# A High-Fat Diet Modifies Brain Neurotransmitter Profile and Hippocampal Proteome and Morphology in an IUGR Pig Model

**DOI:** 10.3390/nu14163440

**Published:** 2022-08-22

**Authors:** Natalia Yeste, Jorge Pérez-Valle, Ana Heras-Molina, José Luis Pesántez-Pacheco, Esteban Porrini, Antonio González-Bulnes, Anna Bassols

**Affiliations:** 1 Departament de Bioquímica i Biologia Molecular, Facultat de Veterinària, Universitat Autònoma de Barcelona, Cerdanyola del Vallès, 08193 Barcelona, Spain; 2 Departamento de Producción Animal, Facultad de Veterinaria, Universidad Complutense de Madrid, Ciudad Universitaria s/n, 28040 Madrid, Spain; 3 Escuela de Medicina Veterinaria y Zootecnia, Facultad de Ciencias Agropecuarias, Universidad de Cuenca, Avda, Doce de Octubre, Cuenca 010220, Ecuador; 4 Departamento de Medicina Interna, Hospital Universitario de Canarias, 38320 La Laguna, Spain; 5 Departamento de Producción y Sanidad Animal, Facultad de Veterinaria, Universidad Cardenal Herrera-CEU, CEU Universities, C/Tirant lo Blanc, 7, Alfara del Patriarca, 46115 Valencia, Spain

**Keywords:** neurotransmitters, metabolism, high-fat diet, hippocampus, intrauterine growth restriction, brain, pig

## Abstract

Intrauterine Growth Restriction (IUGR) hinders the correct growth of the fetus during pregnancy due to the lack of oxygen or nutrients. The developing fetus gives priority to brain development (“brain sparing”), but the risk exists of neurological and cognitive deficits at short or long term. On the other hand, diets rich in fat exert pernicious effects on brain function. Using a pig model of spontaneous IUGR, we have studied the effect on the adult of a long-term high-fat diet (HFD) on the neurotransmitter profile in several brain areas, and the morphology and the proteome of the hippocampus. Our hypothesis was that animals affected by IUGR (born with low birth weight) would present a different susceptibility to an HFD when they become adults, compared with normal birth-weight animals. Our results indicate that HFD affected the serotoninergic pathway, but it did not provoke relevant changes in the morphology of the hippocampus. Finally, the proteomic analysis revealed that, in some instances, NBW and LBW individuals respond to HFD in different ways. In particular, NBW animals presented changes in oxidative phosphorylation and the extracellular matrix, whereas LBW animals presented differences in RNA splicing, anterograde and retrograde transport and the mTOR pathway.

## 1. Introduction

Intrauterine growth restriction (IUGR) is characterized by reduced growth and weight of the fetus, mainly due to the lack of nutrients and oxygen by either maternal nutrient restriction or alterations in the placenta [1]. Worldwide incidence of IUGR ranges between 7 and 15% and accounts for 800,000 neonatal deaths annually [2,3]. The pernicious effects of IUGR extend to the adult life, since the offspring are predisposed to obesity, metabolic syndrome, and diabetes [4,5,6,7,8]. This process is known as ‘prenatal/fetal programming’ or ‘developmental origins of health and disease’ (DOHaD) and was firstly developed by Barker (“Barker hypothesis”) [9]. The mechanisms responsible for the programming of body weight homeostasis are related to epigenetics, in agreement with the “thrifty phenotype” theory [10,11]. Hence, prenatal programming is a protective (adaptive) mechanism ensuring that individuals with poor nutritional conditions during prenatal development modify their metabolic phenotype to enable better use of the available resources during pre- and post-natal life, which are expected to be scarce. When food is abundant postnatally, obesity and other metabolic disorders appear.

The postnatal effects of an exposition to food abundance after an IUGR pregnancy have been largely described in peripheral metabolic tissues but not as much in the nervous system, which has been thought to be preserved due to the brain-sparing phenomenon. Brain sparing means that growth occurs asymmetrically in IUGR, which leads to underdevelopment of the trunk while preserving development of the brain. Such process allows the maintenance of basic functions for the individual’s survival, such as breathing and suckling [12]. Despite this fact, normal development of the brain is not guaranteed, and offspring affected by IUGR can suffer from behavioral disorders related to mobility, cognition, memory, and neurophysiological dysfunctions [13,14,15,16].

Several rodent animal models have confirmed that IUGR provokes alterations in several brain areas [17,18,19]. Nevertheless, these models have significant differences with humans and, consequently, the use of pigs as a biomedical and nutritional model is appreciated due to their similarities in anatomy and physiology [20,21]. One of our groups has developed a well-established porcine model based on a specific breed, the Iberian pig, which has similar evolutive conditions to those previously cited for humans living in developing countries, i.e., adapted genetic specificities for a background of exposure to harsh environments and food scarcity with development of a thrifty genotype. Hence, the Iberian pig is prone to obesity and cardiometabolic diseases in the case of excess nutrients such as a high-fat diet (HFD), therefore it is an animal model which is extensively used in our research [22,23]. In agreement with the DOHaD concept, the fetuses affected by IUGR in our pig model develop prenatal programming, which may modify physiology and metabolism later in life, when the animals are exposed to food abundance for fattening [24,25,26]. Regarding the effects of IUGR on the fetal or neonatal brain, our groups have described alterations in the neurotransmitter (NT) profile in several brain areas [27,28], in the proteome of the hippocampus [29] and the response to supplementation of the maternal diet with the antioxidant hydroxytyrosol [30,31,32].

Alternatively, diets rich in fat exert pernicious effects on brain function. The hippocampus is especially interesting due to its role in learning and memory and, furthermore, the functionality of the hippocampus is affected by energy-rich diets, such as western diets abundant in saturated fat and refined sugars, which have been associated with memory and cognitive decline in aging, and increased incidence of Alzheimer’s disease in humans [33,34]. Animal research has similarly demonstrated impairment of hippocampal function following maintenance on a high saturated fat diet with or without added refined sugar [35,36,37].

The main objective of the present study was to analyze the effects of a long-term high-fat diet (HFD) on the adult brain using a pig model of IUGR caused by placental insufficiency with adequate maternal nutrition, and to answer the question of whether there is an interaction with birth weight, i.e., whether individuals born with a normal birth weight (NBW) or with a low birth weight (LBW, affected by IUGR), respond differently to an HFD when they are adults. The following aspects have been assessed: first, the catecholaminergic and serotoninergic NT profiles in the amygdala, hippocampus, hypothalamus, striatum, and prefrontal cortex, which are brain areas involved in complex functions (appetite, reward, memory, learning, mood, emotion, stress, cognition, etc.); secondly, the hippocampus morphology by using neuronal immunohistochemical markers; and third, the hippocampal proteome.

## 2. Materials and Methods

### 2.1. Ethics Statement

The study was carried out at the INIA animal facilities, which meet local, national, and European requirements for Scientific Procedure Establishments, and was performed according to the Spanish Policy for Animal Protection RD53/2013, which complies with the European Union Directive 2010/63/UE on the care of animals used for research. The experimental procedure was assessed and approved by the INIA Committee of Ethics in Animal Research (report CEEA 2013/036).

### 2.2. Animals and Experimental Procedure

The experiment involved 48 purebred Iberian piglets, which were selected by sex (24 females and 24 males) and birth weight. In this way, half of the piglets were selected within the normal birth weight for the Iberian breed (Group NBW, n = 24, 1.34 ± 0.02 kg of body weight), whilst the remaining were selected as LBW (n = 24, 0.84 ± 0.02 kg of body weight; *p* < 0.0001 versus NBW). There were no significant differences in mean birth weight between males and females within the LBW and NBW groups. Thus, there were four groups (NBW-F, NBW-M, LBW-F, LBW-M), each group with n = 12.

After weaning, at around 28 days of age, all the piglets were housed, sorting out males and females, in collective pens. At the first month after weaning, the piglets were fed with a standard diet with mean values of 18% of crude protein, 4.5% of fat, and 3.35 Mcal/kg of metabolizable energy. Afterwards, from 60 to 140 days of age, the piglets were fed a diet containing mean values of 15.1% of crude protein, 2.8% of fat, and 3.08 Mcal/kg of metabolizable energy; the amount of food offered was re-calculated with age for fulfilling daily maintenance requirements. One female and one male from the group LBW died during this period, so finally 46 pigs were used for the study. Afterwards, the pigs were allocated to different diets from 140 to 385 days old. Half of females and males from the groups NBW and LBW continued being fed with the same diet (maintenance diet; group Ctrl). The remaining pigs, for inducing the expression of obesity, had ad libitum access to the same diet but enriched in fat (6.3%) and, hence, with 3.36 Mcal/kg of metabolizable energy (high-fat diet; group HFD). At 385 days after birth, the pigs were weighed and slaughtered by stunning and exsanguination in compliance with RD53/2013 standard procedures. Subsequently, the head was separated from the trunk at the atlanto-occipital union and the brain was removed from the skull. The amygdala, prefrontal cortex, hypothalamus, hippocampus, and striatum were rapidly dissected, snap-frozen in liquid nitrogen and biobanked at −80 °C until their analyses.

### 2.3. Quantification of Neurotransmitters

Samples were weighed and homogenized by sonication (Branson Digital Sonifier 250, Branson Ultrasonics Corp., Danbury, CT, USA) in a lysis buffer (150 mM NaCl, 50 mM Tris-HCl and 1% NP-40) with 0.3 mg tissue/µL lysis buffer relation. Dihydroxybenzylamine (DHBA) was added to the lysis buffer at 100 pg/µL as internal standard for HPLC. Proteins in brain lysates were precipitated by adding 0.25 M perchloric acid containing 0.1 M sodium metabisulfite and 0.25 M EDTA in a 1.5 (*v*/*v*) ratio. Finally, samples were centrifuged at 12,000× *g* for 10 min at 4 °C and kept at −80 °C until analysis.

Concentrations of catecholamines (NA, DA, DOPAC and HVA) and indoleamines (5-HT, 5-HIAA) were determined by HPLC (EliteLaChrom, Merck-Hitachi, Prague, The Czech Republic) equipped with a Cromolith Rp-18e column (Merck, Darmstadt, Germany) with electrochemical detection (ESA Coulochem II 5200, Thermo Fisher Scientific, Whaltham, MA, USA). The mobile phase consisted of 0.05 M citrate buffer pH 2.8, 0.05 mM EDTA, 1.2 mM sodium octyl sulphate (SOS), and 1% acetonitrile. The applied voltage was set at 0.4 mV and the flow rate was 1.2 mL/min. All procedures are described in detail by Arroyo et al. [38].

### 2.4. Immunohistochemical Analysis of the Hippocampus

LBW individuals were matched with NBW individuals. Litter, sex, and weight were considered when choosing the NBW piglets. Therefore, from the same mother, another individual of the same sex with the highest weight was chosen. Using these selection criteria, 12 animals from the Ctrl group were obtained: 6 LBW (3 females and 3 males) and 6 NBW (3 females and 3 males); and 12 animals from the HFD group: 6 LBW (3 females and 3 males) and 6 NBW (3 females and 3 males).

Hippocampal samples were frozen in an OCT medium (Aname, Madrid, Spain) using molds, an isopentane bath (Sigma, St. Louis, MO, USA), and dry ice, controlling the freezing temperature between −40 °C and −60 °C. The OCT blocks were cut with a cryostat (SME Cryotome Thermo Electron Corporation, Thermo Scientific, Braunschweig, Germany) into 40 μm thick sections in a longitudinal orientation, collecting them in flotation with an antifreeze solution pH 7.4 (40% ethylene glycol, 30% glycerol, and 30% phosphate buffer 0.1 M pH 7.4).

For immunohistochemistry, a minimum of 6 sections per individual were analyzed. Sections were washed using a phosphate buffer 0.1 M pH 7.4, and endogenous peroxidase activity was blocked using 1% H_2_O_2_. Sections were blocked with 2% normal goat serum (NGS) and incubated with the corresponding primary antibodies with NGS overnight at 4 °C. The antibodies used were raised against NeuN (1:1000, Mouse monoclonal anti-neuronal nuclei; Merck Millipore, Chemicon, Billerica, MA, USA, Ref. MAB377), doublecortin (DCX, 1:750, Rabbit polyclonal anti-doublecortin; Abcam, Cambridge, MA, USA, Ref. ab18723), and neurofilaments (NFT, 1:10,000, Mouse monoclonal anti-neurofilament 200; Sigma, St. Louis, MO, USA, Ref. N0142). Afterward, the sections were washed and incubated with biotinylated goat secondary antibodies (1:500, anti-mouse IgG or anti-rabbit IgG; Agilent Technologies, Dako, Glostrup, Denmark). Next, sections were incubated with an avidin–biotin–peroxidase complex (Standard ABC Peroxidase Staining Kit; Pierce Biotechnology, Rockford, IL, USA) and revealed with 3,3′-diaminobenzidine tetrahydrochloride (DAB Liquid Substrate System; Sigma, St. Louis, MO, USA). Sections were transferred to Superfrost Plus™ adherent slides, counterstained with hematoxylin, and mounted in resinous DPX mounting medium (Sigma, St. Louis, MO, USA).

### 2.5. Image Processing and Analysis

Slides were digitally scanned with 2.0 HT Nanozoomer (Hamamatsu Photonics, Hamamatsu, Japan) at the Histopathology Service of the Biomedicine Research Institute (IRB, Barcelona, Spain). The scanned images were visualized and analyzed using NDP.view 2 software (Hamamatsu Photonics, Hamamatsu, Japan). 

NeuN immunostaining was analyzed using ImageJ 1.52p (Version 1.53s, NIH, Bethesda, MD) free software from the website of the National Institutes of Health. The procedure performed to analyze the images was: (1) convert scanned color image to grayscale (8-bit); (2) set measurement scale; (3) threshold the image using “Make Binary”, and (4) analyze particles. The whole tissue area, neuron area, and neuron area percentage were calculated. To obtain the level of the specific DAB signal on the whole tissue in the photograph, the actual area of neurons was calculated by subtracting the blank areas that contained no tissue (e.g., lumina of vessels and artefacts). Moreover, the DAB-positive area outside the neuron area was excluded. Individual neuron clusters were numbered to obtain information regarding a particular neuron (size, circularity, area, etc.) from the tabulated results.

### 2.6. Statistical Analyses

All statistical analyses were performed in SPSS 24.0 software (IBM, Chicago, IL, USA). The significance level was established at *p* < 0.05 and a tendency was considered at 0.05 ≤ *p* ≤ 0.1. Descriptive data are presented with the means and the standard error (mean ± SE). Normal distribution of the variables was confirmed with a Kolmogorov–Smirnov test. Whenever possible, data were log transformed to correct the distribution and hence permit the use of parametric statistics. Normally distributed measures were analyzed using the UNIANOVA procedure of SPSS with Tukey adjustment. In all models, each pig was introduced as the experimental unit, the fixed effects included were diet (CTRL and HFD), birth weight (NBW and LBW), sex (male and female), and their interactions. In addition, pairwise comparisons with Bonferroni adjustment were also performed for significant interactions.

### 2.7. Proteomic Analysis by Isobaric Mass Tag Labeling with TMT10plex™

The same twenty-four individuals from the IHC analysis were included (Yeste et al., 2021a), using the companion hippocampus. Thus, the distribution of samples was twelve from the Ctrl group (6 NBW and 6 LBW, from both males and females), and twelve from the HFD group (same distribution). Protein extracts were prepared in 400 µL of 150 mM NaCl, 50 mM Tris-HCl pH 7.5, and 1% NP-40. Protein quantification was performed using PierceTM 660 nm Protein Assay (#22662) and Ionic Detergent Compatibility Reagent (#22663). Trypsin digestion was performed following the FASP protocol (Wiśniewski et al., 2009). After digestion, samples were acidified with trifluoroacetic acid solution, followed by desalting and cleaning with PolyL C18. 70 μg of protein for each sample was dried, reconstituted in 100 µL of 100 mM TEAB (triethyl ammonium bicarbonate), and labelled with TMT10plex (Thermo Scientific, Braunschweig, Germany) in three reactions, accounting for all individual samples plus three pools as internal controls. The experimental design for the TMT10 labeling is shown in Supplementary Table S1.

### 2.8. Nanoliquid Chromatography Electrospray Ionization Tandem Mass Spectrometry (nanoLC-ESI-MS/MS)

After labeling, samples were combined, desalted, and cleaned with PolyLC C18 and PolySCXn and resuspended in 1% formic acid in 3% acetonitrile prior to MS analysis. LC-MS coupling was performed with the Advion Triversa Nanomate (Advion BioSciences, Ithaca, NY, USA) as the nanoESI source performing nanoelectrospray through chip technology. Peptides were loaded directly onto the analytical column and were separated by reverse-phase chromatography using a NanoEase MZ HSS T3 column (75 µm × 250 mm, 1.8 µm, 100 Å, Waters). Chromatographic gradients started at 99% buffer A (0.1% formic acid in H_2_O) and 1% buffer B (0.1% formic acid in acetonitrile) with a flow rate of 250 nL/min and gradually increased to 35% buffer B in 270 min and then to 50% buffer B in 5 min. After each analysis, the column was washed for 10 min with 15% buffer A and 85% buffer B.

The Nanomate was attached to an Orbitrap Fusion Lumos™ Tribrid mass spectrometer and operated at a spray voltage of 1.7 kV and a delivery pressure of 0.5 psi in positive mode and source temperature at 275 °C. In each data collection cycle, one full MS scan (400–1600 m/z) was acquired in the Orbitrap (1.2 × 105 resolution setting and automatic gain control [AGC] of 2 × 105). The following MS2-MS3 analysis was conducted with a top-speed approach. The most abundant ions were selected for fragmentation by collision-induced dissociation (CID). CID was performed with a collision energy of 35%, 0.25 activation Q, an AGC target of 1 × 104, an isolation window of 0.7 Da, a maximum ion accumulation time of 50 ms and turbo ion scan rate. Previously analyzed precursor ions were dynamically excluded for 30 s.

For the MS3 analyses for TMT quantification, multiple fragment ions from the previous MS2 scan (SPS ions) were co-selected and fragmented by HCD using a 65% collision energy and a precursor isolation window of 2 Da. Reporter ions were detected using the Orbitrap with a resolution of 60,000, an AGC of 1 × 105 and a maximum ion accumulation time of 120 ms.

### 2.9. Database Searching

Database searches were performed with Proteome Discoverer v2.5.0.400 software (Thermo Scientific, Braunschweig, Germany) using Sequest HT search engine and UniProt_PIG_2021_02 and contaminants. A search was run against targeted and decoy database to determine the false discovery rate (FDR). Search parameters included trypsin, allowing for two missed cleavage sites, carbamidomethyl in cysteine and TMT 6plex peptide *N*-terminus as static modification and TMT 6plex in K, methionine oxidation and acetylation in protein *N*-terminus as dynamic modifications. Peptide mass tolerance was 10 ppm for MS1, 0.6 for the MS2 and 20 ppm for the MS3. Peptides with a q-value lower than 0.1 were considered as positive identifications with a high confidence level.

### 2.10. Quantitative Analysis

TMT reporter ion intensities were used for protein quantification. Unique peptides (peptides that are not shared between different protein groups) were considered for further quantitative and statistical analysis. Within each TMT experiment, peptide quantitation was normalized by summing the abundance values for each channel over all peptides identified within an experiment. The channel with the highest total abundance was taken as a reference and all abundance values corrected in all other channels by a constant factor per channel, so that at the end the total abundance is the same for all channels. Protein quantitation was done by summing all peptide normalized intensities for a given protein. Sample C1.1 was removed from the analysis because it had a lower amount of protein compared to the others and it was considered an outlier. All the batches were normalized using quantile normalization.

Data were first transformed to log scale to apply a linear model, and then filtered to retain only proteins with valid quantification values in at least 3 valid values in at least one group (group here refers to diet + weight + sex). Missing values were imputed with normally distributed random numbers (centered at −1.8 standard deviations units and spread 0.3 standard deviations units with respect non missing values). To adjust for batch effect a linear model was used with TMT batch as fixed effect. Model fitting was accomplished with the lmFit function of the limma package [39], of R statistical software [R Core Team. (2014). R: A Language and Environment for Statistical Computing. Available online at: http://www.Rproject.org, accessed on 24 June 2022].

For each comparison, estimated fold changes and *p*-values were calculated. Finally, *p*-values were adjusted using the Benjamini & Hochberg correction. Proteins with an adjusted *p*-value lower than 0.05 and fold change higher than 1.5 were considered statistically significant between groups.

### 2.11. Gene Ontology and Bioinformatic Analysis

Venn diagrams were drawn using FunRich (www.funrich.org, accessed on 24 June 2022). For protein names and Gene Ontology (GO) classifications, PANTHER version 16.0 software (http://pantherdb.org/, accessed on 24 June 2022) (Mi et al., 2017) was used together with the UniProt databases (http://www.uniprot.org/, accessed on 24 June 2022). Complete GO and GO slims were run. GO slims are cut-down versions of the GO ontologies containing a subset of the terms in the whole GO. They provide a broad overview of the ontology content, but excluding the details of the specific fine-grained terms (gene.ontology.org). For pathway analysis, the Reactome platform version 77 was used (https://reactome.org/, accessed on 24 June 2022) [40], as well as the Kegg Mapper tool version 5.0 (https://www.genome.jp/kegg/mapper.html, accessed on 24 June 2022) [41]. For protein interaction network analyses, identified proteins were analyzed with STRING version 11.5 (http://string-db.org/, accessed on 24 June 2022) [42].

The MS proteomics data has been uploaded to the ProteomeXchange Consortium via the PRIDE partner repository [43], with the dataset identifier PXD032300.

## 3. Results

### 3.1. Effects of High-Fat Diet and the Influence of IUGR on the Neurotransmitter Profile in Several Brain Areas

Results are shown in Table 1 and Supplementary Table S2. HFD has an important effect on the serotoninergic pathway, since an increase in 5-HT and in total indoleamines is observed in all analyzed brain areas (hippocampus, amygdala, prefrontal cortex, hypothalamus, and striatum). In the hypothalamus, the effect of HFD is also observed in DA. In the hippocampus and the striatum, an increase in DOPAC, a DA metabolite, caused by HFD is also observed. There is neither a relevant effect of the birth weight nor an interaction between diet and birth weight in the hippocampus, amygdala, prefrontal cortex, and hypothalamus. In the striatum, LBW individuals had higher values than NBW for DA and its metabolite 3-MT (and hence, in total dopaminergic). Noradrenalin, glutamate, and GABA were also determined in the five brain areas, but there were no differences due to diet, weight nor their interaction.

The statistical comparisons by diet, birth weight, or sex are shown in Supplementary Table S2. There is no influence of birth weight in any case, except for DA and its metabolite 3-MT in the striatum (higher concentration in the LBW group). Regarding the effect of sex, there were not relevant differences, but glutamate was higher in males in the hippocampus and prefrontal cortex, and higher in females in the hypothalamus. HVA was higher in males in the prefrontal cortex. Finally, NA was higher in the striatum of males.

### 3.2. Effects of High-Fat Diet and the Influence of IUGR on the Morphology of the Hippocampus

**Figure 1:**
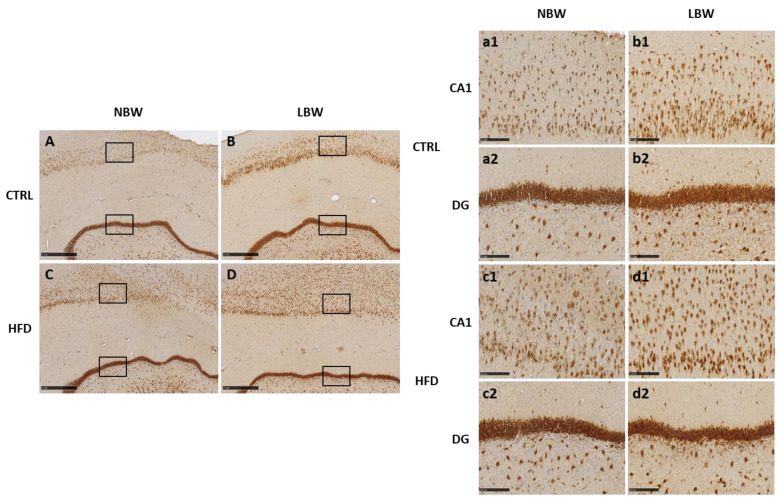
NeuN immunostaining on the hippocampus of one year-old pigs born with normal birth weight (NBW) or low birth weight as result of IUGR (LBW) and fed a control diet (Ctrl, **A**,**B**) or an HFD diet (**C**,**D**). Representative images show the mature neurons immunostained with the NeuN antibody. Panels are magnifications of the CA1 (**a1**–**d1**) and DG (**a2**–**d2**) areas shown using black boxes. Scale bars: 1000 µm (**A**–**D**), and 250 µm (**a1**–**d1**, **a2**–**d2**).

As it can be seen in Table 2 and Figure 1, differences due to IUGR and HFD are mild. Staining with NeuN as a marker of mature neurons, LBW animals showed a higher number of mature neurons than NBW animals, but neuronal size was similar in both weight groups. After a long term HFD, the mean size and the corresponding area percentage increased but only in the NBW group. There were no main differences in the GD area in number of NeuN-positive neurons and their mean size due to the birth weight or to the diet. Nevertheless, HFD increased the area of GD neurons and, consequently, the GD width was narrower in these groups, regardless of their birth weight.

**Figure 2:**
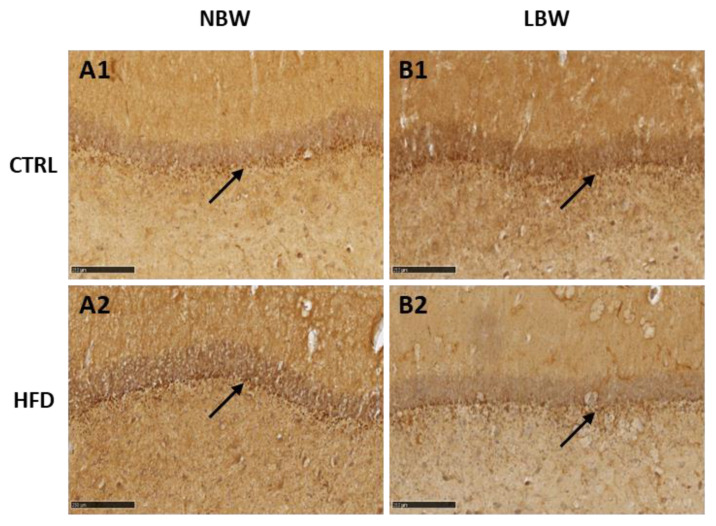
DCX immunostaining on the DG of the hippocampus of one year-old pigs born with normal birth weight (NBW, (**A**)) or low birth weight as result of IUGR (LBW, (**B**)) and fed a control diet (Ctrl) or an HFD diet (identified as 1 or 2, respectively). Representative images show the immature neurons immunostained with the DCX antibody. Scale bars: 250 µm.

With DCX staining, a weak labeling was observed only in DG (Figure 2). The high background prevented the quantification of DCX-positive neurons, but a milder labeling was observed in LBW animals fed with HFD, in opposition to the results obtained with NeuN staining. With NFT, a strong labeling of the neuropil was observed, but no differences due to birth weight or treatment were found (not shown).

### 3.3. Effects of High-Fat Diet and the Influence of IUGR on the Proteome of the Hippocampus

A total of 56413 peptide spectrum matches corresponding to 23,128 peptides and 3508 proteins were identified in the TMT analysis. From these, 2736 proteins could be quantified. Uncharacterized proteins were identified by homology (>98%) with other mammalian databases.

The pair comparison between birth weight (NBW vs. LBW), diet (Ctrl vs. HFD), and sex (M vs. F) yielded very few differences between conditions: 13 DAPs were identified related to weight, 9 DAPs to diet and 10 DAPs to sex (Supplementary Table S3). There were no common proteins identified in two or more comparisons. Furthermore, the DAPs did not show any functional relationship between them in the bioinformatics analysis. In consequence, the simple comparative analysis was dismissed.

**Figure 3:**
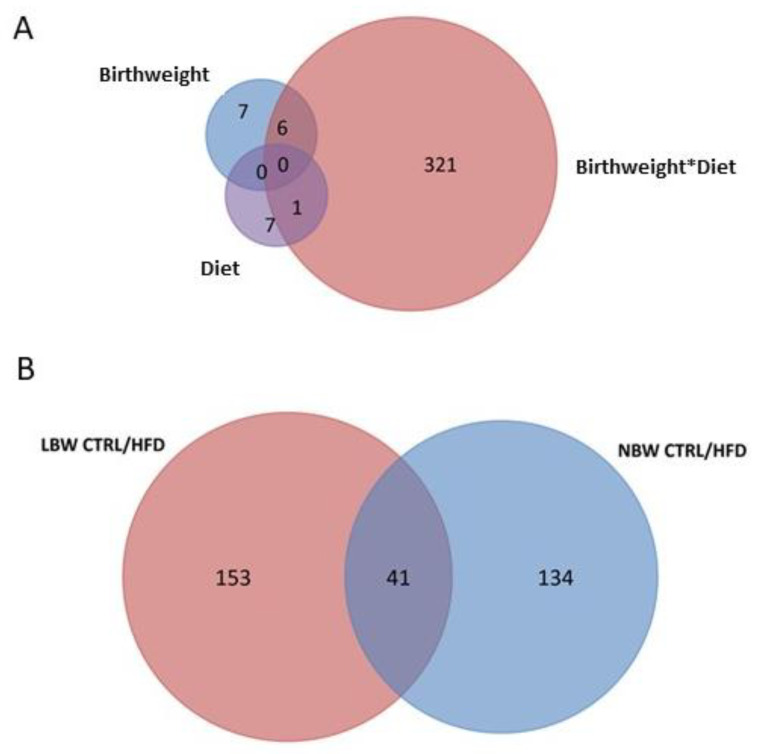
Venn diagram of differentially abundant proteins in the hippocampus of pigs. (**A**) Number of proteins identified by birth weight, diet and its interaction (Birthweight*Diet). (**B**) Number of proteins identified by comparing HFD vs. Ctrl in NBW and LBW groups.

The question to answer was whether NBW- and LBW-born individuals responded differently to an eight-month HFD feeding when they were adults (from 140 to 385 days-old). To answer this question, the comparison between identified proteins was performed between NBW-Ctrl vs. NBW-HFD, and between LBW-Ctrl vs. LBW-HFD. In these comparisons, 328 DAPs were identified. Amongst them, six had been previously found in the comparison regarding birth weight and only one in the comparison regarding diets (Figure 3A). From the 328 DAPs, 194 were identified when comparing LBW-Ctrl and LBW-HFD, whereas 175 were identified in the comparison NBW-Ctrl vs. NBW-HFD (Supplementary Table S4). Forty-one proteins were common to both comparisons (Figure 3B).

**Figure 4:**
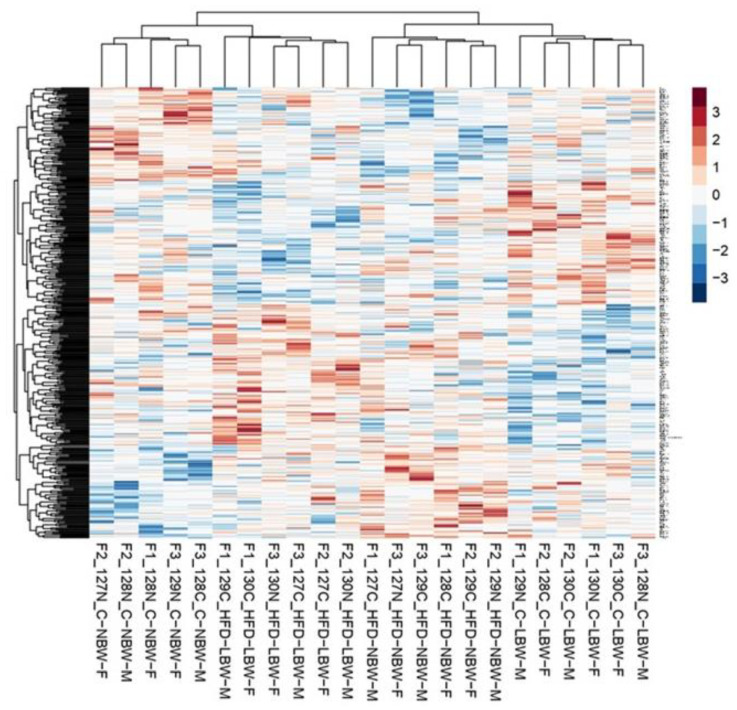
Heatmap of differentially abundant proteins in the hippocampus of pigs in the four analyzed groups (NBW subjected to a control diet (C) or HFD; LBW subjected to a control diet (C) or HFD). Sex of the individual (M, F) is indicated. Numbers indicate the TMT labeling reagent and F indicates the TMT labeling reaction.

The heatmap readily showed that the individuals in each group clustered together and that the proteomic response to HFD of NBW individuals differed from LBW individuals (Figure 4). On the contrary, the effect of sex was not relevant.

#### 3.3.1. Gene Ontology Analysis

**Figure 5:**
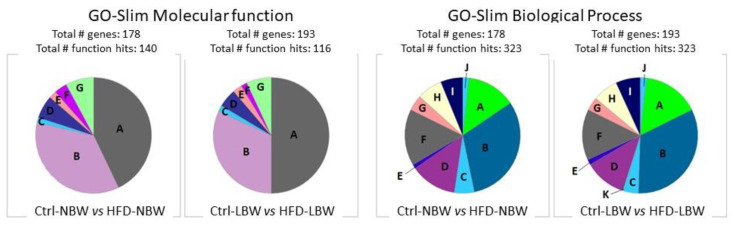
Functional classification of differentially abundant proteins identified in pigs comparing the effect of an HFD diet in NBW and LBW animals by Slim-GO analysis of the Molecular function and biological processes ontologies. (left hand) Molecular Function: A, binding; B, catalytic activity; C, molecular adaptor activity; D: molecular function regulator; E, molecular transducer activity; F: structural molecule activity; G, transporter activity. (right hand) Biological Process: A, biological regulation; B, cellular processes; C, developmental process; D, localization; E, locomotion; F, metabolic process; G, multicellular organismal process; H, response to stimuli; I, signaling; J, biological adhesion; K, growth.

The GO analysis did not show large differences in the distribution of molecular function, biological processes, and cellular components between NBW and LBW animals subjected to Ctrl and HFD diets (Figure 5). Mainly, the percentage of proteins related to binding was larger in the LBW group, whereas the percentage of proteins with catalytic activity was larger in the NBW group (molecular function), whereas no main differences were seen in the biological process analysis. The detailed GO analysis allowed also to identify the main categories for biological process, molecular function, and cell components in both groups of animals after response to an HFD. As shown in Table 3, several categories were common to NBW and LBW groups, such as vesicle-mediated transport or cadherin binding, but other appear specifically in one of the groups. Thus, oxidative phosphorylation/ATP metabolic process was characteristic of NBW animals, whereas mRNA splicing was unique to LBW animals. In the molecular function, enrichment for mRNA binding was higher in LBW animals. Correspondingly, cellular component categories related to vesicle transport and synapsis were enriched in both groups, whereas the cytochrome and respiratory-chain complexes were present only in NBW animals. The complete GO analysis is presented in Supplementary Table S5.

#### 3.3.2. KEGG Pathway Analysis

The KEGG pathway analysis was performed for NBW and LBW animals subjected to Ctrl and HFD diets to assess whether the birth weight influenced the response to an HFD. Many of the pathways were common to both groups but some interesting differences appeared. As shown in Table 4, the main common pathways involved in the response to diet were metabolic pathways, thermogenesis, autophagy, focal adhesions, insulin and cAMP-signaling pathways, and lysosome and protein processing in the ER. In metabolic pathways, proteins regulated in the NBW group were essentially related to oxidative phosphorylation whereas those appearing in the LBW groups were mainly related to carbohydrate and lipid metabolism. Unique pathways in the NBW group were oxidative phosphorylation and MAPK signaling pathway, whereas unique pathways in the LBW group were spliceosome, and the mTOR signaling pathway. The complete KEGG pathway analysis is shown in Supplementary Table S6.

#### 3.3.3. Pathway Analysis with Reactome

**Figure 6:**
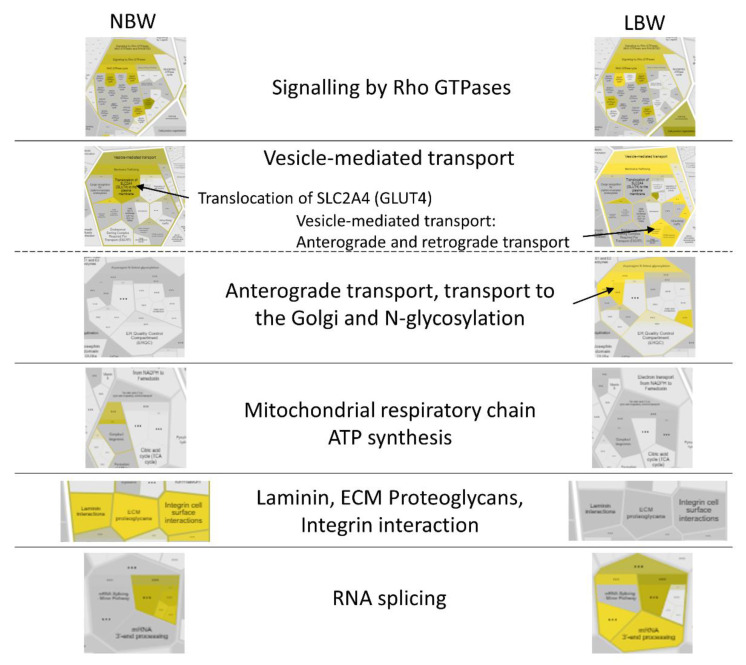
Voronoi diagram sections obtained with Reactome showing the main pathways regulated by an HFD in NBW and LBW groups. Intensity of yellow color indicates abundance of DAPs in a specific pathway. The complete Voronoi picture and images with enlarged letter size is shown in Supplementary Figure S1.

The analysis with Reactome allowed to visualize the similarities and differences of the pathways involved in NBW and LBW individuals. In Figure 6, the Voronoi diagram obtained for NBW and LBW groups in response to an HFD showed that some pathways are quite similar to both groups (as signaling by Rho GTPases). Vesicle-mediated transport appeared to be regulated in both groups, but differences exist in the pattern. Other pathways appeared to be mainly regulated in one of the groups, confirming the results obtained with other bioinformatic tools. Thus, in NBW individuals, a differential regulation is observed for mitochondrial respiratory chain and ATP synthesis, and signal transduction from the extracellular matrix (ECM, Integrins, linkage to MAPK pathway). Conversely, in LBW animals the major differential pathway was the anterograde transport, transport to the Golgi and *N*-glycosylation, and RNA splicing. The complete Voronoi picture is shown in Supplementary Figure S1.

#### 3.3.4. Upregulated and Downregulated Proteins

The next step was to analyze whether the pathways that showed differences after the HFD diet between both groups were up- or down-regulated. Overall, in NBW animals oxidative phosphorylation is increased and several extracellular matrix proteins are increased, whereas in the LBW group mRNA splicing seems to be up-regulated as well as the mTOR pathway (Table 5). Similar conclusions were reached after network analysis with String (Supplementary Figure S2).

## 4. Discussion

The hypothesis of the present work was that individuals which were LBW at birth due to IUGR during gestation would respond to an HFD when adults in a different way than their NBW siblings. This would be related to the thrifty phenotype model (Barker hypothesis), which states that fetuses adapt to a deficient environment through reprogramming and that they retain this phenotype postnatally, even when the environment (nutrition) is favorable [9,11]. This statement has been widely studied from the metabolic standpoint and in metabolic tissues, such as liver, adipose tissue, and muscle, but there are no references regarding the central nervous system. Nevertheless, the influence of IUGR in the development of the nervous system is important and, although most data come from studies performed in children and young people (related to cognitive performance and other pathologies) there are also indications of IUGR being a risk factor for neurodegenerative diseases in the adult [44,45]. Alternatively, a high-fat diet has been associated with cognitive deficits and an increased risk of neurodegenerative diseases, due to inflammation and brain aging [33,34,46,47,48,49,50].

The Iberian pig is an adequate animal model to study this phenomenon, which has been previously characterized by our groups [24,26]. In the present work, the porcine IUGR model does not include a caloric restriction during gestation, and it is based on the large litters in this animal species that provokes the birth of low-birth-weight piglets in each litter, that is the result of a spontaneous IUGR due to placental insufficiency. In this experimental design, NBW and LBW individuals were born from the same mothers and there was no influence of the “mother” variable in the statistical analysis of the results.

### 4.1. Effect of an HFD Diet in NBW and LBW Pigs in the NT Profile and in the Morphology of the Hippocampus

A high-fat diet had a clear and selective influence on the neurotransmitter profile in all studied brain areas (hippocampus, amygdala, prefrontal cortex, hypothalamus, and striatum). Specifically, the concentration of 5-HT increased in all five brain areas and, therefore, there was also an effect on total indoleamines. The increase in 5-HT occurred in both NBW and LBW animals since there were no significant interactions between both factors.

Serotonin has a widely accepted anorexigenic action on the CNS, that is, it decreases food intake [51,52]. The hypothalamus and the hippocampus are the central areas controlling food intake since they influence, respectively, the homeostatic (related to energy requirements) and the hedonic (motivational) appetite. Nevertheless, since food intake is a very complex behavior, it also depends on other brain areas such as the striatum, the amygdala, and the cortex, which influence appetite due to their role in reward and emotions [53,54]. The general increase in 5-HT observed in all analyzed areas may contribute to the satiating effects of an HFD. In the hypothalamus, DA is considered also to have an anorexigenic role [51,55]. Since DA was also increased in HFD-fed animals in this brain area, it could further contribute to the satiating effects of an HFD.

On the other hand, there were no relevant effects of IUGR on the NT profile since NBW and LBW did not present relevant differences except for a higher concentration of DA and its metabolite 3-MT in the striatum of NBW pigs. Since the striatum is mainly involved in rewarding, this difference may account for a different feeding behavior in these animals. Nevertheless, there was almost no effect of birth weight in the NT profile in adult animals and this may correspond to the brain-sparing phenomenon that happens in IUGR. Our previous results did find an effect of IUGR on the NT profile in 100-day old fetuses [30], which is therefore compensated in adult animals.

Regarding the morphology of the hippocampus studied by IHC using NeuN as marker for mature neurons and DCX as marker for immature neurons, the differences induced by an HFD were scarce. We have studied the hippocampus due to its important role related to appetite, memory processes, cognitive functions, learning capacities, and motor skills, which are essential for normal neurological development [56]. Furthermore, several studies including humans indicate that the hippocampus is very vulnerable to hypoxia, malnutrition, and altered micronutrient supply, which are present in IUGR [57]. Our previous work has demonstrated that IUGR alters the morphology of the hippocampus in 100-day old pig fetuses [30]. In that article, we found that there is a neuronal deficit in the CA1 and DG areas of the hippocampi of LBW fetuses (affected by IUGR), after labeling with NeuN and NFT antibodies. Inversely, DCX labeling suggested that LBW fetuses have a higher number of immature and disorganized neurons than NBW fetuses. Thus, our results indicated that cell differentiation proceeds more slowly in LBW than in NBW animals at the fetal age.

In the present work, we aimed to study whether these changes are still visible in adult life. Our results indicate that an HFD does not provoke relevant changes in the morphology of the hippocampus. The observed effects in CA1 and DG (a mild increase in the percentage of the area occupied by neurons) were observed in NBW and LBW groups, that is, independently of the initial birth weight. Alternatively, an effect of IUGR was observed since the number of NeuN-positive neurons was higher in LBW pigs. Again, it should be emphasized that the analysis was performed in adult animals, whereas IUGR was suffered during gestation. After birth, both groups of animals (NBW and LBW) were treated and fed under the same conditions. This result means that, at least in this aspect, the effects of the IUGR remain further after birth. It is interesting to note that we had previously found the opposite effect of IUGR in fetuses, since LBW animals presented a lower number of mature neurons than NBW fetuses [30]. It may be speculated that neuronal development is slower during the fetal period in animals subjected to IUGR, but the proliferation/differentiation rate catches up afterwards and it is maintained even at adult ages.

### 4.2. Effect of an HFD Diet in NBW and LBW Pigs in the Proteome of the Hippocampus

The heatmap showing the response of all animals included in the study clearly showed that the five animals in each group clustered together, indicating a relatively low inter-individual variability (Figure 4). Furthermore, each group separated from the others, indicating that indeed the treatment with an HFD had an effect on the hippocampus proteome (even if the direct comparison between Ctrl and HFD groups did not show relevant differences). Similarly, NBW-Ctrl and LBW-Ctrl groups also showed different patterns indicating that some differences exist in the proteome composition due to IUGR, even if they were not detected in the simple analysis comparing global NBW versus LBW. On the contrary, sex did not have an influence since males and females appeared to be mixed in each of the groups. In a previous work of our group performed in a different porcine model of IUGR (mothers subjected to caloric restriction during gestation) in 100-day old fetuses instead of adult pigs, few differences comparing severe and mild IUGR were found in the hippocampus proteome (only six proteins) [29].

To answer the question of whether birth weight (i.e., to have suffered IUGR during the fetal period) may influence the changes in the hippocampus proteome in response to an HFD in adulthood, the comparison was performed between NBW-Ctrl vs. NBW-HFD, and between LBW-Ctrl vs. LBW-HFD. In these comparisons, 328 DAPs were identified. First, the set of common proteins regulated in NBW and LBW animals were analyzed. In the response to an HFD diet, 41 proteins out of 328 DAPs were common to both NBW and LBW groups. Other 36 proteins were members of similar families (i.e., ACAD10 and ACAD8), meaning that 77 proteins out of 328 were common (Supplementary Table S7). According to this idea, the analysis performed with various bioinformatics tools (GO, KEGG, Reactome) indicated that several elicited pathways are common to NBW and LBW groups (Thermogenesis, Autophagy, Focal adhesion, Insulin signaling, cAMP signaling, Lysosome) (Table 3). Likewise, the analysis with only the 77 common proteins included mainly in vesicle-mediated transport, signal transduction by growth factor receptors and second messengers, Rho-GTPase cycle, transport to the Golgi, extracellular matrix organization, HSP90, and Complex I biogenesis. This similar response of NBW and LBW groups to HFD was expected since the animals were 1-year old adult animals, and they had lived in the same conditions, had the same diet, and had been paired by the litter for the proteomic analysis. A high number of differences between both groups would have been unexpected. Nevertheless, some significant differences in the response to the HFD were found between both groups, which will be due to the different birth weight, that is, to the IUGR condition (Supplementary Tables S5–S7).

Metabolism and signal transduction are modulated in NBW and LBW groups after an HFD diet, but with some differences. It has to be noted that the literature on the effects of an HFD on the proteome is quite abundant in peripheral tissues as liver, muscle, and adipose tissue [58,59,60], but scarce for the nervous system, and specifically for the hippocampus. In the hypothalamus, a short-length HFD induces changes indicative of cellular stress, altered synaptic plasticity, and mitochondrial function in mice [61]. It also induces changes in the phosphoproteome of the brain, especially of proteins involved in neuronal development, in vesicle trafficking, and in cytoskeletal functions [62]. Interestingly, there is one study performed in rats subjected to a high saturated fat and refined sugar diet for eight weeks where the proteome of the hippocampus was analyzed by label-free shotgun proteomic analysis [63]. The authors found main effects on the citrate cycle and oxidative phosphorylation, structure of the cytoskeleton, calcium-dependent signal transduction, synaptic vesicles, and ubiquitination. The effect on energy metabolism was a decrease in oxidative phosphorylation. In our case and in NBW animals, we have found an increase in several components of the respiratory chain (UQCRC2, COX15, COX7A2, COA1, NDUFS5, and NADH1) but a decrease in another (COX6B1) and also in one of the subunits of ATP synthase, the enzyme ultimately responsible for ATP synthesis (ATP5PD) with an uncertain global outcome. The differences between the Francis et al. study and ours may be due to the diet, which in that case was a high-saturated fat high-refined sugar which is believed to be more deleterious than high fat only [37], the duration of the diet, or the animal species. The increase in mitochondrial respiratory chain proteins may indicate an increase in the energy nutrients that arrive to the brain. These differences are more evident in NBW animals, which therefore seem to have a metabolism more based on oxidative phosphorylation than LBW animals. That will agree with a catabolic use of nutrients (i.e., the extra fat given in the diet), whereas the LBW animals would probably direct the extra fat to storage.

Especially in NBW animals, the extracellular matrix is modulated. Overabundant proteins after an HFD include agrin, a proteoglycan present in neurons involved in dendritic filopodia and synapse formation, collagen is an extracellular protein that may interact with integrins like ITGA7, which may bind to parvin by interacting with ILK (integrin-linked protein kinase), SAPRCL1 is an extracellular protein involved in synaptic membrane adhesion. Thus, a signaling node appears that may be activated especially in NBW neurons and that, overall, may influence the neuronal and synaptic function [64,65,66].

Regarding the pathways that appear to be more influenced by an HFD in LBW animals, it is interesting to note the abundance of proteins involved in the mRNA splicing node. An HFD has been recently found to induce, in the hypothalamus, the formation of new alternative polyadenylation sites, a molecular feature that dictate the fate of newly synthesized RNA molecular and direct alternative splicing of nascent transcripts [67]. Our results will support this fact. The consumption of diets high in fat have been linked to reduced cognitive function and an increased risk of neurodegenerative diseases as Alzheimer’s disease (AD) [68]. AD is characterized by the formation of insoluble neurofibrillary tangles formed by accumulation of the Tau protein. In a mouse model of AD, it has been described that the splicing of Tau is modified by an HFD [69]. It is interesting to speculate that birth weight could be another variable influencing the risk of AD. Although most of the studies on the neurological consequences of IUGR have focused on children and young people, there are some data in the literature indicating that IUGR may also be involved in pernicious neurological outcomes in the adult, including AD [44,45].

Vesicular transport is regulated in NBW and LBW groups although there are more proteins and reactions identified in the LBW group. These DAPs may be up- or down-regulated in HFD-fed animals and indicate a higher rate of organelle functioning. A number of proteins involved in anterograde and retrograde transport (that is, axonal transport from the cell body to the synapsis or from axon termini toward the cell body, respectively) have been also identified [70,71]. This may be related to autophagy or lipid droplets, which would be expected to be down regulated in HFD-fed animals since they will not need an extra source of energy nutrients. It is interesting to note that, especially in LBW animals, these changes in ER-Golgi proteins are accompanied by an increase in enzymes involved in glycosylation of proteins (GFPT1, GMPPA and PMM1).

**Figure 7:**
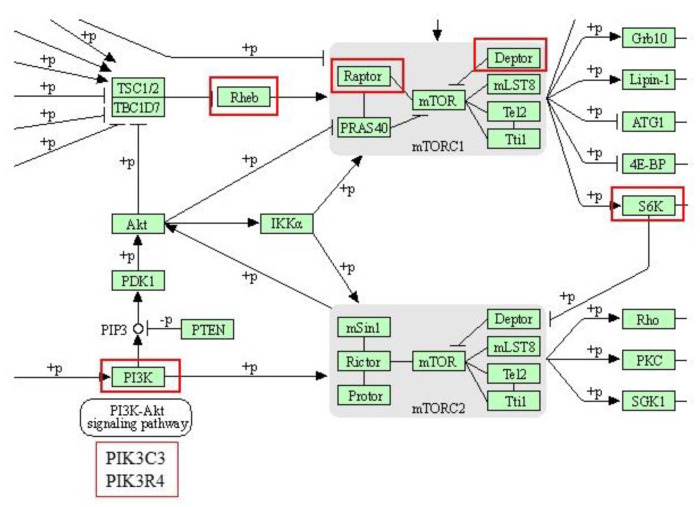
KEGG diagram for the mTOR pathway (hsa04150) with the differentially abundant proteins identified in LBW animals subjected to an HFD marked by red squares.

Finally, the mTOR pathway has a central role in the sensing of the cell to nutrient availability. It is activated in high-energy status (nutrient abundance), and phosphorylation of the S6 ribosomal proteins and activation of protein synthesis occurs as its final step. This pathway appears to be upregulated only in LBW animals with several proteins corresponding to this pathway (PIK3C3, PIK3R4, DEPTOR, RPTOR, and RPS6KA3) including one of the subunits of S6kinase (Figure 7). It has been described that IUGR causes an inactivation of the mTOR pathway leading to a “hypometabolic status” in the fetus and the placenta that corresponds to an adaptive strategy for survival. Inactivation of the mTOR pathway will be a major mechanism underlying the low birth weight of IUGR piglets [72,73]. In this work, LBW animals after HFD have an up-regulated mTOR pathway, which would probably be the consequence of the thrifty phenotype: although the animals do not have any nutritional restriction, their metabolism is permanently on alert to favor anabolic reactions even in the presence of nutrients also in the brain. As it has been described in the Introduction, this fact is well characterized regarding fat and carbohydrate metabolism. Our studies indicate that it may also be extended to protein synthesis.

Even if we observed changes in the NT profile, almost no proteins directly related to neurotransmission were identified in the proteomic analysis. Although it may be surprising, it has to be taken into account that the rate-limiting enzymes for DA and 5-HT metabolic pathways (that is, tyrosine hydroxylase, and tryptophan hydroxylase) are mainly regulated by phosphorylation and allosteric effectors and not through changes in the amount of enzyme protein [74,75]. Nevertheless, we have identified many proteins involved in signal transduction, for example, DAPs corresponding to the cAMP signaling pathway, which is the transduction mechanism for several 5-HT receptors, which belong to the GPCR family [76], and also DA [77]. Likewise, many identified DAPs are related to the effects of neurotransmitter mechanism of action, i.e., Vesicle-mediated transport, signal transduction and second messengers, Rho-GTPase cycle, transport to the Golgi, extracellular matrix organization, calcium-dependent signal transduction, and synaptic vesicles. Although many of these proteins (small GTPases, for example) are involved in many pathways, they are also clearly involved in NT action. It has to be noted that the scarce proteomic studies of the effects of an HFD in the brain did not identify changes to proteins directly related to NTs, but proteins related to neuronal development, vesicle trafficking, cytoskeletal function and structure, signal transduction, synaptic vesicles, and other biological processes [61,62,63].

Finally, there have been reported sex-related differences in the effects of IUGR on productive parameters in young pigs [25,78], and in the effects of IUGR on the hippocampus proteome in fetuses [29]. However, the results of the present study indicate that these differences seem not to remain in the adult age.

## 5. Conclusions

An HFD provokes an increase in 5-HT in the five analyzed brain areas, and also in DA in the hypothalamus. Both, serotonin and DA have an anorexigenic function in the brain, probably contributing to the satiating effect of an HFD.

Our results indicate that an HFD does not provoke relevant changes in the morphology of the hippocampus. The observed effects in CA1 and DG (a mild increase in the percentage of the area occupied by neurons) were observed in NBW and LBW groups, that is, independently of the initial birth weight.

An HFD provokes differences in the proteome of the hippocampus. Some changes are common to both NBW and LBW groups as vesicle-mediated transport and some metabolic proteins, but other are specific to one of the groups, and then influenced by birth weight (IUGR). In particular, NBW animals present changes in the mitochondrial respiratory chain and oxidative phosphorylation, and in the extracellular matrix and its interaction with the cell. LBW animals present differences in RNA splicing, anterograde and retrograde transport, and the mTOR pathway.

Research on the relationship between IUGR and the late consequences of an obesogenic diet may benefit from the knowledge from large animal models as the Iberian pig.

## Figures and Tables

**Table 1:** Concentration of neurotransmitters and their metabolites (ng/g tissue) in different brain areas of 1 year-old pigs as a function of diet (Ctrl and HFD), their birth weight (NBW and LBW), and their interaction.

NT		NBW	LBW	*p*-Value		
				Diet	Weight	Interaction
Hippocampus						
DOPAC	CTRL	63.87 ± 4.59	74.40 ± 4.13	**0.024**	0.287	0.370
	HFD	81.10 ± 4.79	82.01 ± 6.62			
HVA	CTRL	87.12 ± 4.33	94.12 ± 5.20	0.836	0.998	0.182
	HFD	93.06 ± 4.47	86.04 ± 6.27			
DOPtotal	CTRL	145.90 ± 7.95	168.52 ± 8.08	0.253	0.264	0.150
	HFD	168.77 ± 6.30	165.85 ± 11.71			
5-HT	CTRL	336.03 ± 14.03	367.69 ± 13.88	**<0.001**	0.371	0.551
	HFD	434.76 ± 23.53	441.15 ± 25.38			
5-HIAA	CTRL	191.93 ± 8.13	213.85 ± 8.08	0.209	0.187	0.326
	HFD	213.22 ± 7.27	216.49 ± 13.56			
INDtotal	CTRL	533.40 ± 18.72	581.54 ± 19.35	**0.001**	0.331	0.543
	HFD	655.59 ± 31.81	666.77 ± 39.48			
Amygdala						
DA	CTRL	705.45 ± 33.80	682.81 ± 32.18	0.947	0.734	0.387
	HFD	665.36 ± 39.76	717.17 ± 60.81			
DOPAC	CTRL	447.20 ± 41.74	543.63 ± 51.70	0.828	0.150	0.447
	HFD	470.87 ± 36.03	501.09 ± 43.99			
HVA	CTRL	814.35 ± 19.52	861.67 ± 63.50	0.285	0.809	0.543
	HFD	908.06 ± 52.11	887.69 ± 71.62			
DOPtotal	CTRL	1966.99 ± 68.37	2088.12 ± 121.17	0.928	0.343	0.862
	HFD	1995.30 ± 90.14	2079.17 ± 143.26			
5-HT	CTRL	1501.57 ± 95.32	1567.02 ± 99.18	**0.012**	0.171	0.482
	HFD	1685.72 ± 80.52	1886.99 ± 107.41			
5-HIAA	CTRL	618.86 ± 27.66	678.11 ± 27.91	0.575	0.082	0.786
	HFD	643.03 ± 20.20	686.51 ± 38.39			
INDtotal	CTRL	2120.43 ± 100.44	2245.12 ± 102.41	**0.013**	0.061	0.452
	HFD	2315.06 ± 89.82	2601.64 ± 129.96			
Prefrontal cortex						
DOPAC	CTRL	71.95 ± 8.07	70.87 ± 5.75	0.818	0.910	0.945
	HFD	70.17 ± 4.05	69.91 ± 6.01			
HVA	CTRL	150.58 ± 5.95	158.25 ± 6.52	0.307	0.697	0.386
	HFD	149.63 ± 5.82	146.70 ± 5.50			
DOPtotal	CTRL	222.53 ± 7.88	229.12 ± 9.81	0.361	0.838	0.557
	HFD	219.80 ± 8.40	216.60 ± 6.40			
5-HT	CTRL	325.93 ± 13.14	362.59 ± 26.87	**0.046**	0.189	0.740
	HFD	378.39 ± 17.81	400.40 ± 26.18			
5-HIAA	CTRL	163.75 ± 9.58	170.88 ± 9.90	0.385	0.326	0.884
	HFD	169.89 ± 3.98	179.48 ± 9.77			
INDtotal	CTRL	489.68 ± 21.47	533.47 ± 34.18	0.073	0.193	0.832
	HFD	548.29 ± 20.52	579.88 ± 34.62			
Hypothalamus						
DA	CTRL	361.95 ± 30.94	348.53 ± 27.16	**0.039**	0.569	0.905
	HFD	428.97 ± 20.97	408.44 ± 31.99			
L-DOPA	CTRL	652.69 ± 54.48	600.27 ± 104.22	0.13	0.212	0.653
	HFD	781.39 ± 51.58	670.69 ± 55.58			
DOPAC	CTRL	511.69 ± 28.01	585.81 ± 60.98	0.817	0.813	0.163
	HFD	564.78 ± 51.55	511.92 ± 24.00			
HVA	CTRL	616.73 ± 57.76	606.47 ± 50.58	0.876	0.715	0.876
	HFD	616.73 ± 39.71	591.17 ± 38.61			
DOPtotal	CTRL	2225.07 ± 143.97	2057.62 ± 255.38	0.293	0.176	0.878
	HFD	2391.87 ± 91.40	2182.21 ± 98.13			
5-HT	CTRL	1250.61 ± 78.97	1146.07 ± 151.71	**0.023**	0.732	0.180
	HFD	1352.55 ± 88.09	1527.81 ± 97.94			
5-HIAA	CTRL	645.40 ± 37.09	622.28 ± 48.06	0.297	0.943	0.601
	HFD	665.78 ± 35.64	683.32 ± 29.96			
INDtotal	CTRL	1907.75 ± 100.41	1768.35 ± 162.49	**0.033**	0.832	0.191
	HFD	2018.33 ± 110.94	2211.14 ± 120.42			
Striatum						
DA	CTRL	21,834.51 ± 2607.80	25,637.38 ± 2479.18	0.373	**0.007**	0.280
	HFD	21,412.39 ± 1348.85	29,970.72 ± 2353.49			
L-DOPA	CTRL	2275.64 ± 248.13	1962.68 ± 242.11	0.528	0.061	0.636
	HFD	2516.53 ± 221.93	1997.44 ± 122.63			
DOPAC	CTRL	4260.22 ± 217.81	4320.28 ± 189.50	**0.011**	0.437	0.653
	HFD	3693.90 ± 116.83	3918.48 ± 201.54			
HVA	CTRL	11,262.32 ± 795.05	12,643.72 ± 689.32	0.175	0.113	0.655
	HFD	10,641.91 ± 602.61	11,422.03 ± 578.05			
3-MT	CTRL	1210.46 ± 96.21	1472.53 ± 56.29	0.151	**0.046**	0.272
	HFD	1181.48 ± 71.50	1259.28 ± 89.19			
DOPtotal	CTRL	38,533.33 ± 1575.54	46,228.27 ± 2016.19	0.721	**0.002**	0.401
	HFD	39,446.20 ± 1443.33	43,976.56 ± 2328.97			
5-HT	CTRL	705.05 ± 29.61	720.23 ± 23.70	**0.024**	0.119	0.309
	HFD	740.54 ± 24.01	811.32 ± 29.54			
5-HIAA	CTRL	533.18 ± 33.11	540.28 ± 29.01	0.230	0.459	0.629
	HFD	553.12 ± 23.37	586.66 ± 22.67			
INDtotal	CTRL	1270.54 ± 50.59	1260.51 ± 45.79	0.057	0.239	0.161
	HFD	1293.66 ± 31.07	1406.73 ± 46.71			

Concentrations are presented as the mean ± SE. The columns show the neurotransmitters and metabolites in function of birth weight (LBW or NBW). The rows divide pigs in function of diet (Ctrl of HFD). *p*-values in bold indicate significant differences. DA: Dopamine; DOPAC: 3,4-dihydroxyphenyl acetic acid; HVA: Homovanillic acid; 3-MT: 3-Methoxytyramine; 5-HT: Serotonin/5-Hydroxytryptamine; 5-HIAA: 5-Hydroxyindoleacetic acid; DOP total: Total dopaminergic neurotransmitters; IND total: Total serotoninergic neurotransmitters. Statistical significance was determined by UNIANOVA with Tukey adjustment.

**Table 2:** Effect of HFD on NeuN immunostaining in the hippocampus of one year-old pigs born with normal birth weight (NBW) or low birth weight as result of IUGR (LBW) and fed a control diet (Ctrl) or an HFD diet.

			NBW	LBW	*p*-Value		
					Diet	Weight	Interaction
**CA1**	**Number of neurons**	**CTRL**	**352.81 ± 17.18**	481.00 ± 161.83	0.897	**<0.001**	0.539
		**HFD**	336.84 ± 12.63	505.50 ± 22.00			
	**Mean size (µm^2^)**	**CTRL**	650.90 ± 52.89	626.75 ± 145.43	0.521	0.175	0.309
		**HFD**	767.67 ± 30.49	600.20 ± 34.96			
	**Area** **(%)**	**CTRL**	12.60 ± 0.61	15.00 ± 1.94	**0.037**	**0.010**	0.759
		**HFD**	14.45 ± 0.46	17.47 ± 0.98			
**DG**	**Number of neurons**	**CTRL**	208.56 ± 10.39	217.33 ± 15.34	0.084	0.333	0.825
		**HFD**	226.42 ± 4.74	240.38 ± 5.55			
	**Mean size (µm^2^)**	**CTRL**	1345.26 ± 45.64	1131.78 ± 164.07	0.575	0.050	0.260
		**HFD**	1307.23 ± 29.95	1244.58 ± 26.85			
	**Area** **(%)**	**CTRL**	15.92 ± 0.55	13.96 ± 1.20	**0.010**	0.322	0.180
		**HFD**	17.02 ± 0.39	17.32 ± 0.48			
	**Width** **(µm)**	**CTRL**	92.44 ± 2.36	92.72 ± 3.29	**0.008**	0.781	0.857
		**HFD**	84.08 ± 1.75	85.39 ± 4.57			

Results are presented as (mean ± SE). In the main effects, significant differences are marked in bold. ImageJ was used for quantification.

**Table 3:** Response of NBW and LBW animals to an HFD. Comparison between GO categories in NBW and LBW groups: Main categories from Panther have been selected and fold of enrichment indicated. Only main categories with the highest enrichment fold are shown.

NBW	Enrichment	LBW	Enrichment
Biological Process			
negative regulation of synaptic vesicle exocytosis (GO:2000301)	69.81	negative regulation of mRNA splicing, via spliceosome (GO:0048025)	24.25
oxidative phosphorylation (GO:0006119)	7.21	regulation of alternative mRNA splicing, via spliceosome (GO:0000381)	16.01
ATP metabolic process (GO:0046034)	5.18	post-Golgi vesicle-mediated transport (GO:0006892)	9.90
regulation of vesicle-mediated transport (GO:0060627)	3.96		
Molecular Function			
cadherin binding (GO:0045296)	3.96	pre-mRNA binding (GO:0036002)	16.42
cytoskeletal protein binding (GO:0008092)	2.90	structural constituent of cytoskeleton (GO:0005200)	7.18
oxidoreductase activity (GO:0016491)	2.82	cadherin binding (GO:0045296)	4.96
RNA binding (GO:0003723)	2.07	actin binding (GO:0003779)	4.48
Cellular Component			
platelet alpha granule membrane (GO:0031092)	20.53	UFD1-NPL4 complex (GO:0036501)	>100
cytochrome complex (GO:0070069)	11.35	endoplasmic reticulum-Golgi intermediate compartment membrane (GO:0033116)	6.93
lysosomal lumen (GO:0043202)	7.20	ruffle membrane (GO:0032587)	6.47
basement membrane (GO:0005604)	7.20	exocytic vesicle (GO:0070382)	4.66
lipid droplet (GO:0005811)	7.20	postsynaptic density (GO:0014069)	4.46
Schaffer collateral-CA1 synapse (GO:0098685)	7.01	asymmetric synapse (GO:0032279)	4.39
respiratory chain complex (GO:0098803)	6.54	postsynaptic specialization (GO:0099572)	4.14
glutamatergic synapse (GO:0098978)	5.32		
secretory granule membrane (GO:0030667)	3.74		
secretory granule lumen (GO:0034774)	3.62		

**Table 4:** List of the most representative differential proteins involved in the response of NBW and LBW pigs to an HFD identified by KEGG pathway analysis.

	Pathways	n	Proteins
NBW	Metabolic pathways	25	COQ7; ATP5PD; CKMT2; COX6B1; COX7A2; COX15; STT3B; AK2; ALDH1A3; PLCH1; GLUD2; GNS; GSTP1; APRT; ARSA; ND1; MVD; NDUFS5; ACOX1; PKM; UQCRC2; CA4; DGKE; AGPS; KYAT1
	Thermogenesis	10	ATP5PD; COX6B1; COX7A2; COX15; ND1; NDUFS5; COA1; MAP2K3; RHEB; UQCRC2
	Oxidative phosphorylation	7	ATP5PD; COX6B1; COX7A2; COX15; ND1; NDUFS5; UQCRC2
	Autophagy	7	AKT2; ATG4B; ITPR1; LAMP2; ZFYVE1; RHEB; ATG3
	Focal adhesion and ECM–receptor interaction	7	COL4A1; AKT2; PARVB; ITGA7; PPP1CA; RAP1B; BRAF; HSPG2; AGRIN
	Insulin signaling pathway	6	AKT2; PPP1CA; PRKAR1B; PTPRF; RHEB; BRAF
	cAMP signaling pathway	6	AKT2; ATP2A1; ACOX1; PPP1CA; RAP1B; BRAF
	Lysosome	5	NPC2; GNS; LAMP2; ARSA; AP3B1
	MAPK signaling pathway	5	AKT2; NF1; MAP2K3; RAP1B; BRAF
	Protein processing in the ER	4	SEC63; STT3B; NPLOC4; PDIA4
LBW	Metabolic pathways	30	CBS; CDIPT; PAICS; DGKB; FASN; PLCH1; SIRT5; TKFC; GCSH; GFPT1; ACAD8; MAT2B; GMPPA; HADHA; ARSB; ND1; NDUFS5; ALDH7A1; PDE4B; COQ3; PIK3C3; PMM1; CYCS; UCKL1; PPT1; ECHDC1; CHPT1; CBR1; KYAT1; PIGS
	Thermogenesis	7	ND1; NDUFS5; COA1; RPTOR; RHEB; RPS6KA3; SMARCC1
	Spliceosome	7	U2AF2; DDX5; HNRNPK; SRSF4; SRSF6; TRA2B; RBM8A
	Autophagy	6	PIK3R4; PIK3C3; MAPK10; RPTOR; RHEB; DEPTOR
	Protein processing in endoplasmic reticulum	6	SEC61B; DNAJB2; NPLOC4; MAPK10; SEC62; UFD1
	cAMP signaling pathway	5	GRIA4; AFDN; ATP2A1; PDE4B; MAPK10
	Lysosome	5	AP3S1; FUCA1; ARSB; PPT1; SORT1
	mTOR signaling pathway	4	RPTOR; RHEB; RPS6KA3; DEPTOR
	Focal adhesion	4	DIAPH1; ITGB8; PARVA; MAPK10
	Insulin signaling pathway	4	FASN; MAPK10; RPTOR; RHEB

**Table 5:** Differentially abundant proteins identified in NBW and LBW animals subjected to Ctrl and HFD diets. In red: Upregulated in HFD versus Ctrl. In Green: Down-Regulated in HFD versus Ctrl.

	Access UniProt	Gene	Identification
NBW	Oxidative phosphorylation/Respiratory chain		
	F1RPD2	*UQCRC2*	Cytochrome b-c1 complex subunit 2, mitochondrial
	F1S8W1	*COX15*	Cytochrome c oxidase assembly protein COX15 homolog isoform 1
	F1S4V0	*COX7A2*	Cytochrome c oxidase subunit 7A2, mitochondrial
	I3LR62	*COA1*	Cytochrome c oxidase assembly factor 1 homolog
	F1SV23	*NDUFS5*	Complex I-15 kDa
	O79874	*NADH1*	NADH-ubiquinone oxidoreductase chain 1
	A0A5G2QL31	*COX6B1*	Cytochrome c oxidase subunit 6B1
	F1SMF9	*ATP5PD*	ATP synthase subunit d, mitochondrial
	Focal adhesion and ECM–receptor interaction		
	I3LGD9	*AGRN*	Agrin
	F1SJU4	*PARVB*	Parvin beta
	A0A481B0D0	*ITGA7*	Integrin alpha-2
	M3V819	*COL4A1*	Collagen alpha-1 (IV) chain isoform 1 preproprotein
	F1RW32	*SPARCL1*	SPARC like 1
	F1SU03	*HSPG2*	Heparan sulfate proteoglycan 2
LBW	mRNA splicing		
	I3LFJ5	*SUGP2*	SURP and G-patch domain containing 2
	F1S6R7	*PTBP1*	Polypyrimidine tract-binding protein 1
	F1RZV6	*KHDRBS2*	KH RNA binding domain containing, signal transduction associated 2
	P80230	*ERH*	Enhancer of rudimentary homolog
	Q06AA7	*TRA2B*	Transformer 2 beta homolog
	I3LDY1	*EML1*	Echinoderm microtubule-associated protein-like 1
	F6QB00	*SRSF4*	Serine/arginine-rich splicing factor 4 isoform X1
	I3W8V7	*U2AF2*	Splicing factor U2AF 65 kDa subunit
	I3LI59	*RBM8A*	RNA-binding protein 8A
	A0A286ZM27	*DDX5*	DEAD box protein 5
	I3LQS0	*HNRNPK*	Heterogeneous nuclear ribonucleoprotein K
	K7GNF4	*FMR1*	Synaptic functional regulator FMR1
	F1RUN0	*CELF2*	CUGBP Elav-like family member 2
	A0A4X1TTZ9	*SRSF6*	Serine/arginine-rich splicing factor 6
	K7GNY3	*KHDRBS3*	KH domain-containing, RNA-binding, signal transduction-associated protein 3
	mTOR signaling pathway		
	Q5D891	*PIK3C3*	Phosphatidylinositol 3-kinase catalytic subunit type 3
	K9IWD2	*PIK3R4*	Phosphoinositide 3-kinase regulatory subunit 4
	A0A480DP04	*DEPTOR*	DEP domain-containing mTOR-interacting protein
	I3L942	*RPTOR*	Regulatory associated protein of MTOR complex 1
	F1SQN4	*RPS6KA3*	Ribosomal protein S6 kinase alpha-3
	F2Z5R2	*RHEB*	GTP-binding protein Rheb

## Data Availability

The MS proteomics data has been uploaded to the ProteomeXchange Consortium via the PRIDE partner repository with the dataset identifier PXD032300.

## Supplementary Materials

The following supporting information can be downloaded at: https://www.mdpi.com/article/10.3390/nu14163440/s1, Figure S1: Complete Voronoi diagram obtained with Reactome showing the main pathways regulated by an HFD in NBW and LBW groups; Figure S2: Network analysis by STRING of differentially abundant proteins in the hippocampus of one year-old pigs after being subjected to an HFD; Table S1: Design of the TMT experiment; Table S2: Effect of diet, birth weight and sex on the concentration of neurotransmitters in different brain areas; Table S3: Differentially abundant proteins in the hippocampus by diet, birth weight and sex; Table S4: List of differentially abundant proteins in the hippocampus comparing NBW-Ctrl versus NBW-HFD and LBW-Ctrl versus LBW-HFD; Table S5: Gene Ontology analysis of the response of NBW and LBW groups to an HFD; Table S6: KEGG pathway analysis of the response of NBW and LBW groups to an HFD; Table S7: List of common and different proteins in NBW and LBW groups in response to an HFD.
