# Supplementary material for: A High-Fat Diet Modifies Brain Neurotransmitter Profile and Hippocampal Proteome and Morphology in an IUGR Pig Model
Source: Nutrients. 2022 Aug 22;14(16):3440. doi: 10.3390/nu14163440 (PMC9416793; doi:10.3390/nu14163440)
Supplement: Supplementary file 1 [file nutrients-14-03440-s001.zip › nutrients-1856500-supplementary.pdf]

Supplementary Materials

Figure S1. Complete Voronoi diagram obtained with Reactome showing the main pathways regulated by an HFD in NBW and LBW groups.

Complete Voronoi figure and individual images shown in Figure 6 with enlarged letter size:

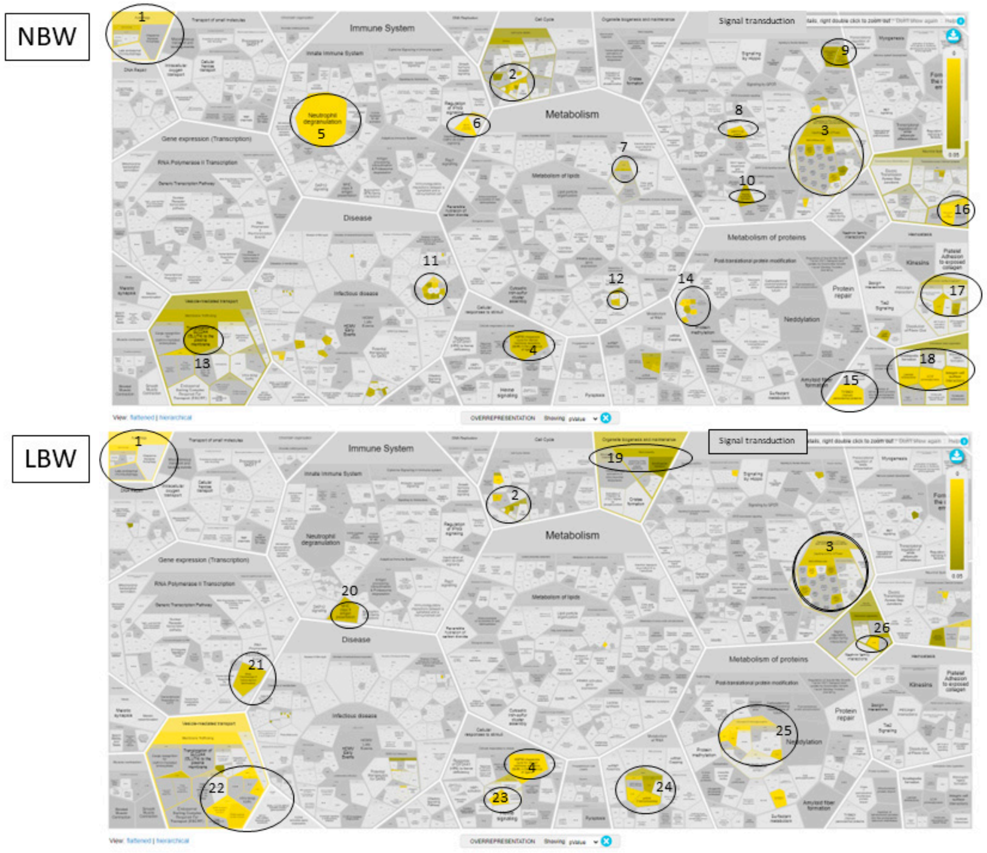

Supplementary Figure S1. Voronoi diagram obtained with Reactome showing the main pathways regulated by HFD in NBW and LBW groups.

Common regulated pathways to both NBW and LBW groups:

1. Apoptosis
2. Cell cycle
3. Signaling by Rho GTPases
4. HSP90

Overabundant in NBW:

5. Neutrophil degranulation
6. Antiviral mechanism
7. Mitochondrial respiratory chain. ATP synthesis
8. DARPP-32 events
9. Linkage to MAPK to integrins
10. MAPK activation
11. MAPK signaling
12. Glucose metabolism
13. Translocation of SLC2A4 (GLUT4)
14. Sumoylation
15. Peroxisome
16. Neurexins
17. Platelet degranulation
18. Laminin, ECM Proteoglycans, Integrin interaction

Overabundant in LBW:

19. Organelle biogenesis and maintenance
20. Antigen presentation
21. Transcription termination
22. Vesicle-mediated transport: Anterograde and retrograde transport
23. Activation of chaperone genes
24. Splicing
25. Anterograde transport, transport to the Golgi and N-glycosylation
26. Cell communication

Signalling by Rho GTPases

NBW

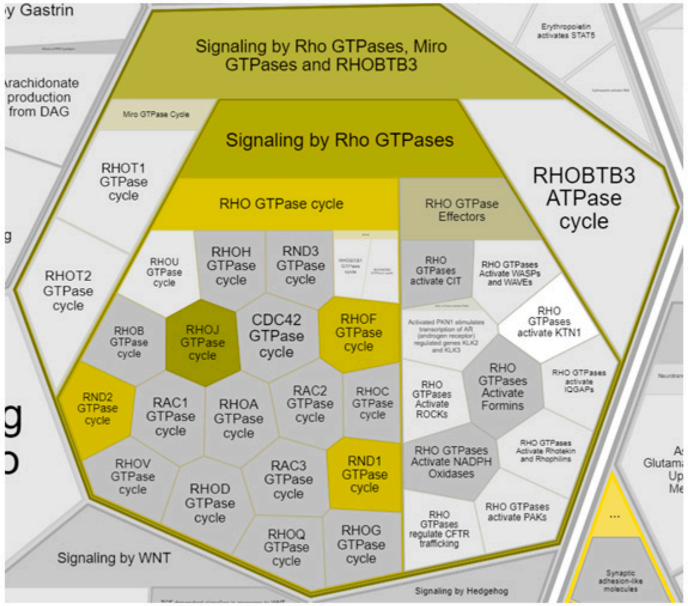

LBW

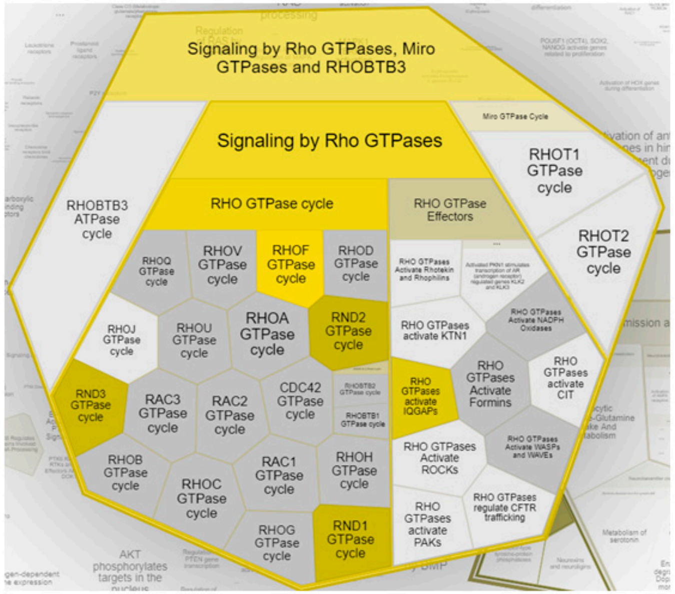

Note: shapes are not exactly the same than in slide #1 since Reactome has been run at different times

Vesicle-mediated transport

NBW

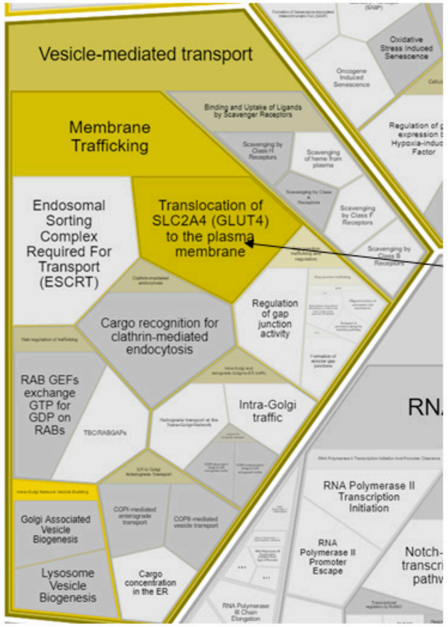

Translocation of SLC2A4 (GLUT4)

LBW

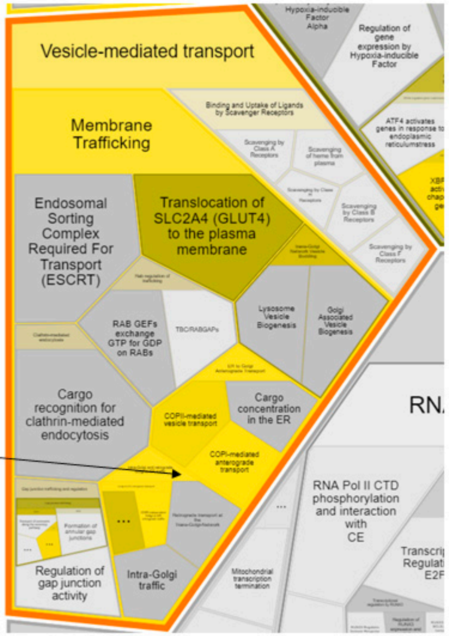

Vesicle-mediated transport: Anterograde and retrograde transport

Note: shapes are not exactly the same than in slide #1 since Reactome has been run at different times

Anterograde transport, transport to the Golgi and N-glycosylation

NBW

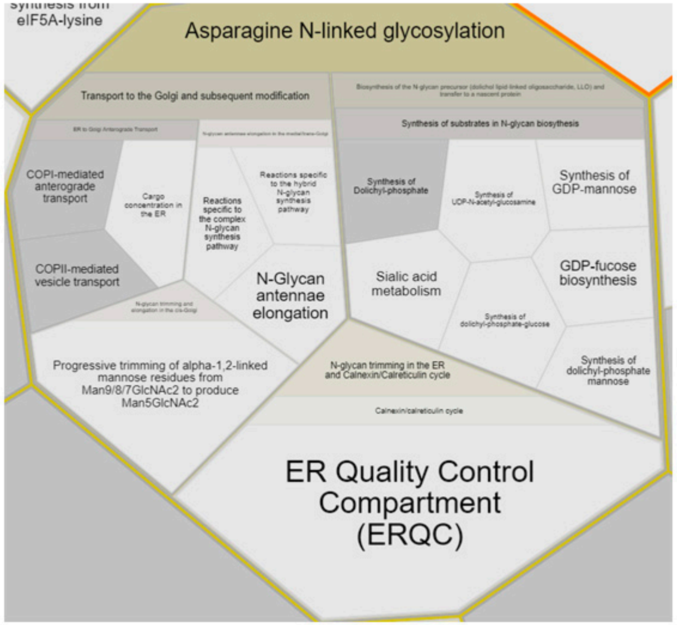

LBW

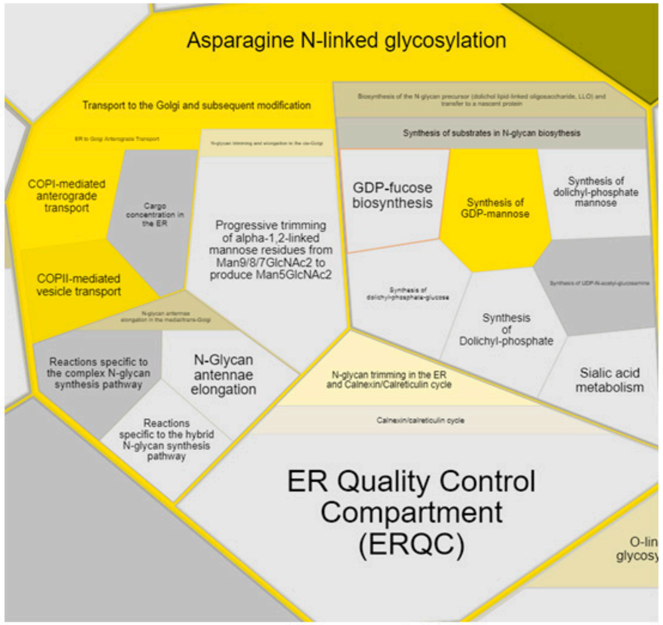

Note: shapes are not exactly the same than in slide #1 since Reactome has been run at different times

Mitochondrial respiratory chain
ATP synthesis

NBW

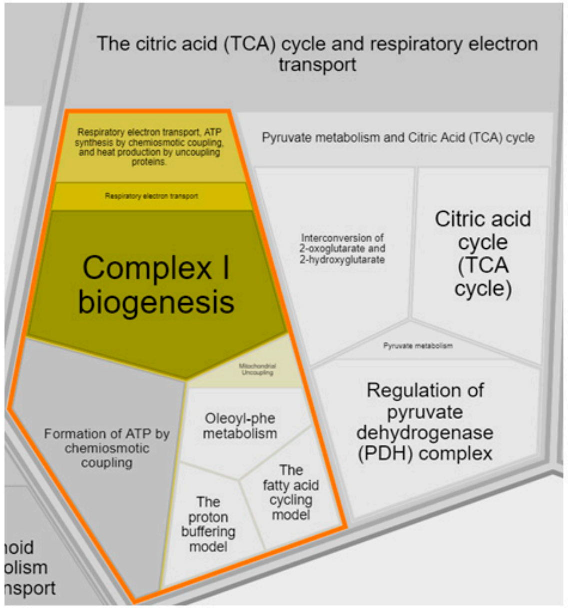

LBW

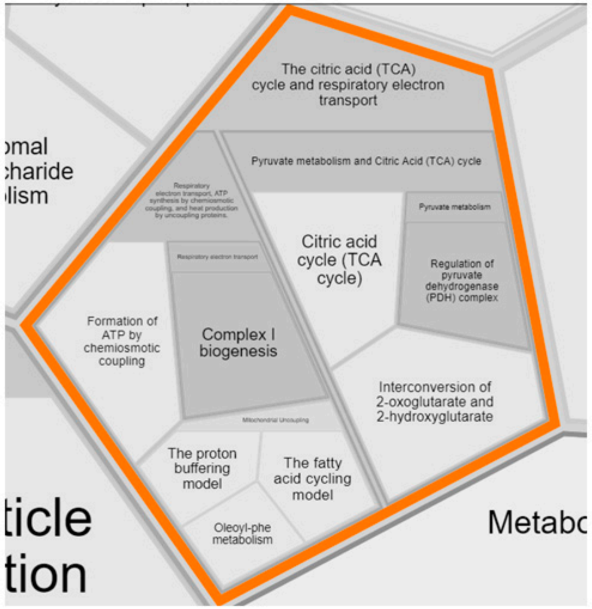

Note: shapes are not exactly the same than in slide #1 since Reactome has been run at different times

Laminin, ECM Proteoglycans,
Integrin interaction

NBW

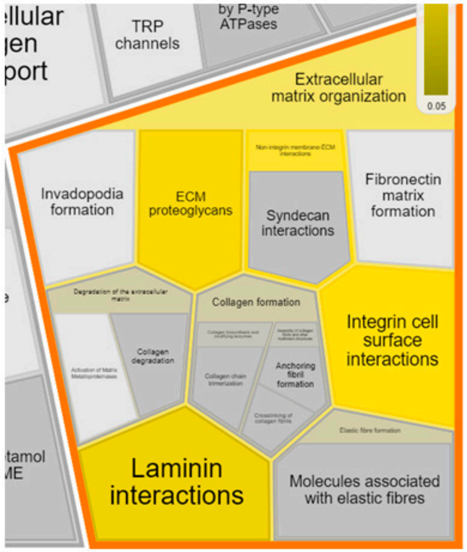

LBW

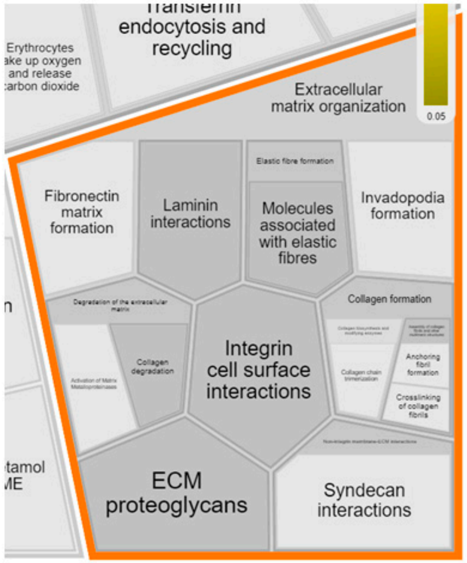

Note: shapes are not exactly the same than in slide #1 since Reactome has been run at different times

RNA splicing

NBW

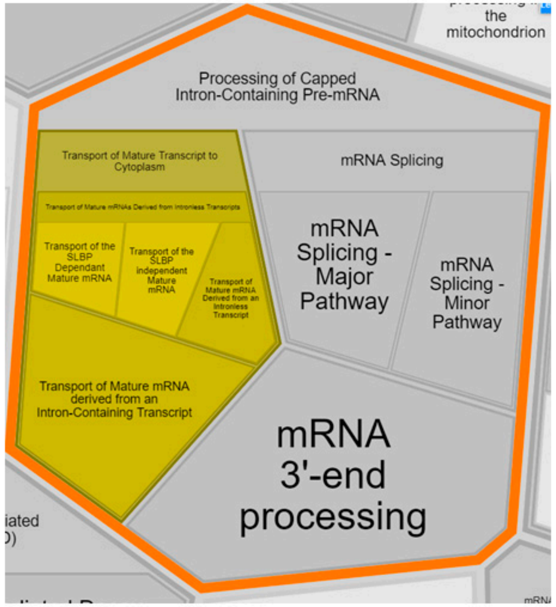

LBW

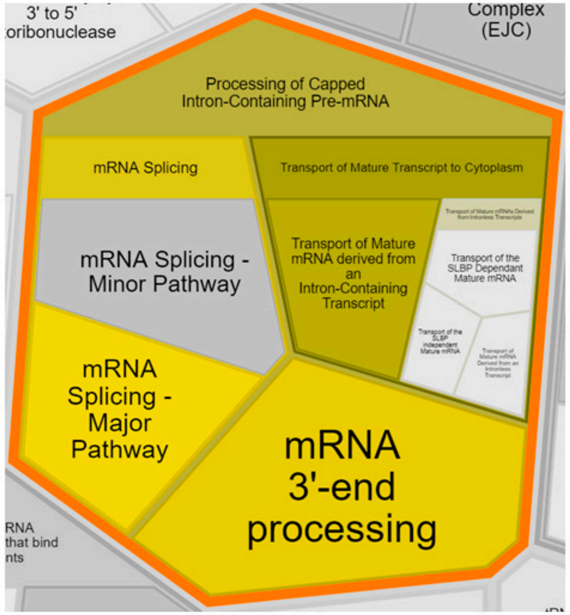

Note: shapes are not exactly the same than in slide #1 since Reactome has been run at different times

Figure S2. Network analysis by STRING of differentially abundant proteins in the hippocampus of one year-old pigs after being subjected to an HFD.

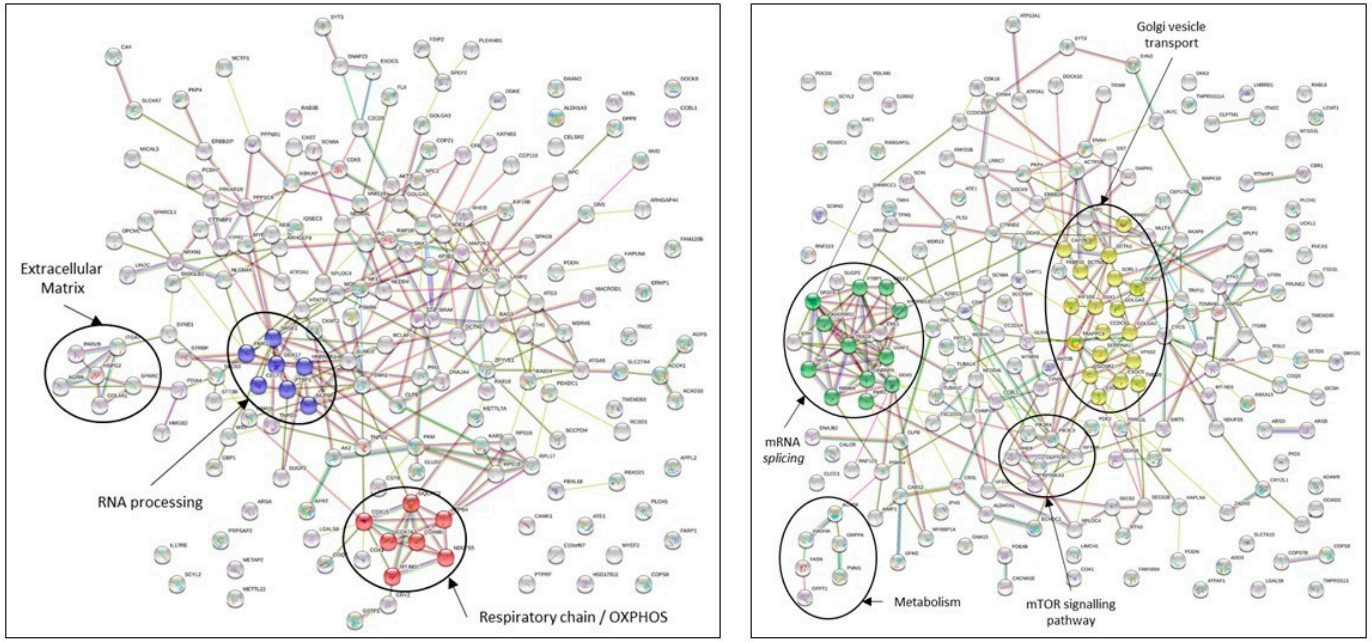

Supplementary Figure S2. Network analysis by STRING of differentially abundant proteins in the hippocampus of one year-old pigs after being subjected to an HFD. Upper panel: NBW group. Lower panel: LBW group. Different colours of the lines represent the types of evidence for association: Cyan line: database; Pink line: experimental; Green line: gene neighborhood evidence; Yellow line, text mining evidence; Black line, co-expression evidence.

Table S1. Design of the TMT experiment.

Experimental design for TMT10 labelling for individual samples from groups with different diet (Ctrl, HFD), birthweight (NBW, LBW) or sex (M, F). Pools are formed by mixing all samples

Batch	1	2	3
126	Pool 1a	Pool 2a	Pool 3a
127N	C1.1	C2.2	T2.3
127C	T1.1	T4.2	T3.3
128N	C2.1	C1.2	C3.3
128C	T2.1	C4.2	C1.3
129N	C3.1	T1.2	C2.3
129C	T3.1	T2.2	T1.3
130N	C4.1	T3.2	T4.3
130C	T4.1	C3.2	C4.3
131	Pool 1b	Pool 2b	Pool 3b

Ctrl, NBW, M	HFD, NBW, M
Ctrl, NBW, F	HFD, LBW, F
Ctrl, LBW, M	HFD, LBW, M
Ctrl, LBW, F	HFD, LBW, F

Table S2. Effect of diet, birth weight and sex on the concentration of neurotransmitters in different brain areas.

Concentration of neurotransmitters and their metabolites in several brain areas of 1 year-old pigs as a function of diet (Ctrl or HFD), birthweight (NBW or LBW) and sex (M, F). Significant differences are indicated in bold ($p < 0.05$).

		Diet		Birthweight		Sex	
		CTRL	HFD	NBW	LBW	Males	Females
Amygdala	NA	354.70 ± 36.20	354.60 ± 25.91	316.88 ± 20.52	394.20 ± 36.02	372.09 ± 39.03	339.47 ± 20.54
	**DA**	695.26 ± 23.10	687.56 ± 34.11	684.53 ± 26.06	699.99 ± 33.63	677.19 ± 29.73	706.16 ± 28.67
	**DOPAC**	490.59 ± 33.64	485.38 ± 27.80	460.02 ± 26.83	519.33 ± 32.99	464.62 ± 26.86	509.77 ± 32.60
	**HVA**	835.64 ± 30.06	898.72 ± 42.35	865.11 ± 30.63	875.98 ± 47.51	828.29 ± 31.26	908.18 ± 42.09
	**5-HT**	**1532.74 ± 67.39**	**1781.98 ± 68.26**	1597.65 ± 63.65	1734.62 ± 80.04	1621.62 ± 85.47	1708.37 ± 52.73
	**5-HIAA**	647.07 ± 20.28	662.96 ± 20.72	631.95 ± 16.56	682.51 ± 23.53	666.60 ± 21.95	643.98 ± 18.78
	**Glutamate**	1378.05 ± 29.57	1379.02 ± 22.41	1365.29 ± 25.09	1393.17 ± 25.51	1366.06 ± 26.43	1391.14 ± 24.21
	**GABA**	234.41 ± 14.90	228.83 ± 9.40	220.78 ± 10.35	242.58 ± 13.39	223.87 ± 15.25	239.35 ± 6.90
PFC	**NA**	140.40 ± 12.82	127.30 ± 5.21	137.49 ± 10.18	128.02 ± 7.18	138.50 ± 11.46	127.41 ± 5.12
	**DOPAC**	71.41 ± 4.81	70.04 ± 3.50	70.90 ± 3.97	70.32 ± 4.13	73.99 ± 4.87	67.40 ± 2.95
	**HVA**	154.42 ± 4.38	148.22 ± 3.94	150.02 ± 4.12	151.65 ± 4.29	157.53 ± 4.20	144.40 ± 3.70
	**5-HT**	**343.30 ± 14.70**	**388.96 ± 15.44**	355.58 ± 12.62	384.19 ± 18.89	383.08 ± 18.84	355.39 ± 12.03
	**5-HIAA**	167.13 ± 6.75	174.50 ± 5.11	167.22 ± 4.65	175.80 ± 6.91	179.09 ± 6.64	163.54 ± 4.36
	**Glutamate**	1395.06 ± 53.18	1363.04 ± 50.82	1372.83 ± 51.47	1380.70 ± 53.19	**1451.83 ± 47.17**	**1294.20 ± 51.64**
	**GABA**	158.84 ± 6.88	149.67 ± 6.45	150.67 ± 6.07	156.48 ± 7.39	159.16 ± 7.31	147.59 ± 5.81

Hippocampus	**NA**	146.01 ± 8.33	157.59 ± 7.46	149.08 ± 7.68	155.20 ± 8.16	154.97 ± 8.47	149.81 ± 7.48
	**DOPAC**	**69.14 ± 3.24**	**81.54 ± 3.95**	73.61 ± 3.76	78.55 ± 4.07	75.57 ± 3.97	76.47 ± 3.93
	**HVA**	90.62 ± 3.39	89.84 ± 3.75	90.48 ± 3.15	89.89 ± 4.11	87.93 ± 3.97	92.47 ± 3.16
	**5-HT**	**351.86 ± 10.27**	**437.69 ± 16.89**	391.84 ± 17.74	406.17 ± 16.65	413.31 ± 19.18	384.04 ± 14.60
	**5-HIAA**	202.37 ± 6.11	214.71 ± 7.14	203.04 ± 5.77	215.17 ± 7.69	210.31 ± 7.58	207.12 ± 6.00
	**Glutamate**	1564.26 ± 43.09	1542.23 ± 31.59	1528.05 ± 37.84	1579.79 ± 35.74	**1623.69 ± 31.45**	**1487.96 ± 36.46**
	**GABA**	324.92 ± 19.73	326.24 ± 14.97	317.92 ± 14.38	334.10 ± 19.76	334.22 ± 14.96	316.25 ± 19.20
Striatum	**NA**	369.41 ± 13.79	361.72 ± 10.25	362.73 ± 10.87	367.65 ± 12.83	**392.53 ± 6.65**	**336.5 ± 13.02**
	**DA**	23735.94 ± 1804.63	25133.4 ± 1537.62	**21595.92 ± 1331.36**	**27804.05 ± 1736.27**	24779.68 ± 1400.72	24173.06 ± 1917.56
	**L-DOPA**	2126.61 ± 172.94	2278.61 ± 140.8	2406.12 ± 163.75	1980.89 ± 128.51	2300.49 ± 156.33	2110.65 ± 154.25
	**DOPAC**	**4290.25 ± 140.67**	**3801.7 ± 114.06**	3940.13 ± 127.39	4101.12 ± 143.2	4117.79 ± 149.51	3915.37 ± 115.9
	**HVA**	11920.13 ± 539.6	11016.37 ± 417.44	10926.26 ± 482.43	11977.35 ± 453.49	11228.69 ± 519.96	11629.22 ± 439
	**3-MT**	1328.39 ± 64.61	1218.82 ± 56.06	**1194.76 ± 57.43**	**1350.67 ± 60**	1306.3 ± 58.02	1226.98 ± 62.96
	**5-HT**	**712.28 ± 18.78**	**772.98 ± 19.77**	724.27 ± 18.74	767.94 ± 21.27	757.9 ± 22.62	730.81 ± 17.29
	**5-HIAA**	536.56 ± 21.63	568.49 ± 16.4	543.98 ± 19.42	564.57 ± 18.49	574 ± 18.65	532.25 ± 18.66
	**Glutamate**	1306.26 ± 19.69	1331.95 ± 29.96	1340.03 ± 26.51	1295.69 ± 23.4	1348.97 ± 23.78	1288.43 ± 26.01
	**GABA**	350.4 ± 10.1	366.95 ± 10.51	362.93 ± 9.29	355.6 ± 11.8	370.31 ± 10.13	348.55 ± 10.41
Hypotha-	**NA**	8297,63 ± 703,64	10690,63 ± 779,06	9691,9 ± 781,6	9881,75 ± 896,83	9130,45 ± 611,61	10420,87 ± 981,88
	**DA**	**356,92 ± 21,31**	**419,56 ± 18,24**	399,83 ± 18,87	387,29 ± 23,37	394,44 ± 20,02	394,56 ± 21,45

**L-DOPA**	631,72 ± 51,15	730,65 ± 38,73	728,74 ± 39,39	645,83 ± 50,18	696,4 ± 47,47	689,34 ± 43,03
**DOPAC**	539,48 ± 29,07	540,55 ± 29,93	541,69 ± 31,47	538 ± 26,85	527,82 ± 22,62	551,25 ± 35,08
**HVA**	612,88 ± 39,66	605,02 ± 27,38	616,73 ± 32,86	596,57 ± 29,81	**555,2 ± 28,34**	**656,08 ± 31,46**
**5-HT**	**1213,71 ± 72,23**	**1432,88 ± 66,6**	1305,82 ± 59,57	1393,08 ± 92,18	1299,07 ± 69,12	1382,89 ± 76,5
**5-HIAA**	636,15 ± 28,47	673,82 ± 23,26	657,45 ± 25,46	661,78 ± 25,9	646,87 ± 27,13	671,18 ± 24,31
**Glutamate**	875,05 ± 32,23	824,57 ± 26,24	873,1 ± 25,57	805,17 ± 31,94	**798,56 ± 19,47**	**890,43 ± 33,33**
**GABA**	384,69 ± 24,39	370,22 ± 11,92	381 ± 15,8	368,06 ± 17,28	385,36 ± 16,83	365,74 ± 16,04

Neurotransmitter concentrations are presented as mean ± SE. Units are in ng/g tissue except for glutamate and GABA, which are in μg/g tissue. NA: noradrenalin; DA: dopamine; DOPAC: 3,4-dihydroxyphenyl acetic acid; HVA: homovanillic acid; 3-MT: 3-Methoxytyramine; 5-HT: serotonin/5-hydroxytryptamine; 5-HIAA: 5-hydroxyindoleacetic acid; GABA: Gamma-Aminobutyric acid.

Table S3. Differentially abundant proteins in the hippocampus by diet, birth weight and sex.

Supplementary Table S3A: List of differential proteins identified comparing males and females

Accession Number	Access UniProt Human	Access Uni-Prot Pig	Gene	Identification	FC	p-value	M vs F
A0A4X1TU79	P22234	F1RTV5	PAICS	AIR carboxylase	0,61	0,001	down
A0A4X1SNG7	Q567U6	F1RY11	CCDC93	Coiled-coil domain-containing protein 93	0,61	0,018	down
I3LLA0	P49756	I3LLA0	RBM25	RNA-binding protein 25	0,65	0,015	down
A0A480JMC0	P47897	M3TYW5	QARS1	Glutaminyl-tRNA synthetase	0,66	0,023	down
A0A287AEY8	Q9Y3D7	F1RK50	PAM16	Mitochondrial import inner membrane translocase sub-unit TIM16	1,50	0,016	up
A0A5G2QU93	Q9GZY4	I3LR62	COA1	Cytochrome c oxidase assembly factor 1 homolog	1,52	0,018	up
M3VH45	P08133	F1S0V3	ANXA6	Annexin A6	1,53	0,024	up
A0A287B0D3	Q05586	A0A287B0D3	GRIN1	Glutamate receptor ionotropic, NMDA 1	1,54	0,047	up
A0A287AN84	Q9NRG1	I3LDD5	PRTFDC1	Phosphoribosyl transferase domain containing 1	1,70	0,008	up
A0A4X1VBH3	P53671	F2Z4Y3	LIMK2	LIM domain kinase 2	2,61	0,002	up

Supplementary Table S3B: List of differential proteins identified comparing birthweight

Accession	Accesion UniProt Human	Accession UniProt Pig	Gene	Identification	FC	p-va-lue	NBW vs LBW
A0A287AH65	Q15052	A0A287AH65	ARHGEF6	Rac/Cdc42 guanine nucleotide exchange factor 6	0,40	0,009	down
A0A480EJB5	Q3LXA3	F1RKQ4	TKFC	ATP-dependent dihydroxyacetone kinase	0,44	0,033	down
A0A480MD89	Q8WXH2	F1S6K2	JPH3	Junctophilin	0,59	0,045	down
A0A4X1UGZ2	P53004	F1SSF5	BLVRA	Biliverdin reductase A	0,62	0,009	down
A0A480X9B8	O94952	F1RKE9	FBXO21	F-box only protein 21	0,63	0,044	down
A0A4X1THN7	Q6NUK1	F6Q4L6	SLC25A24	Calcium-binding mitochondrial carrier protein SCaMC-1	0,64	0,001	down
E3US74	Q9P2P5	E3US74	HECW2	HECT-type E3 ubiquitin transferase	0,66	0,032	down

A0A4X1VAS0	Q9P2S2	A0A5G2R504	NRXN2	Neurexin-2	0,67	0,010	down
A0A5G2QEW3	P51812	F1SQN4	RPS6KA3	Non-specific serine/threonine protein kinase	1,51	0,026	up
A0A4X1TXB7	Q96KQ4	F1S9X3	PPP1R13B	Protein phosphatase 1 regulatory subunit 13B	1,56	0,001	up
A0A480PH82	O43719	I3LC46	HTATSF1	HIV Tat-specific factor 1	1,57	0,040	up
Q06AA9	P62837	Q06AA9	UBE2D2	Ubiquitin-conjugating enzyme E2 D2	1,57	0,039	up
A0A287A2D7	Q15643	A0A287A2D7	TRIP11	Thyroid hormone receptor interactor 11	1,62	0,047	up

Supplementary Table S3C: List of differential proteins identified comparing diets

Accession	Acceso UniProt Human	Acceso UniProt Pig	Gen	Identificación	FC	p-va-lor	Ctrl vs HFD
A0A286ZYE1	Q8IV36	F1RVV6	HID1	HID1 domain containing	0,56	0,001	down
A0A4X1W3R7	Q8WZ74	F1SJF2	CTTNBP2	Cortactin-binding protein 2	0,62	0,017	down
A0A4X1TPC2	P62750	A0A4X1TPC2	RPL23A	60S ribosomal protein L23a	0,62	0,011	down
A0A4X1VIL0	P21246	P79281	PTN	Pleiotrophin	0,63	0,044	down
A0A4X1V053	O75077	F1SHD2	ADAM23	ADAM metallopeptidase domain 23	0,66	0,022	down
A0A481DW99	Q6UWR7	F1RSZ8	ENPP6	Choline-specific glycerophosphodiester phosphodies-terase	1,51	0,012	up
A0A480N196	Q8WVM8	F1SHF4	SCFD1	Sec1 family domain-containing protein 1 isoform a	1,52	0,019	up
A0A4X1W5X8	O43657	F1S1M4	TSPAN6	Tetraspanin	1,53	0,016	up

Table S4. List of differentially abundant proteins in the hippocampus comparing NBW-Ctrl versus NBW-HFD and LBW-Ctrl versus LBW-HFD.

Accession	Uniprot Human	Uniprot Pig	gene	Description	FC	P.Value	CLBW vs HFDLBW	FC	P.Value	CNBW vs HFDNBW
A0A4X1V8T0	Q9UKU7	A0A4X1V8T0	ACAD8	Isobutyryl-CoA dehydrogenase, mitochondrial	0,5825	0,014	down	0,75	0,1872	
A0A480R7G2	P42025	F1STD5	ACTR1B	Beta-centractin	0,6421	0,027	down	0,88	0,5151	
A0A4X1SMM1	Q9UEY8	F1S5M9	ADD3	Gamma-adducin	0,5596	0,0096	down	0,88	0,5409	
A0A5K1VI30	Q12904	A0A5K1VI30	AIMP1	Aminoacyl tRNA synthetase complex interacting multifunctional protein 1	0,5848	0,0308	down	0,67	0,109	
A0A480VDF1	Q99996	I3LR05	AKAP9	A-kinase anchoring protein 9	0,6105	0,0147	down	1,18	0,394	
A0A480X1N8	P49419	F1RKM1	ALDH7A1	Alpha-aminoadipic semialdehyde dehydrogenase	0,5065	0,0409	down	0,78	0,4527	
F1SQT8	Q7Z6G8	F1SQT8	ANKS1B	Ankyrin repeat and sterile alpha motif domain-containing protein 1B	0,5902	0,0072	down	1,21	0,3207	
A0A480SXF6	Q06481	C7C1H8	APLP2	Amyloid-like protein 2 isoform 1	0,6285	0,0228	down	0,95	0,8027	
A0A4X1T255	P15848	A0A4X1T255	ARSB	Arylsulfatase B	0,6131	0,0126	down	1,06	0,7406	
A0A287B1K5	Q9HD20	F1S7C4	ATP13A1	ATPase 13A1	0,6204	0,03	down	1,24	0,3264	
A0A4X1UPW6	Q9BSU1	A0A287BJH7	C16orf70	Chromosome 6 C16orf70 homolog	0,6564	0,044	down	1,02	0,9295	
A0A4X1W5K9	P47756	A0PFK7	CAPZB	F-actin-capping protein subunit beta	0,5468	0,0027	down	0,96	0,8238	

A0A5G2QA46	Q6P1N0	F1SD63	CC2D1A	Coiled-coil and C2 domain containing 1A	0,58	0,0343	down	1,15	0,5774	
A0A4X1U1H8	O14735	D0G6R6	CDIPT	CDP-diacylglycerol--inositol 3-phosphatidyltransferase	0,5607	0,0146	down	1,28	0,2964	
A0A4X1THF5	Q00536	K7GRV3	CDK16	Cyclin dependent kinase 16	0,6157	0,049	down	0,81	0,4033	
A0A4X1T669	Q5SW79	I3LU72	CEP170	Centrosomal protein of 170 kDa	0,5796	0,0015	down	0,78	0,1189	
A0A481CY36	O43633	I3LSS2	CHMP2A	Charged multivesicular body protein 2a	0,6093	0,0388	down	0,88	0,5959	
A0A4X1T0Z2	Q8WUD6	A0A287B7T8	CHPT1	Choline phosphotransferase 1	0,5465	0,0148	down	0,84	0,465	
A0A5G2RB82	Q96S66	F1S5Y7	CLCC1	Chloride channel CLIC-like protein 1	0,5957	0,0332	down	1,06	0,8104	
A0A4X1U103	P99999	P62895	CYCS	Cytochrome c	0,6326	0,0019	down	1,03	0,8175	
A0A5G2Q8X4	O75935	F1SEC0	DCTN3	Dynactin subunit 3	0,6624	0,034	down	1,31	0,1757	
A0A286ZM27	P17844	A0A286ZM27	DDX5	DEAD box protein 5	0,6044	0,0232	down	1,13	0,5735	
B9WZD6	O95786	Q9GLV6	DDX58	Antiviral innate immune response receptor RIG-I	0,5406	0,0094	down	0,85	0,4839	
A0A5G2R656	Q8TB45	A0A480DP04	DEPTOR	DEP domain-containing mTOR-interacting protein	0,5379	0,0009	down	1,28	0,1496	
A0A4X1TKE5	Q9Y6T7	F1SF62	DGKB	Diacylglycerol kinase beta	0,6047	0,009	down	1,11	0,5754	
A0A4X1SNQ3	O60610	F1RMT5	DIAPH1	Diaphanous related formin 1	0,5626	0,0076	down	0,91	0,6321	

A0A4X1UT57	Q9BZ29	I3LTY2	DOCK9	Dedicator of cytokinesis 9	0,5785	0,012	down	0,99	0,955	
F1S2X3	Q9NTX5	F1S2X3	ECHDC1	Ethylmalonyl-CoA decarboxylase 1	0,6649	0,0274	down	1,06	0,7539	
A0A5G2Q8W0	O00423	I3LKT2	EML1	Echinoderm microtubule-associated protein-like 1	0,6281	0,0297	down	1,05	0,8344	
I3LDY1	O00423	I3LDY1	EML1	Echinoderm microtubule-associated protein-like 1	0,6235	0,0308	down	0,91	0,6512	
A0A481BSH3	A0A0U1RRM6	F1S8S4	ENAH	Protein enabled homolog isoform a	0,6092	0,0388	down	0,91	0,6978	
A0A480SB04	Q96RT1	F1SKS3	ERBIN	Erbin isoform 1	0,49	0,021	down	1,1	0,7418	
F2Z5J5	P84090	P80230	ERH	Enhancer of rudimentary homolog	0,606	0,0007	down	0,79	0,0859	
A0A4X1W2F0	Q9Y2D4	I3LRV0	EXOC6B	Exocyst complex component 6B	0,6655	0,0123	down	0,98	0,8868	
A0A5K1VT17	Q9Y6X4	F1S2J5	FAM169A	Family with sequence similarity 169 member A	0,5666	0,004	down	0,79	0,2067	
A0A287BEG6	Q9BXM9	F1SP51	FSD1L	Fibronectin type III and SPRY domain containing 1 like	0,389	0,0028	down	0,92	0,7851	
F1SP51	Q9BXM9	F1SP51	FSD1L	Fibronectin type III and SPRY domain containing 1 like	0,5391	0,0045	down	1,04	0,8628	
A0A4X1VXR8	P04066	I3LNS9	FUCA1	Alpha-L-fucosidase	0,5916	0,0201	down	1,01	0,9734	
A0A4X1SFJ5	P23434	A0A4X1SFJ5	GCSH	Glycine cleavage system H protein, mitochondrial	0,6552	0,0064	down	1,17	0,2881	
A0A4X1SRA1	Q969S9	F1S2J6	GFM2	Ribosome-releasing factor 2, mitochondrial	0,5677	0,0381	down	0,87	0,5966	

F1SPM7	Q06210	F1SPM7	GFPT1	Glutamine--fructose-6-phosphate transaminase (isomerizing)	0,5783	0,0447	down	0,8	0,4259	
P12309	P35754	P12309	GLRX	Glutaredoxin-1	0,6597	0,0215	down	0,7	0,0541	
A0A4X1UEN1	Q96IJ6	I3LUP1	GMPPA	Mannose-1-phosphate guanyltransferase alpha	0,5159	0,0371	down	0,78	0,4328	
F1S8F1	P30679	F1S8F1	GNA15	G protein subunit alpha 15	0,4823	0,0154	down	1,02	0,9376	
A0A4X1TDL2	O14653	A0A287BIC8	GOSR2	Golgi SNAP receptor complex member 2 isoform A	0,6253	0,0274	down	1,04	0,8578	
A0A4X1THI5	P48058	I3L8N9	GRIA4	Glutamate receptor 4	0,6233	0,0165	down	1,08	0,7034	
A0A4X1UTG0	P61978	I3LQS0	HNRNPK	Heterogeneous nuclear ribonucleoprotein K	0,6253	0,0138	down	0,95	0,7707	
A0A5G2QIL6	Q5JU85	F1RUI3	IQSEC2	IQ motif and Sec7 domain ArfGEF 2	0,4976	0,0072	down	1,09	0,7278	
A0A287AXX5	Q5VWX1	F1RZV6	KHDRBS2	KH RNA binding domain containing, signal transduction associated 2	0,5567	0,005	down	1,29	0,2112	
A0A4X1VAK5	Q9UIC8	F1RFE3	LCMT1	Leucine carboxyl methyltransferase 1	0,5426	0,0243	down	1,06	0,84	
F1RTT5	Q9NUN5	F1RTT5	LMBRD1	LMBR1 domain containing 1	0,6497	0,036	down	0,88	0,5243	
A0A4X1U7M0	P32455	F1S4D7	LOC100523668	GB1/RHD3-type G domain-containing protein	0,6571	0,0261	down	1,14	0,4843	
A0A4X1U8J5	Q96NW7	F1S832	LRRC7	Leucine-rich repeat-containing protein 7	0,6569	0,0266	down	0,96	0,8413	
A0A4X1U4R2	Q9NZL9	I3LU04	MAT2B	Methionine adenosyltransferase 2 subunit beta	0,5606	0,0296	down	0,89	0,6619	

A0A4X1SEK9	P02795	P79379	MT2A	Metallothionein-2	0,636	0,0298	down	0,96	0,8302	
A0A480WKW9	Q765P7	A0A480WKW9	MTSS2	MTSS 2 protein	0,6497	0,0079	down	0,86	0,3254	
Q71KI1	P03886	O79874	NADH1	NADH-ubiquinone oxidoreductase chain 1	0,4654	0,0021	down	0,82	0,3784	
A0A5G2R6P9	Q56VL3	F1SE82	OCIAD2	OCIA domain containing 2	0,5056	0,0271	down	1,01	0,9664	
A0A5G2Q930	O14737	F1RNX2	PDCD5	Programmed cell death 5	0,5658	0,0046	down	1,06	0,7513	
A0A480ME27	Q07343	F1S820	PDE4B	cAMP-specific 3',5'-cyclic phosphodiesterase 4B	0,5981	0,0137	down	1,12	0,5701	
A0A287BI36	Q96HC4	F1RWW4	PDLIM5	PDZ and LIM domain 5	0,6276	0,0321	down	0,85	0,4635	
A0A4X1TYE8	Q6P996	A0A287B2K7	PDXDC1	Pyridoxal-dependent decarboxylase domain-containing protein 1	0,6151	0,0296	down	0,94	0,7604	
A0A4X1TMK4	Q96S52	I3LRK9	PIGS	Phosphatidylinositol glycan anchor biosynthesis class S	0,6592	0,0363	down	0,89	0,5583	
A0A4X1V428	Q8NEB9	Q5D891	PIK3C3	Phosphatidylinositol 3-kinase catalytic subunit type 3	0,664	0,0385	down	1,36	0,1297	
A0A481B2C1	Q99570	K9IWD2	PIK3R4	Phosphoinositide 3-kinase regulatory subunit 4	0,5125	0,0285	down	1,03	0,9151	
A0A4X1SPN4	Q4KWH8	I3LFF0	PLCH1	Phosphoinositide phospholipase C	0,587	0,0139	down	1,12	0,602	
A0A4X1W4L6	Q92871	F1SRD8	PMM1	Phosphomannomutase	0,6384	0,0266	down	1,37	0,126	
A0A4X1W406	Q8WUY3	A0A286ZRA1	PRUNE2	Protein prune homolog 2	0,5124	0,0288	down	0,67	0,1964	

A0A480MP33	E7ESA6	F1RJS6	PTK2	Non-specific protein-tyrosine kinase	0,4862	0,0175	down	0,7	0,2323	
F1S725	Q5R372	F1S725	RABGAP1L	Rab GTPase-activating protein 1-like	0,5714	0,0215	down	0,94	0,7866	
I3LI59	Q9Y5S9	I3LI59	RBM8A	RNA-binding protein 8A	0,6149	0,0162	down	1,15	0,4911	
A0A5K1U3A5	P51812	F1SQN4	RPS6KA3	Ribosomal protein S6 kinase alpha-3	0,6238	0,0332	down	1,13	0,58	
A0A4X1UGB7	Q8N122	I3L942	RPTOR	Regulatory associated protein of MTOR complex 1	0,5457	0,0356	down	1,57	0,1282	
A0A287BB01	O95197	F1RPY3	RTN3	Reticulon 3	0,5866	0,0297	down	0,98	0,9327	
F1RT47	Q8WWV3	F1RT47	RTN4IP1	Reticulon 4 interacting protein 1	0,6351	0,0378	down	0,88	0,5712	
A0A4X1W0M9	P29034	K7GQ95	S100A2	S100 calcium binding protein A2	0,4401	0,0487	down	1,55	0,2962	
F1RM03	Q9UBE0	F1RM03	SAE1	SUMO1 activating enzyme subunit 1	0,5487	0,0257	down	0,74	0,2624	
A0A287AE16	Q9Y6U3	Q29297	SCIN	Adseverin	0,5146	0,0333	down	0,97	0,9199	
I3L8U8	Q0VDG4	I3L8U8	SCRN3	Secernin 3	0,6174	0,0266	down	1,08	0,7089	
A0A4X1SGW5	P60468	F2Z5P0	SEC61B	SEC61 translocon subunit beta	0,5835	0,0266	down	1,01	0,9504	
A0A5G2QSI1	Q99442	I3LND2	SEC62	Translocation protein SEC62	0,6071	0,0366	down	0,99	0,972	
A0A4X1TLR3	Q9NS82	F1RNW8	SLC7A10	Asc-type amino acid transporter 1	0,6035	0,0319	down	1,3	0,2731	

A0A5G2QZ94	Q92922	F1SLI6	SMARCC1	SWI/SNF complex subunit SMARCC1	0,6115	0,0394	down	0,97	0,9024	
A0A4X1VJH0	Q92673	I3L8K1	SORL1	Low-density lipoprotein receptor relative with 11 ligand-binding repeats	0,5468	0,0107	down	0,77	0,248	
H2B2M0	Q99523	F1S5Z7	SORT1	Sortilin 1	0,6252	0,0474	down	1,12	0,6458	
A0A4X1SVT4	Q08170	F6QB00	SRSF4	Serine/arginine-rich splicing factor 4 isoform X1	0,6516	0,0441	down	1,22	0,3581	
F1S3E0	Q9BVK6	F1S3E0	TMED9	Transmembrane emp24 domain-containing protein 9	0,6468	0,0159	down	0,83	0,2993	
A0A287BNI4	Q9BYE2	A0A287BNI4	TMPRSS13	Transmembrane serine protease 13	0,6578	0,025	down	0,8	0,2237	
A0A5G2QKH8	O96008	F1RM44	TOMM40	Translocase of outer mitochondrial membrane 40	0,5638	0,0208	down	1,1	0,6929	
A0A4X1TYJ1	P09493	P42639	TPM1	Tropomyosin alpha-1 chain	0,6572	0,0097	down	0,97	0,8685	
A0A5G2QFH4	P62995	Q06AA7	TRA2B	Transformer 2 beta homolog	0,6551	0,0237	down	1,05	0,8084	
F1RSJ3	Q96Q05	F1RSJ3	TRAPPC9	Trafficking protein particle complex 9	0,6506	0,0309	down	1,15	0,4693	
A0A287BA16	Q15643	A0A287BA16	TRIP11	Thyroid hormone receptor interactor 11	0,4833	0,01	down	0,79	0,3779	
A0A5G2QVF5	E7EWD5	P80220	TSC22D3	TSC22 domain family protein 3	0,4846	0,0039	down	1,36	0,195	
A7E1S5	Q9BQE3	A7E1S5	TUBA1C	Tubulin alpha-1C chain	0,6294	0,0223	down	1,22	0,3177	
A0A4X1TXV4	P26368	I3W8V7	U2AF2	Splicing factor U2AF 65 kDa subunit	0,6475	0,0309	down	0,81	0,3033	

A0A480L6C9	Q9NWZ5	M3UZ96	UCKL1	Uridine-cytidine kinase	0,625<br>3	0,042<br>1	down	0,9<br>2	0,711<br>8	
A0A4X1VNI8	P46939	K9J6K2	UTRN	Utrophin	0,630<br>7	0,000<br>7	down	0,9<br>4	0,626<br>7	
A0A287AE08	Q13443	F1RZL4	ADAM9	Disintegrin and metalloproteinase domain-containing protein 9	1,513<br>9	0,040<br>9	up	0,7<br>7	0,201<br>6	
A0A5G2R0R1	P55196	F1SBY5	AFDN	Afadin, adherens junction formation factor	1,530<br>2	0,029<br>7	up	0,8<br>3	0,348<br>8	
A0A4X1W927	O00468	I3LGD9	AGRN	Agrin	1,934<br>1	0,011<br>5	up	1,0<br>7	0,801<br>1	
A0A4X1UV89	Q12904	A0A4X1UV89	AIMP1	Aminoacyl tRNA synthase complex-interacting multifunctional protein 1	1,744<br>5	0,008<br>5	up	1,1	0,642<br>7	
A0A287A579	Q99996	I3LR05	AKAP9	A-kinase anchoring protein 9	1,840<br>1	0,018<br>3	up	1,2<br>4	0,393<br>8	
A0A4X1T259	P27216	F1RRP6	ANXA13	Annexin A13	1,862<br>4	0,008	up	1,1	0,657<br>7	
A0A4X1U4C2	Q92572	A0A4X1U4C2	AP3S1	AP-3 complex subunit sigma-1	1,702<br>8	0,010<br>7	up	1,0<br>4	0,843<br>9	
A0A480VYB2	P51689	I3LM95	ARSD	Arylsulfatase D isoform alpha	1,834	0,006<br>2	up	0,8<br>5	0,449<br>5	
A0A4X1UHM<br>9	O14983	F1RFH9	ATP2A1	Calcium-transporting ATPase	1,962<br>7	0,002<br>3	up	0,8<br>7	0,482<br>7	
A0A287ANF8	Q5TC12	A0A287ANF8	ATPAF1	ATP synthase mitochondrial F1 complex assembly factor 1	1,659<br>1	0,031	up	0,9<br>5	0,835<br>2	
A0A4X1U9P0	Q9H425	F1RG57	C14H1orf198	Chromosome 14 C1orf198 homolog	1,730<br>7	0,007	up	0,8<br>1	0,274<br>5	
A0A5G2QMX2	Q15878	F1S675	CACNA1E	Voltage-dependent R-type calcium channel subunit alpha	1,886<br>2	0,005	up	1,2	0,387<br>3	

A0A5S8KVN2	P30988	P25117	CALCR	Calcitonin receptor	1,7013	0,015	up	0,99	0,9742	
A0A4X1U688	Q9HA77	A0A4X1U688	CARS2	Cysteinyl-tRNA synthetase	1,5337	0,0413	up	0,96	0,8421	
A0A4X1U5Q5	P16152	Q28960	CBR1	Carbonyl reductase [NADPH] 1	1,6412	0,0335	up	1,18	0,4828	
A0A287AYK3	P35520	A0A287AYK3	CBS	Cystathionine beta-synthase	1,7197	0,021	up	0,88	0,5716	
A0A287B7U3	Q3V6T2	F1SQK4	CCDC88A	Coiled-coil domain containing 88A	1,7055	0,026	up	0,86	0,5344	
F1RY11	Q567U6	F1RY11	CCDC93	Coiled-coil domain containing 93	1,5149	0,0137	up	0,97	0,8747	
A0A4X1VWW9	O96005	F1RM47	CLPTM1	CLPTM1 regulator of GABA type A receptor forward trafficking	1,7063	0,0136	up	0,9	0,6022	
A0A4X1TFA8	Q9H9Q2	A0A4X1TFA8	COPS7B	COP9 signalosome complex subunit 7b	1,6966	0,0036	up	1,28	0,1503	
A0A4X1VJ44	Q9NZJ6	I3LD55	COQ3	Ubiquinone biosynthesis O-methyltransferase, mitochondrial	1,8445	0,0167	up	1,36	0,2258	
A0A5G2QQG0	O95825	I3LQA0	CRYZL1	Crystallin zeta like 1	2,0633	0,0033	up	0,75	0,2252	
A0A4X1TVF2	Q9UQB3	F1SRN5	CTNND2	Catenin delta-2	1,664	0,0223	up	0,89	0,6018	
A0A4X1TMF2	Q9UBP4	F6Q5E9	DKK3	Dickkopf_N domain-containing protein	1,5258	0,0357	up	0,76	0,1858	
A0A286ZVQ8	P25686	F1SR79	DNAJB2	DnaJ homolog subfamily B member 2	1,7499	0,026	up	1,03	0,9031	
A0A480Q1W3	Q96BY6	F1SNP7	DOCK10	Dedicator of cytokinesis protein 10 isoform X9-like	1,7608	0,0263	up	1,05	0,8373	

A0A480X0S5	F6QMI7	A0A480X0S5	DST	Dystonin isoform 1eA	1,5181	0,0293	up	1,12	0,5545	
Q9GLD4	P49327	I3LCW1	FASN	Fatty acid synthase (Fragment)	1,5453	0,026	up	0,86	0,4492	
A0A480N6P3	Q5T1M5	F1SN89	FKBP15	FK506-binding protein 15	1,9791	0,029	up	0,78	0,4221	
A0A4X1SPU0	Q969S9	F1S2J6	GFM2	Ribosome-releasing factor 2, mitochondrial	1,5005	0,035	up	1	0,989	
A0A480PE19	P40939	Q29554	HADHA	Trifunctional enzyme subunit alpha, mitochondrial	2,1016	0,0018	up	1,21	0,3793	
A4GUC2	P26012	A0A287AUF9	ITGB8	Integrin beta-8	1,7104	0,0037	up	1,22	0,2605	
A0A4X1T9U1	Q8WXH2	F1S6K2	JPH3	Junctophilin	1,7168	0,0207	up	1,17	0,4957	
A0A4X1SNV8	O75525	K7GNY3	KHDRBS3	KH domain-containing, RNA-binding, signal transduction-associated protein 3	1,52	0,0321	up	1,03	0,8594	
A0A287BNN8	Q9UPQ0	F1S4G7	LIMCH1	LIM and calponin homology domains 1	1,8042	0,0132	up	0,85	0,4963	
A0A287AUT0	P32455	F1S4D7	LOC100523668	GB1/RHD3-type G domain-containing protein	1,5751	0,0238	up	1,14	0,5165	
A0A4X1TYC3	P53779	K7GP94	MAPK10	Mitogen-activated protein kinase	1,8717	0,0325	up	1,72	0,0744	
A0A5G2QN98	Q9NZL9	I3LU04	MAT2B	Methionine adenosyltransferase 2 subunit beta	2,0785	0,0044	up	1,12	0,6366	
A0A5G2R4H0	Q96QG7	A0A5G2R4H0	MTMR9	Myotubularin-related protein 9	1,5225	0,0309	up	1,35	0,1294	
B5KN59	P03886	O79874	NADH1	NADH-ubiquinone oxidoreductase chain 1	1,6304	0,0362	up	0,93	0,7643	

F1RTV5	P22234	F1RTV5	PAICS	AIR carboxylase	1,638	0,0418	up	0,94	0,8098	
A0A5G2QCR7	Q9NVD7	I3L973	PARVA	Parvin alpha	1,5745	0,0094	up	1,16	0,3712	
A0A480IMJ0	Q07343	F1S820	PDE4B	cAMP-specific 3',5'-cyclic phosphodiesterase 4B	1,5239	0,0361	up	1,11	0,5894	
F1RT56	Q15119	F1RT56	PDK2	Protein-serine/threonine kinase	1,6908	0,0073	up	1,03	0,8607	
A0A4X1SQG1	Q4KWH8	I3LFF0	PLCH1	Phosphoinositide phospholipase C	1,674	0,0138	up	1,39	0,1134	
F1SKF1	Q14651	F1SKF1	PLS1	Plastin 1	1,6062	0,0462	up	0,95	0,8407	
F1S2E3	P30405	F1S2E3	PPIF	Peptidyl-prolyl cis-trans isomerase F, mitochondrial	1,7124	0,0086	up	0,94	0,7679	
A0A480TS65	P50897	A0A287AYH9	PPT1	Palmitoyl-protein hydrolase 1	1,7554	0,0149	up	1,01	0,9553	
A0A4X1W0Y8	P28070	Q29384	PSMB4	Proteasome subunit beta type-4	1,604	0,0139	up	0,86	0,4345	
A0A287AMM5	Q3YEC7	F1RVY4	RABL6	RAB, member RAS oncogene family like 6	1,7324	0,0287	up	0,97	0,8892	
A0A286ZKD3	Q5XPI4	I3LFZ7	RNF123	E3 ubiquitin-protein ligase RNF123	1,5535	0,0379	up	0,75	0,1878	
A0A480YJW7	Q63HN8	A0A480YJW7	RNF213	E3 ubiquitin-protein ligase RNF213 isoform 3	2,3761	0,021	up	1,05	0,8988	
A0A5G2Q990	Q15404	F1RW98	RSU1	Ras suppressor protein 1	1,57	0,0215	up	1,13	0,5342	
A0A4X1WC18	Q9UQD0	F1SGK6	SCN8A	Sodium channel protein type 8 subunit alpha	1,727	0,0016	up	0,97	0,8605	

A0A4X1SHX2	P01009	P50447	SERPINA1	Alpha-1-antitrypsin	1,6324	0,0357	up	1,27	0,3136	
A0A5G2R349	Q86TU7	F1SAQ4	SETD3	Protein-histidine N-methyltransferase	1,8075	0,0169	up	1,08	0,75	
A0A4X1VJI2	Q9NXA8	A7XXV9	SIRT5	NAD-dependent protein deacylase sirtuin-5, mitochondrial	1,9278	0,0394	up	0,82	0,5374	
F1SLF4	Q6GMV2	F1SLF4	SMYD5	SMYD family member 5	1,7032	0,0146	up	1	0,9992	
A0A4X1TNU2	Q13247	A0A4X1TTZ9	SRSF6	Serine/arginine-rich splicing factor 6	1,9443	0,0328	up	1,12	0,7156	
A0A4X1T3H7	O14994	F1SPS2	SYN3	Synapsin-3	1,5269	0,0053	up	1,18	0,2506	
A0A480IXS3	Q3LXA3	F1RKQ4	TKFC	Triokinase/FMN cyclase	1,6925	0,0026	up	1,07	0,6678	
A0A480S340	Q3LXA3	F1RKQ4	TKFC	Triokinase/FMN cyclase isoform b	1,622	0,0029	up	0,86	0,3069	
A0A287AGM1	Q9H330	F1SP25	TMEM245	Transmembrane protein 245	1,8372	0,0208	up	0,85	0,5417	
A0A4X1TDU5	Q6ZMR5	A0A287BEC5	TMPRSS11A	Transmembrane protease serine	1,7995	0,0184	up	1,1	0,6996	
A0A4X1UJK3	Q9H1E5	I3LB23	TMX4	Thioredoxin domain-containing protein	1,5452	0,015	up	1,16	0,384	
A0A5G2QZ59	Q5T0D9	F1RJA8	TPRG1L	Tumor protein p63 regulated 1 like	1,6884	0,0206	up	1,26	0,2994	
A0A4X1SDS7	Q9C026	F1SFF7	TRIM9	E3 ubiquitin-protein ligase TRIM9	1,8228	0,0355	up	0,71	0,2397	
A0A5G2QE60	E7EWD5	P80220	TSC22D3	TSC22 domain family protein 3	1,9149	0,0162	up	0,95	0,8486	

A0A480UBM5	Q71U36	P02550	TUBA1A	Tubulin alpha-1A chain	1,5567	0,0375	up	0,82	0,3644	
A0A4X1URM2	Q9NNW7	F1RHN4	TXNRD2	Thioredoxin reductase 2	1,6549	0,0093	up	0,84	0,3451	
A0A5G2R434	Q9NNW7	F1RHN4	TXNRD2	Thioredoxin-disulfide reductase	2,2676	0,0087	up	0,95	0,8623	
A0A286ZRI2	Q92890	F1RK61	UFD1	Ubiquitin recognition factor in ER associated degradation 1	1,5913	0,0185	up	1	0,9964	
A0A5G2RB53	Q92890	F1RK61	UFD1	Ubiquitin recognition factor in ER associated degradation 1	1,7688	0,0486	up	0,76	0,343	
A0A480IWZ9	Q8N1B4	F1RZT5	VPS52	Vacuolar protein sorting-associated protein 52 homolog	1,6196	0,0414	up	1,18	0,492	
A0A480U566	Q9H1Z4	I3LPL0	WDR13	WD repeat-containing protein 13 isoform X1	1,5992	0,0235	up	0,93	0,7131	
K7GNH1	Q9Y484	K7GNH1	WDR45	WD repeat domain phosphoinositide-interacting protein 4	1,5725	0,0335	up	1,1	0,6454	
A0A287A6F5	Q8N4Q0	A0A287A6F5	ZADH2	Prostaglandin reductase 3 isoform 1	2,0162	0,0004	up	1,08	0,655	
I3LGD9	O00468	I3LGD9	AGRN	Agrin	1,0664	0,7173		0,67	0,0413	down
A0A5G2R290	P31751	G9BWQ2	AKT2	RAC-beta serine/threonine-protein kinase	0,8682	0,4163		0,64	0,025	down
A0A4X1UHV7	P47895	F1SR94	ALDH1A3	Aldehyde dehydrogenase family 1 member A3	0,8613	0,3977		0,65	0,0283	down
A0A5G2RC09	O00203	F1S2G1	AP3B1	AP-3 complex subunit beta	0,9834	0,9333		0,6	0,027	down
A0A4X1TEA2	P07741	F1S6K5	APRT	Adenine phosphoribosyltransferase	1,1145	0,5973		0,62	0,0387	down

A0A4X1SNI5	Q17R89	F1SS49	ARHGAP44	Rho GTPase activating protein 44	0,9037	0,6268		0,62	0,0421	down
A0A4X1SU43	O95817	F1S415	BAG3	BAG family molecular chaperone regulator 3	0,8221	0,2048		0,64	0,0112	down
A0A5G2QBS9	P15056	F1SRQ1	BRAF	B-Raf proto-oncogene, serine/threonine kinase	1,1065	0,6818		0,58	0,0464	down
I3LDN0	Q8IYJ2	I3LDN0	C10H10orf67	Chromosome 10 C10orf67 homolog	0,9488	0,7775		0,63	0,0284	down
A0A5G2QRL3	Q86YS7	F1SFI8	C2CD5	C2 calcium dependent domain containing 5	1,2185	0,3362		0,57	0,015	down
A0A4X1SYE0	P22748	F1S1C3	CA4	Carbonate dehydratase IV	0,8956	0,5166		0,59	0,0079	down
A0A4X1VHY6	A0A2R8YEJ7	A0A4X1VHY6	CASK	Peripheral plasma membrane protein CASK	0,8511	0,3294		0,67	0,0277	down
A2TNA7	Q00535	Q197W4	CDK5	Cyclin-dependent kinase 5 (Fragment)	1,122	0,4794		0,61	0,0093	down
A5PF00	P00751	K7GPT9	CFB	C3/C5 convertase	0,8789	0,496		0,64	0,0363	down
A0A481B434	P17540	Q2HYU1	CKMT2	Creatine kinase	1,0318	0,8898		0,56	0,0237	down
A0A480YJ16	P02462	M3V819	COL4A1	Collagen alpha-1(IV) chain isoform 1 preproprotein	1,0163	0,9105		0,65	0,0096	down
A0A4X1WA40	P61923	F2Z5S7	COPZ1	Coatomer subunit zeta	0,9509	0,8055		0,61	0,0304	down
A0A481D7D0	Q7KZN9	F1S8W1	COX15	Cytochrome c oxidase assembly protein COX15 homolog isoform 1 (Fragment)	1,1448	0,4453		0,54	0,0036	down
A0A4X1UVX3	P14406	F1S4V0	COX7A2	Cytochrome c oxidase subunit 7A2, mitochondrial	1,0877	0,5736		0,65	0,012	down

A0A287BRT8	Q08257	Q0MVN8	CRYZ	Quinone oxidoreductase	0,9302	0,7623		0,49	0,0108	down
Q95KR0	P04080	Q29290	CSTB	Cystatin B (Fragment)	0,9648	0,8392		0,49	0,001	down
A0A4X1W3D2	Q8WZ74	F1SJF2	CTTNBP2	Cortactin-binding protein 2	1,2309	0,2793		0,65	0,0392	down
A0A480Z928	P52429	F1RSC5	DGKE	Diacylglycerol kinase epsilon	1,0905	0,6197		0,62	0,0164	down
A0A4X1UUC4	Q8WW22	F6PUU1	DNAJA4	DnaJ homolog subfamily A member 4	1,2714	0,234		0,65	0,0494	down
F1SMM0	Q7Z2K6	F1SMM0	ERMP1	Endoplasmic reticulum metallopeptidase 1	1,1092	0,6275		0,62	0,0442	down
A0A287BFP9	Q96EK7	I3LUV2	FAM120B	Family with sequence similarity 120B	1,2293	0,2052		0,66	0,0221	down
F1RX36	P02671	F1RX36	FGA	Fibrinogen alpha chain	0,8711	0,1693		0,65	0,0006	down
A0A480X0T9	Q13045	A0A287AT54	FLII	Protein flightless-1 homolog isoform 1	0,9306	0,7801		0,53	0,0275	down
A0A4X1VNG0	P49448	P42174	GLUD1	Glutamate dehydrogenase 1, mitochondrial	0,9144	0,5944		0,65	0,0256	down
A0A4X1W412	P15586	K9IVU5	GNS	N-acetylglucosamine-6-sulfatase	0,8032	0,3792		0,54	0,0278	down
A0A4X1T0S7	P09211	P80031	GSTP1	GST class-pi	0,9495	0,8116		0,48	0,0045	down
A0A4X1U4D2	Q8NBQ5	I3L5K3	HSD17B13	Estradiol 17-beta-dehydrogenase 11	0,9547	0,7586		0,6	0,0045	down
A0A4X1UX13	Q8NFR9	A0A4X1UX13	IL17RE	Interleukin-17 receptor E	0,9959	0,9914		0,4	0,0341	down

A0A4X1WB54	Q13683	A0A481B0D0	ITGA2	Integrin alpha-2	0,9583	0,8138		0,63	0,0236	down
A0A480NE93	Q14643	I3LHZ3	ITPR1	Inositol 1,4,5-trisphosphate receptor type 1 isoform	1,1821	0,3454		0,64	0,0259	down
A0A4X1UVT4	Q9BVA0	F1RF38	KATNB1	Katanin p80 WD40 repeat-containing subunit B1	1,1564	0,468		0,6	0,0246	down
A0A287B836	P13473	F1RUC0	LAMP2	Lysosomal associated membrane protein 2	1,3409	0,1598		0,59	0,022	down
A0A5G2QCP8	P46734	K7GNK7	MAP2K3	Dual specificity mitogen-activated protein kinase kinase 3	1,123	0,5969		0,57	0,0255	down
I3LSP1	P50579	I3LSP1	METAP2	Methionine aminopeptidase 2	0,9705	0,8775		0,56	0,0104	down
A0A4X1W6X4	Q9H8H3	A0A287A6C1	METTL7A	Methyltransferase-like protein 7A	0,8092	0,267		0,56	0,0076	down
A0A4X1U8A7	Q7RTP6	F1SHR1	MICAL3	F-actin monooxygenase	1,0451	0,8346		0,62	0,0444	down
A0A4X1T9M8	P53602	M3VH72	MVD	Diphosphomevalonate decarboxylase	1,0825	0,7087		0,6	0,033	down
A0A5S6I9M5	P20592	A7VK00	MX2	Interferon-induced GTP-binding protein Mx2	1,1895	0,3887		0,52	0,006	down
Q1WEZ8	P03886	O79874	NADH1	NADH-ubiquinone oxidoreductase chain 1	0,9679	0,8756		0,55	0,0144	down
A0A481CVT6	Q9NXR1	I3L8G0	NDE1	Nuclear distribution protein nudE homolog 1	1,0645	0,6515		0,58	0,0015	down
A0A4X1TJL9	P21359	F1RJ58	NF1	Neurofibromin	0,9803	0,9026		0,65	0,0224	down
A0A4X1VGW0	Q8N0W4	A0A287BC42	NLGN4X	Neuroligin-4, X-linked	0,9394	0,739		0,57	0,0105	down

A0A4X1TFG8	P61916	O97763	NPC2	NPC intracellular cholesterol transporter 2	0,9821	0,9463		0,51	0,0257	down
A0A4X1WCA6	Q9ULB1	I3L8T0	NRXN1	Neurexin-1	0,991	0,9618		0,62	0,0276	down
A0A286ZUG8	P52948	F1SUZ2	NUP98	Nuclear pore complex protein Nup96	1,0627	0,6779		0,65	0,0106	down
A0A5G2QZ66	Q9HBI1	F1SJU4	PARVB	Parvin beta	1,2141	0,3532		0,6	0,0277	down
F1S3G5	Q13526	I3LLH5	PIN1	Peptidyl-prolyl cis-trans isomerase	0,8992	0,6617		0,49	0,0111	down
Q29582	P14618	F1SHM0	PKM	Pyruvate kinase	0,8699	0,399		0,58	0,0052	down
A0A287B6R6	Q4KWH8	I3LFF0	PLCH1	Phosphoinositide phospholipase C	0,8184	0,4536		0,54	0,0416	down
A0A4X1TVL0	O60256	F1SB18	PRPSAP2	Phosphoribosyl pyrophosphate synthase-associated protein 2	1,1466	0,4956		0,59	0,0218	down
A0A5G2QYV9	P20337	F1S6G3	RAB3B	RAB3B, member RAS oncogene family	1,01	0,9669		0,48	0,0092	down
A0A286ZVJ2	P61224	A0A286ZVJ2	RAP1B	Ras-related protein Rap-1b isoform 1	1,3417	0,2137		0,58	0,0377	down
A0A4X1V9V8	Q6JBY9	F1S269	RCSD1	RCSD domain containing 1	0,6304	0,2144		0,43	0,038	down
P62272	P62269	P62272	RPS18	40S ribosomal protein S18	0,9333	0,788		0,56	0,0435	down
Q29308	P39019	Q29308	RPS19	40S ribosomal protein S19	1,0811	0,6827		0,6	0,0182	down
A0A4X1U5L3	P10523	P79260	SAG	S-arrestin	0,7983	0,2384		0,64	0,0375	down

A0A480TTT7	Q15858	F1S1Y7	SCN9A	Sodium channel protein type 9 subunit alpha	1,1254	0,465		0,6	0,0066	down
F1RT28	Q9UGP8	F1RT28	SEC63	J domain-containing protein	0,9186	0,7673		0,49	0,0276	down
A0A286ZI11	E9PFN4	F1RRH2	SLC4A7	Anion exchange protein	1,1161	0,596		0,62	0,0426	down
A0A4X1V3P0	O00161	A0A4X1V3P0	SNAP23	Synaptosome associated protein 23	0,9099	0,6651		0,59	0,0341	down
A0A4X1U4N7	Q14515	F1RW32	SPARCL1	SPARC like 1	1,0563	0,8042		0,59	0,032	down
F1SNC1	Q9C093	Q2IA00	SPEF2	Sperm flagellar protein 2	1,0712	0,7246		0,58	0,0152	down
A0A4X1VHD8	P31948	I3LNG8	STIP1	Stress induced phosphoprotein 1	1,301	0,4294		0,46	0,0352	down
A0A4X1VGE1	P31948	I3LNG8	STIP1	Stress induced phosphoprotein 1	1,301	0,4294		0,46	0,0352	down
A0A287B141	Q96SI9	F1SKU5	STRBP	Spermatid perinuclear RNA-binding protein	0,88	0,4377		0,59	0,0069	down
C7S2Z6	P61956	P61958	SUMO2	Small ubiquitin-related modifier	0,9039	0,6		0,6	0,021	down
A0A480W7Z5	Q8NF91	A0A480W7Z5	SYNE1	Nesprin-1	1,3507	0,1853		0,6	0,0422	down
A0A4X1U4Q1	O14787	F1SEX5	TNPO2	Transportin-2	1,0647	0,7349		0,62	0,0219	down
A0A4X1UCK9	O14787	F1SEX5	TNPO2	Transportin-2	0,6917	0,0609		0,49	0,0018	down
A0A287AXL5	Q9Y5L0	F1SMQ0	TNPO3	Transportin 3	0,9757	0,8706		0,62	0,0068	down

F1RPD2	P22695	F1RPD2	UQCRC2	Cytochrome b-c1 complex subunit 2, mitochondrial	0,7791	0,3259		0,54	0,0307	down
A0A5G2QXB6	Q9Y484	K7GNH1	WDR45	WD repeat domain phosphoinositide-interacting protein 4	1,1588	0,432		0,64	0,0317	down
A0A4X1WD73	O00471	F1SSM0	EXOC5	Exocyst complex component 5	0,6248	0,0449	down	0,61	0,0431	down
I3LIX0	Q08379	I3LIX0	GOLGA2	Golgin subfamily A member 2	0,6271	0,0106	down	0,57	0,023	down
A0A5G2R265	Q16773	F1RR62	KYAT1	Kynurenine--oxoglutarate transaminase 1	0,577	0,0199	down	0,55	0,0042	down
A0A4X1ULI8	O00214	F1RGZ0	LGALS8	Galectin	0,6104	0,0162	down	0,56	0,0201	down
A0A4X1SZ93	Q9NUP9	F2Z5M5	LIN7C	Protein lin-7 homolog C	0,6444	0,0147	down	0,51	0,0325	down
A0A4X1VX77	O43920	F1SV23	NDUFS5	Complex I-15 kDa	0,6608	0,0263	down	0,66	0,0347	down
I3LEB7	Q7Z5L7	I3LEB7	PODN	Podocan	0,6239	0,0376	down	0,55	0,0152	down
A0A287APM1	Q8IX01	I3LFJ5	SUGP2	SURP and G-patch domain containing 2	0,5852	0,0117	down	0,65	0,0234	down
F1RH13	Q9BQG1	F1RH13	SYT3	dolichyl-diphosphooligosaccharide--protein glycotransferase	0,5878	0,0291	down	0,58	0,0358	down
A0A4X1VM20	O43307	F1RTP2	ARHGEF9	Collybistin	1,7631	0,0147	up	0,47	0,0264	down
A0A4X1UET6	Q9GZY4	I3LR62	COA1	Cytochrome c oxidase assembly factor 1 homolog	1,5842	0,0297	up	0,63	0,0056	down
A0A5G2RIY7	Q99627	F1SM07	COPS8	COP9 signalosome complex subunit 8	1,6187	0,0402	up	0,48	0,0234	down

F1SNT8	Q14203	F1SNT8	DCTN1	Dynactin subunit 1	1,8315	0,0364	up	0,64	0,0232	down
A0A286ZL35	Q86UW8	A0A286ZL35	HAPLN4	Hyaluronan and proteoglycan link protein 4	1,5458	0,0486	up	0,63	0,0454	down
A0A480XZ08	Q6P996	A0A287B2K7	PDXDC1	Pyridoxal-dependent decarboxylase domain-containing protein 1	1,5931	0,0115	up	0,55	0,0207	down
A0A5G2QMU5	Q8NBX0	F1S8P1	SCCPDH	Saccharopine dehydrogenase-like oxidoreductase	1,5945	0,037	up	0,57	0,0179	down
A0A481AII0	Q6JQN1	F1RL48	ACAD10	Acyl-CoA dehydrogenase family member 10	0,8863	0,6218		2	0,0138	up
A0A286ZSR3	Q15067	F1RVZ1	ACOX1	Acyl-coenzyme A oxidase	1,2173	0,2701		1,58	0,0229	up
A0A480V7K8	O00116	I3LM15	AGPS	Alkylglycerone-phosphate synthase	1,1064	0,6161		2,24	0,0012	up
A0A287B6G6	O00468	I3LGD9	AGRN	Agrin	1,2562	0,2578		1,61	0,0324	up
A0A5G2QKQ8	P54819	A0A5G2QKQ8	AK2	Adenylate kinase 2, mitochondrial	0,9997	0,9986		1,57	0,0233	up
A0A5G2R903	Q8NEU8	F6Q7B0	APPL2	Adaptor protein, phosphotyrosine interacting with PH domain and leucine zipper 2	1,2347	0,356		1,64	0,0498	up
A0A286ZTA6	P15289	A0A286ZTA6	ARSA	Arylsulfatase A	1,2013	0,2107		1,55	0,0094	up
A0A4X1SV97	Q9NT62	F1SLS7	ATG3	Autophagy related 3	1,1566	0,5079		1,79	0,0202	up
A0A5G2Q8U4	Q9Y4P1	D7RA22	ATG4B	Cysteine protease	0,973	0,9075		1,69	0,0468	up
F1SMF9	O75947	F1SMF9	ATP5PD	ATP synthase subunit d, mitochondrial	1,2131	0,377		1,76	0,0221	up

A0A287AKI0	Q9NYF8	F1S684	BCLAF1	BCL2 associated transcription factor 1	0,7836	0,2797		1,71	0,0332	up
F1SQF8	Q14012	F1SQF8	CAMK1	Calcium/calmodulin dependent protein kinase I	1,1292	0,5171		1,6	0,0271	up
B5L0Y3	P20810	P12675	CAST	Calpastatin	1,0283	0,8747		1,76	0,0067	up
A0A4X1VEM1	O43303	I3LAA1	CCP110	Centriolar coiled-coil protein 110	1,5324	0,1349		2,12	0,0178	up
A0A481BMX2	Q9HCU4	A0A287AEM7	CELSR2	Cadherin EGF LAG seven-pass G-type receptor 2	0,8469	0,3496		1,5	0,0386	up
A0A4X1VKX5	Q99807	A0A4X1VKX5	COQ7	5-demethoxyubiquinone hydroxylase, mitochondrial	0,8569	0,5251		1,75	0,0392	up
A0A5G2QL31	P14854	A0A5G2QL31	COX6B1	Cytochrome c oxidase subunit 6B1	1,2226	0,2889		1,7	0,0142	up
A0A5G2QHM6	Q86T65	F1RVQ5	DAAM2	Dishevelled associated activator of morphogenesis 2	1,0096	0,9642		1,68	0,0317	up
A0A0J9X295	Q13561	F1SKF9	DCTN2	Dynactin subunit 2	0,9992	0,9972		1,81	0,0191	up
A0A4X1VR28	Q92841	F1SKQ0	DDX17	Probable ATP-dependent RNA helicase DDX17	1,1322	0,6624		2,05	0,0259	up
A0A4X1W5L2	Q8NF50	M3TYM9	DOCK8	Dedicator of cytokinesis protein 8	0,8834	0,5414		1,69	0,0225	up
A0A5K1U3Q4	Q6V1X1	K7GN07	DPP8	Dipeptidyl peptidase 8	0,9658	0,8997		1,91	0,0371	up
A0A480JST9	O95163	F1SP27	ELP1	Elongator complex protein 1	0,99	0,9487		1,59	0,0107	up
A0A481DE00	Q96RT1	F1SKS3	ERBIN	Erbin isoform 2	1,0362	0,8274		1,55	0,019	up

A0A5G2RGJ1	Q9Y4F1	I3LEG0	FARP1	FERM, ARH/RhoGEF and pleckstrin domain protein 1	0,7946	0,2357		1,61	0,0281	up
B3GLY4	Q6P1M0	B3GLY4	FATP4	Fatty acid transport protein 4 (Fragment)	1,0284	0,8837		1,62	0,0261	up
A0A5G2QUW9	Q96ME1	A0A5G2QUW9	FBXL18	F-box and leucine rich repeat protein 18	0,6318	0,1498		2,49	0,0114	up
A0A480VVI3	O94952	F1RKE9	FBXO21	F-box only protein 21	1,219	0,4183		1,96	0,0161	up
A0A480K4A9	Q13045	A0A287AT54	FLII	Protein flightless-1 homolog isoform 1 (Fragment)	1,4743	0,1406		1,95	0,0218	up
A0A4X1W3V6	Q5CZC0	A0A4X1W3V6	FSIP2	Fibrous sheath-interacting protein 2	0,9793	0,9254		1,67	0,0408	up
P19130	P02794	P19130	FTH1	Ferritin heavy chain	1,0119	0,9446		1,65	0,0119	up
A0A5G2R0N1	Q86UW8	A0A5G2R0N1	HAPLN4	Hyaluronan and proteoglycan link protein 4	1,3865	0,1424		1,88	0,0117	up
A0A4X1V6U1	P26583	P17741	HMGB2	High mobility group protein B2	1,2206	0,3201		1,6	0,035	up
A0A4X1V9E7	P09651	F6Q9I4	HNRNPA1	Heterogeneous nuclear ribonucleoprotein A1	0,8437	0,3885		1,59	0,0348	up
A0A480PH82	O43719	I3LC46	HTATSF1	HIV Tat-specific factor 1	1,0235	0,9369		1,93	0,0462	up
A0A4X1UGM9	Q9UPP2	F1SK81	IQSEC3	IQ motif and SEC7 domain-containing protein 3	1,243	0,2002		1,56	0,0188	up
A0A481B0D0	Q13683	A0A481B0D0	ITGA7	Integrin alpha-7	0,9156	0,7067		1,95	0,0135	up
A0A480TCT8	Q15046	F1S458	KARS1	Lysine--tRNA ligase	0,8462	0,3258		1,53	0,025	up

A0A287AT80	B7ZMF5	F1S2E7	KCNMA1	BK channel	1,3654	0,1344		1,72	0,019	up
A0A4X1UAA8	P32455	F1S4D7	LOC100523668	GB1/RHD3-type G domain-containing protein	0,9939	0,9719		1,66	0,0121	up
A0A5G2QZZ8	Q9BQ69	A0A5G2QZZ8	MACROD1	Mono-ADP ribosylhydrolase 1	0,8111	0,2927		1,6	0,0342	up
A0A287ANJ5	Q6DN14	I3LFJ9	MCTP1	Multiple C2 and transmembrane domain containing 1	1,2003	0,3666		1,59	0,0404	up
I3LRY9	Q9BUU2	I3LRY9	METTL22	Methyltransferase like 22	1,0606	0,7294		1,55	0,0244	up
A0A480W0I3	Q7RTP6	F1SHR1	MICAL3	F-actin monooxygenase	0,6923	0,179		1,89	0,0343	up
A0A287A0G6	Q96T76	F1S8Y2	MMS19	MMS19 nucleotide excision repair protein	1,5672	0,1112		2,2	0,0122	up
A0A4X1W511	Q7Z3U7	A0A4X1W511	MON2	Protein MON2 homolog	0,8177	0,3742		1,7	0,0349	up
A0A287BEB3	Q9P2K5	F1SN63	MYEF2	Myelin expression factor 2	0,9222	0,6606		1,6	0,0242	up
Q9G7T5	P03886	O79874	NADH1	NADH-ubiquinone oxidoreductase chain 1	1,0859	0,8044		2,16	0,0393	up
A0A287ARH5	P20929	F1SHX0	NEB	Nebulin	1,178	0,4182		1,79	0,0124	up
A0A4X1TN78	O76041	F1RVG6	NEBL	Nebulette	0,8781	0,3769		1,56	0,0089	up
A0A4X1TG56	P46934	I3L6R7	NEDD4	HECT-type E3 ubiquitin transferase	1,0992	0,5304		1,56	0,0111	up
A0A480H9T2	Q14982	A0A480IG74	OPCML	Opioid-binding protein/cell adhesion molecule isoform X1	0,9947	0,9771		1,68	0,0157	up

A0A287AEY8	Q9Y3D7	F1RK50	PAM16	Mitochondrial import inner membrane translocase subunit TIM16	1,1739	0,4176		1,59	0,0363	up
A0A480ECQ3	O60245	A0A480ECQ3	PCDH7	Protocadherin-7 isoform d	0,9573	0,7284		1,51	0,0056	up
Q29305	P13667	F1SAD9	PDIA4	Protein disulfide-isomerase	0,7985	0,2662		1,69	0,0207	up
A0A5G2QFU3	Q4KWH8	I3LFF0	PLCH1	Phosphoinositide phospholipase C	0,8485	0,4755		2,36	0,002	up
F1SUT1	Q9UF11	F1SUT1	PLEKHB1	Pleckstrin homology domain containing B1	1,1164	0,3817		1,54	0,0039	up
A0A286ZQN2	Q7Z5L7	I3LEB7	PODN	Podocan	0,7003	0,2354		1,99	0,0378	up
A0A286ZL42	P62136	Q2EHH8	PPP1CA	Serine/threonine-protein phosphatase PP1-alpha catalytic subunit	1,0426	0,7811		1,56	0,0112	up
F1RIY9	P31321	F1RIY9	PRKAR1B	Protein kinase cAMP-dependent type I regulatory subunit beta	1,3532	0,0861		1,52	0,0292	up
Q5GA59	P25786	F2Z5L7	PSMA1	Proteasome subunit alpha type 1 (Fragment)	1,0248	0,9234		1,84	0,034	up
G3DRF8	P28072	F1RFV5	PSMB6	Proteasome subunit beta type-6	0,7625	0,1948		1,64	0,0331	up
A0A480PKE7	P10586	A0A480PKE7	PTPRF	Receptor-type tyrosine-protein phosphatase F isoform 6	0,9272	0,6853		1,55	0,0356	up
A0A287BTC4	Q9NP72	I3LC07	RAB18	Ras-related protein Rab-18	1,021	0,9248		2,1	0,0047	up
A0A5G2QWM5	Q969Q5	F1S3C2	RAB24	RAB24, member RAS oncogene family	1,1859	0,3915		1,86	0,0073	up
A0A287B386	P18621	I3LT81	RPL17	60S ribosomal protein L17	0,7588	0,0556		1,56	0,0057	up

A0A287A0E2	Q9NR46	I3LUB3	SH3GLB2	SH3 domain containing GRB2 like, endophilin B2	0,8504	0,4728		1,64	0,0475	up
F1RR85	Q6P1M0	F1RR85	SLC27A4	Long-chain fatty acid transport protein 4	1,5937	0,0836		1,96	0,0226	up
A0A480JXS2	O60271	A0A480JXS2	SPAG9	C-Jun-amino-terminal kinase-interacting protein 4 isoform X1	1,0269	0,8754		1,58	0,0192	up
A4H2R5	P09486	P20112	SPARC	Osteonectin (Fragment)	1,2864	0,1175		1,5	0,0214	up
A0A5G2RJD9	Q9C093	Q2IA00	SPEF2	Sperm flagellar protein 2	0,6662	0,0509		1,58	0,0391	up
A0A4X1SJH8	Q07955	Q3YLA6	SRSF1	Serine/arginine-rich splicing factor 1	1,4697	0,0294		1,58	0,0163	up
A0A4X1UF36	Q8TCJ2	F1RRF2	STT3B	Dolichyl-diphosphooligosaccharide--protein glycotransferase	1,0838	0,7741		1,85	0,0498	up
A0A5G2QVT2	Q8WUH6	A0A5G2QVT2	TMEM263	Transmembrane protein 263	1,089	0,6656		1,6	0,0339	up
A0A287A351	Q9HBF4	F1S3L2	ZFYVE1	Zinc finger FYVE-type containing 1	0,9434	0,7187		1,56	0,0169	up
A0A286ZSN0	P25054	D4PEB7	APC	APC regulator of WNT signaling pathway	0,6316	0,017	down	1,75	0,0166	up
A0A4X1UH24	O14983	F1RFH9	ATP2A1	Calcium-transporting ATPase	0,5386	0,0461	down	1,98	0,0381	up
A0A5S6GGN8	Q9NQX7	Q06AV4	ITM2C	Integral membrane protein 2C	0,5231	0,0147	down	1,71	0,0139	up
A0A5G2QMV1	Q96L93	I3LEQ5	KIF16B	Kinesin-like protein KIF16B	0,5443	0,0352	down	1,52	0,0428	up
A0A287BBE4	Q9BQG0	F1RGP1	MYBBP1A	MYB binding protein 1a	0,6319	0,0389	down	1,71	0,0485	up

I3L829	Q8TAT6	I3L829	NPLOC4	NPL4 homolog, ubiquitin recognition factor	0,6424	0,027	down	1,85	0,0336	up
A0A4X1VQE8	Q99569	F1RPT5	PKP4	Plakophilin 4	0,574	0,0054	down	2,45	0,0081	up
A0A480TRN1	Q9UPN7	F1RMN3	PPP6R1	Protein phosphatase 6 regulatory subunit 1	0,5337	0,0253	down	1,58	0,0448	up
A0A5G2RBG1	P26599	F1S6R7	PTBP1	Polypyrimidine tract-binding protein 1	0,6407	0,0364	down	1,6	0,0377	up
A0A4X1SIX3	O95260	F1SEH8	ATE1	Arginyl-tRNA--protein transferase 1	1,7126	0,008	up	2,3	0,0057	up
A0A287A7Q1	O95319	F1RUN0	CELF2	CUGBP Elav-like family member 2	2,2374	0,014	up	1,62	0,0454	up
A0A4X1UX60	Q9H078	A0A4X1UX60	CLPB	Caseinolytic peptidase B protein homolog	1,7875	0,0097	up	1,6	0,04	up
K7GSF4	Q06787	K7GNF4	FMR1	Synaptic functional regulator FMR1	1,9833	0,0025	up	1,65	0,0254	up
A0A480M5N0	Q08378	F1RI72	GOLGA3	Golgin subfamily A member 3	1,5172	0,0401	up	1,69	0,0127	up
A0A4X1W5L4	P98160	F1SU03	HSPG2	Heparan sulfate proteoglycan 2	2,5647	0,0085	up	1,68	0,0267	up
A0A4X1TMN0	K7ERN1	F1SM37	NEDD4L	HECT-type E3 ubiquitin transferase	2,0233	0,0246	up	1,78	0,0179	up
A0A4X1TEY6	Q15382	F2Z5R2	RHEB	GTP-binding protein Rheb	1,8749	0,0135	up	1,96	0,0325	up
A0A287AN91	Q6P3W7	F1SQU2	SCYL2	SCY1 like pseudokinase 2	1,5003	0,0179	up	1,56	0,0023	up
A0A480V246	Q13596	I3LJ78	SNX1	Sorting nexin-1	1,8866	0,0276	up	1,73	0,0377	up

A0A480V246	Q13596	I3LJ78	SNX1	Sorting nexin-1	1,886<br>6	0,027<br>6	up	1,7<br>3	0,037<br>7	up

Table S5. Gene Ontology analysis of the response of NBW and LBW groups to an HFD.

Response of NBW and LBW animals to HFD: Comparison between GO categories in NBW and LBW groups: main categories from Panther have been selected and fold of enrichment indicated. Only positive (+)

Biological Process

NBW	Enrichment	LBW	Enrichment
negative regulation of synaptic vesicle exocytosis (GO:2000301)	69.81	negative regulation of mRNA splicing, via spliceosome (GO:0048025)	24.25
regulation of synaptic vesicle cycle (GO:0098693)	38.79	negative regulation of RNA splicing (GO:0033119)	19.76
regulation of neurotransmitter transport (GO:0051588)	7.83	negative regulation of mRNA processing (GO:0050686)	17.21
oxidative phosphorylation (GO:0006119)	7.21	regulation of alternative mRNA splicing, via spliceosome (GO:0000381)	16.01
regulation of synapse organization (GO:0050807)	5.54	regulation of mRNA splicing, via spliceosome (GO:0048024)	12.43
regulation of neurotransmitter levels (GO:0001505)	5.36	post-Golgi vesicle-mediated transport (GO:0006892)	9.90
regulation of synapse structure or activity (GO:0050803)	5.36	regulation of mRNA processing (GO:0050684)	9.21
synapse organization (GO:0050808)	5.23	regulation of RNA splicing (GO:0043484)	8.54
ATP metabolic process (GO:0046034)	5.18	Golgi vesicle transport (GO:0048193)	5.22
energy derivation by oxidation of organic compounds (GO:0015980)	4.75	regulation of mRNA metabolic process (GO:1903311)	4.77
regulation of vesicle-mediated transport (GO:0060627)	3.96	vesicle-mediated transport (GO:0016192)	2.81
intracellular protein transport (GO:0006886)	3.72	intracellular transport (GO:0046907)	2.40
cell junction organization (GO:0034330)	3.66	cellular protein localization (GO:0034613)	2.25
protein localization to membrane (GO:0072657)	3.50	cellular macromolecule localization (GO:0070727)	2.23
intracellular transport (GO:0046907)	3.17	establishment of localization in cell (GO:0051649)	2.11
protein transport (GO:0015031)	3.05	cellular localization (GO:0051641)	2.07
establishment of protein localization (GO:0045184)	3.02	regulation of molecular function (GO:0065009)	1.81
establishment of localization in cell (GO:0051649)	2.88	transport (GO:0006810)	1.71
cellular protein localization (GO:0034613)	2.71	establishment of localization (GO:0051234)	1.66
cellular macromolecule localization (GO:0070727)	2.69	localization (GO:0051179)	1.62
nitrogen compound transport (GO:0071705)	2.58	cellular process (GO:0009987)	1.22
central nervous system development (GO:0007417)	2.55	biological_process (GO:0008150)	1.13

cellular localization (GO:0051641)	2.47
organic substance transport (GO:0071702)	2.35
protein localization (GO:0008104)	2.33
macromolecule localization (GO:0033036)	2.27
transport (GO:0006810)	2.08
nervous system development (GO:0007399)	2.07
establishment of localization (GO:0051234)	2.02
cellular component assembly (GO:0022607)	2.02

Molecular Function

NBW	Enrichment	LBW	Enrichment
cadherin binding (GO:0045296)	3.96	pre-mRNA binding (GO:0036002)	16.42
cell adhesion molecule binding (GO:0050839)	3.68	structural constituent of cytoskeleton (GO:0005200)	7.18
cytoskeletal protein binding (GO:0008092)	2.90	cadherin binding (GO:0045296)	4.96
oxidoreductase activity (GO:0016491)	2.82	actin binding (GO:0003779)	4.48
anion binding (GO:0043168)	2.12	cell adhesion molecule binding (GO:0050839)	4.37
RNA binding (GO:0003723)	2.07	cytoskeletal protein binding (GO:0008092)	2.98
nucleotide binding (GO:0000166)	1.98	protein-containing complex binding (GO:0044877)	2.43
nucleoside phosphate binding (GO:1901265)	1.98	anion binding (GO:0043168)	1.90
small molecule binding (GO:0036094)	1.90	protein binding (GO:0005515)	1.21
catalytic activity (GO:0003824)	1.53	binding (GO:0005488)	1.16
protein binding (GO:0005515)	1.27	molecular_function (GO:0003674)	1.11
binding (GO:0005488)	1.18		
molecular_function (GO:0003674)	1.11		

Cellular Component

NBW	Enrich-ment	LBW	En-rich-ment
platelet alpha granule membrane (GO:0031092)	20.53	UFD1-NPL4 complex (GO:0036501)	> 100
cytochrome complex (GO:0070069)	11.35	H zone (GO:0031673)	> 100
lysosomal lumen (GO:0043202)	7.20	endoplasmic reticulum-Golgi intermediate compartment membrane (GO:0033116)	6.93
basement membrane (GO:0005604)	7.20	ruffle membrane (GO:0032587)	6.47
lipid droplet (GO:0005811)	7.20	exocytic vesicle (GO:0070382)	4.66
respirasome (GO:0070469)	7.05	postsynaptic density (GO:0014069)	4.46
Schaffer collateral - CA1 synapse (GO:0098685)	7.01	extrinsic component of membrane (GO:0019898)	4.43
respiratory chain complex (GO:0098803)	6.54	asymmetric synapse (GO:0032279)	4.39
mitochondrial respirasome (GO:0005746)	6.54	postsynaptic specialization (GO:0099572)	4.14
glutamatergic synapse (GO:0098978)	5.32	transport vesicle (GO:0030133)	4.09
postsynaptic density (GO:0014069)	4.86	neuron to neuron synapse (GO:0098984)	4.08
asymmetric synapse (GO:0032279)	4.79	synaptic vesicle (GO:0008021)	4.07
neuron to neuron synapse (GO:0098984)	4.77	sarcomere (GO:0030017)	4.03
postsynaptic specialization (GO:0099572)	4.51	cell cortex (GO:0005938)	3.65
postsynapse (GO:0098794)	4.00	postsynapse (GO:0098794)	3.33
secretory granule membrane (GO:0030667)	3.74	actin cytoskeleton (GO:0015629)	3.31
secretory granule lumen (GO:0034774)	3.62	cell leading edge (GO:0031252)	2.99
cytoplasmic vesicle lumen (GO:0060205)	3.59	synapse (GO:0045202)	2.77
vesicle lumen (GO:0031983)	3.57	cell junction (GO:0030054)	2.38
synapse (GO:0045202)	3.11	membrane protein complex (GO:0098796)	2.37
collagen-containing extracellular matrix (GO:0062023)	3.00	Golgi apparatus (GO:0005794)	2.27
secretory granule (GO:0030141)	2.95	extracellular vesicle (GO:1903561)	2.17
secretory vesicle (GO:0099503)	2.92	extracellular organelle (GO:0043230)	2.16
extracellular exosome (GO:0070062)	2.33	extracellular membrane-bounded organelle (GO:0065010)	2.16
extracellular vesicle (GO:1903561)	2.31	extracellular exosome (GO:0070062)	2.14
extracellular organelle (GO:0043230)	2.31	cell projection (GO:0042995)	2.03

extracellular membrane-bounded organelle (GO:0065010)	2.31
cell junction (GO:0030054)	2.27
membrane protein complex (GO:0098796)	2.05
cytoplasmic vesicle (GO:0031410)	2.04
intracellular vesicle (GO:0097708)	2.04
plasma membrane bounded cell projection (GO:0120025)	2.01
vesicle (GO:0031982)	1.98
cell projection (GO:0042995)	1.97
mitochondrion (GO:0005739)	1.90
cytosol (GO:0005829)	1.83
extracellular space (GO:0005615)	1.75
endomembrane system (GO:0012505)	1.70
organelle membrane (GO:0031090)	1.63
extracellular region (GO:0005576)	1.57
protein-containing complex (GO:0032991)	1.54
organelle lumen (GO:0043233)	1.53
intracellular organelle lumen (GO:0070013)	1.53
membrane-enclosed lumen (GO:0031974)	1.53
cytoplasm (GO:0005737)	1.52
membrane (GO:0016020)	1.35
membrane-bounded organelle (GO:0043227)	1.31
intracellular membrane-bounded organelle (GO:0043231)	1.31
organelle (GO:0043226)	1.29
intracellular anatomical structure (GO:0005622)	1.29
intracellular organelle (GO:0043229)	1.27
cellular anatomical entity (GO:0110165)	1.10
cellular_component (GO:0005575)	1.09

vesicle (GO:0031982)	2.01
neuron projection (GO:0043005)	1.99
plasma membrane bounded cell projection (GO:0120025)	1.99
cytoplasmic vesicle (GO:0031410)	1.96
intracellular vesicle (GO:0097708)	1.95
cytoskeleton (GO:0005856)	1.86
cytosol (GO:0005829)	1.78
endomembrane system (GO:0012505)	1.76
organelle membrane (GO:0031090)	1.58
cytoplasm (GO:0005737)	1.50
membrane (GO:0016020)	1.35
intracellular membrane-bounded organelle (GO:0043231)	1.31
membrane-bounded organelle (GO:0043227)	1.30
intracellular organelle (GO:0043229)	1.29
organelle (GO:0043226)	1.29
intracellular anatomical structure (GO:0005622)	1.29
cellular_component (GO:0005575)	1.08
cellular anatomical entity (GO:0110165)	1.08

Table S6. KEGG pathway analysis of the response of NBW and LBW groups to an HFD.

KEGG - NBW GROUP

METABOLISM AND SIGNAL TRANSDUCTION
• hsa01100 Metabolic pathways - Homo sapiens (human) (25)
hsa:10229 COQ7; coenzyme Q7, hydroxylase
hsa:10476 ATP5PD; ATP synthase peripheral stalk subunit d
hsa:1160 CKMT2; creatine kinase, mitochondrial 2
hsa:1340 COX6B1; cytochrome c oxidase subunit 6B1
hsa:1347 COX7A2; cytochrome c oxidase subunit 7A2
hsa:1355 COX15; cytochrome c oxidase assembly homolog COX15
hsa:201595 STT3B; STT3 oligosaccharyltransferase complex catalytic subunit B
hsa:204 AK2; adenylate kinase 2
hsa:220 ALDH1A3; aldehyde dehydrogenase 1 family member A3
hsa:23007 PLCH1; phospholipase C eta 1
hsa:2747 GLUD2; glutamate dehydrogenase 2
hsa:2799 GNS; glucosamine (N-acetyl)-6-sulfatase
hsa:2950 GSTP1; glutathione S-transferase pi 1
hsa:353 APRT; adenine phosphoribosyltransferase
hsa:410 ARSA; arylsulfatase A
hsa:4535 ND1; NADH dehydrogenase subunit 1
hsa:4597 MVD; mevalonate diphosphate decarboxylase
hsa:4725 NDUFS5; NADH:ubiquinone oxidoreductase subunit S5
hsa:51 ACOX1; acyl-CoA oxidase 1
hsa:5315 PKM; pyruvate kinase M1/2
hsa:7385 UQCRC2; ubiquinol-cytochrome c reductase core protein 2

KEGG - LBW GROUP

METABOLISM AND SIGNAL TRANSDUCTION
• hsa01100 Metabolic pathways - Homo sapiens (human) (31)
hsa:102724560 CBS; cystathionine beta-synthase like
hsa:10423 CDIPT; CDP-diacylglycerol--inositol 3-phosphatidyltransferase
hsa:10606 PAICS; phosphoribosylaminoimidazole carboxylase and phospho-ribosylaminoimidazolesuccinocarboxamide synthase
hsa:1607 DGKB; diacylglycerol kinase beta
hsa:2194 FASN; fatty acid synthase
hsa:23007 PLCH1; phospholipase C eta 1
hsa:23408 SIRT5; sirtuin 5
hsa:26007 TKFC; triokinase and FMN cyclase
hsa:2653 GCSH; glycine cleavage system protein H
hsa:2673 GFPT1; glutamine--fructose-6-phosphate transaminase 1
hsa:27034 ACAD8; acyl-CoA dehydrogenase family member 8
hsa:27430 MAT2B; methionine adenosyltransferase 2B
hsa:29926 GMPPA; GDP-mannose pyrophosphorylase A
hsa:3030 HADHA; hydroxyacyl-CoA dehydrogenase trifunctional multien-zyme complex subunit alpha
hsa:411 ARSB; arylsulfatase B
hsa:4535 ND1; NADH dehydrogenase subunit 1
hsa:4725 NDUFS5; NADH:ubiquinone oxidoreductase subunit S5
hsa:501 ALDH7A1; aldehyde dehydrogenase 7 family member A1
hsa:5142 PDE4B; phosphodiesterase 4B
hsa:51805 COQ3; coenzyme Q3, methyltransferase
hsa:5289 PIK3C3; phosphatidylinositol 3-kinase catalytic subunit type 3

hsa:762 CA4; carbonic anhydrase 4
hsa:8526 DGKE; diacylglycerol kinase epsilon
hsa:8540 AGPS; alkylglycerone phosphate synthase
hsa:883 KYAT1; kynurenine aminotransferase 1
• hsa04714 Thermogenesis - Homo sapiens (human) (10)
hsa:10476 ATP5PD; ATP synthase peripheral stalk subunit d
hsa:1340 COX6B1; cytochrome c oxidase subunit 6B1
hsa:1347 COX7A2; cytochrome c oxidase subunit 7A2
hsa:1355 COX15; cytochrome c oxidase assembly homolog COX15
hsa:4535 ND1; NADH dehydrogenase subunit 1
hsa:4725 NDUFS5; NADH:ubiquinone oxidoreductase subunit S5
hsa:55744 COA1; cytochrome c oxidase assembly factor 1
hsa:5606 MAP2K3; mitogen-activated protein kinase kinase 3
hsa:6009 RHEB; Ras homolog, mTORC1 binding
hsa:7385 UQCRC2; ubiquinol-cytochrome c reductase core protein 2
• hsa00190 Oxidative phosphorylation - Homo sapiens (human) (7)
hsa:10476 ATP5PD; ATP synthase peripheral stalk subunit d
hsa:1340 COX6B1; cytochrome c oxidase subunit 6B1
hsa:1347 COX7A2; cytochrome c oxidase subunit 7A2
hsa:1355 COX15; cytochrome c oxidase assembly homolog COX15
hsa:4535 ND1; NADH dehydrogenase subunit 1
hsa:4725 NDUFS5; NADH:ubiquinone oxidoreductase subunit S5
hsa:7385 UQCRC2; ubiquinol-cytochrome c reductase core protein 2
• hsa04910 Insulin signaling pathway - Homo sapiens (human) (6)
hsa:208 AKT2; AKT serine/threonine kinase 2

hsa:5372 PMM1; phosphomannomutase 1
hsa:54205 CYCS; cytochrome c, somatic
hsa:54963 UCKL1; uridine-cytidine kinase 1 like 1
hsa:5538 PPT1; palmitoyl-protein thioesterase 1
hsa:55862 ECHDC1; ethylmalonyl-CoA decarboxylase 1
hsa:56994 CHPT1; choline phosphotransferase 1
hsa:873 CBR1; carbonyl reductase 1
hsa:875 CBS; cystathionine beta-synthase
hsa:883 KYAT1; kynurenine aminotransferase 1
hsa:94005 PIGS; phosphatidylinositol glycan anchor biosynthesis class S
• hsa04714 Thermogenesis - Homo sapiens (human) (7)
hsa:4535 ND1; NADH dehydrogenase subunit 1
hsa:4725 NDUFS5; NADH:ubiquinone oxidoreductase subunit S5
hsa:55744 COA1; cytochrome c oxidase assembly factor 1
hsa:57521 RPTOR; regulatory associated protein of MTOR complex 1
hsa:6009 RHEB; Ras homolog, mTORC1 binding
hsa:6197 RPS6KA3; ribosomal protein S6 kinase A3
hsa:6599 SMARCC1; SWI/SNF related, matrix associated, actin dependent regulator of chromatin subfamily c member 1
• hsa04910 Insulin signaling pathway - Homo sapiens (human) (4)
hsa:2194 FASN; fatty acid synthase
hsa:5602 MAPK10; mitogen-activated protein kinase 10
hsa:57521 RPTOR; regulatory associated protein of MTOR complex 1
hsa:6009 RHEB; Ras homolog, mTORC1 binding
• hsa04024 cAMP signaling pathway - Homo sapiens (human) (5)
hsa:2893 GRIA4; glutamate ionotropic receptor AMPA type subunit 4

hsa:5499 PPP1CA; protein phosphatase 1 catalytic subunit alpha
hsa:5575 PRKAR1B; protein kinase cAMP-dependent type I regulatory subunit beta
hsa:5792 PTPRF; protein tyrosine phosphatase receptor type F
hsa:6009 RHEB; Ras homolog, mTORC1 binding
hsa:673 BRAF; B-Raf proto-oncogene, serine/threonine kinase
• hsa04931 Insulin resistance - Homo sapiens (human) (4)
hsa:10999 SLC27A4; solute carrier family 27 member 4
hsa:208 AKT2; AKT serine/threonine kinase 2
hsa:5499 PPP1CA; protein phosphatase 1 catalytic subunit alpha
hsa:5792 PTPRF; protein tyrosine phosphatase receptor type F
• hsa04024 cAMP signaling pathway - Homo sapiens (human) (6)
hsa:208 AKT2; AKT serine/threonine kinase 2
hsa:487 ATP2A1; ATPase sarcoplasmic/endoplasmic reticulum Ca2+ transporting 1
hsa:51 ACOX1; acyl-CoA oxidase 1
hsa:5499 PPP1CA; protein phosphatase 1 catalytic subunit alpha
hsa:5908 RAP1B; RAP1B, member of RAS oncogene family
hsa:673 BRAF; B-Raf proto-oncogene, serine/threonine kinase
• hsa04010 MAPK signaling pathway - Homo sapiens (human) (5)
hsa:208 AKT2; AKT serine/threonine kinase 2
hsa:4763 NF1; neurofibromin 1
hsa:5606 MAP2K3; mitogen-activated protein kinase kinase 3

hsa:4301 AFDN; afadin, adherens junction formation factor
hsa:487 ATP2A1; ATPase sarcoplasmic/endoplasmic reticulum Ca2+ transporting 1
hsa:5142 PDE4B; phosphodiesterase 4B
hsa:5602 MAPK10; mitogen-activated protein kinase 10
• hsa04020 Calcium signaling pathway - Homo sapiens (human) (4)
hsa:10105 PPIF; peptidylprolyl isomerase F
hsa:2769 GNA15; G protein subunit alpha 15
hsa:487 ATP2A1; ATPase sarcoplasmic/endoplasmic reticulum Ca2+ transporting 1
hsa:777 CACNA1E; calcium voltage-gated channel subunit alpha1 E
• hsa00260 Glycine, serine and threonine metabolism - Homo sapiens (human) (4)
hsa:102724560 CBS; cystathionine beta-synthase like
hsa:2653 GCSH; glycine cleavage system protein H
hsa:501 ALDH7A1; aldehyde dehydrogenase 7 family member A1
hsa:875 CBS; cystathionine beta-synthase
• hsa04150 mTOR signaling pathway - Homo sapiens (human) (4)
hsa:57521 RPTOR; regulatory associated protein of MTOR complex 1
hsa:6009 RHEB; Ras homolog, mTORC1 binding
hsa:6197 RPS6KA3; ribosomal protein S6 kinase A3
hsa:64798 DEPTOR; DEP domain containing MTOR interacting protein
• hsa00270 Cysteine and methionine metabolism - Homo sapiens (human) (4)

hsa:5908 RAP1B; RAP1B, member of RAS oncogene family
hsa:673 BRAF; B-Raf proto-oncogene, serine/threonine kinase
• hsa04022 cGMP-PKG signaling pathway - Homo sapiens (human) (4)
hsa:208 AKT2; AKT serine/threonine kinase 2
hsa:3708 ITPR1; inositol 1,4,5-trisphosphate receptor type 1
hsa:487 ATP2A1; ATPase sarcoplasmic/endoplasmic reticulum Ca2+ transporting 1
hsa:5499 PPP1CA; protein phosphatase 1 catalytic subunit alpha
• hsa04151 PI3K-Akt signaling pathway - Homo sapiens (human) (4)
hsa:1282 COL4A1; collagen type IV alpha 1 chain
hsa:208 AKT2; AKT serine/threonine kinase 2
hsa:3679 ITGA7; integrin subunit alpha 7
hsa:6009 RHEB; Ras homolog, mTORC1 binding
AUTOPHAGY AND SENESCENCE
• hsa04140 Autophagy - animal - Homo sapiens (human) (7)
hsa:208 AKT2; AKT serine/threonine kinase 2
hsa:23192 ATG4B; autophagy related 4B cysteine peptidase
hsa:3708 ITPR1; inositol 1,4,5-trisphosphate receptor type 1
hsa:3920 LAMP2; lysosomal associated membrane protein 2
hsa:53349 ZFYVE1; zinc finger FYVE-type containing 1
hsa:6009 RHEB; Ras homolog, mTORC1 binding
hsa:64422 ATG3; autophagy related 3
• hsa04218 Cellular senescence - Homo sapiens (human) (5)
hsa:208 AKT2; AKT serine/threonine kinase 2

hsa:102724560 CBS; cystathionine beta-synthase like
hsa:27430 MAT2B; methionine adenosyltransferase 2B
hsa:875 CBS; cystathionine beta-synthase
hsa:883 KYAT1; kynurenine aminotransferase 1
AUTOPHAGY AND SENESCENCE
• hsa04140 Autophagy - animal - Homo sapiens (human) (6)
hsa:30849 PIK3R4; phosphoinositide-3-kinase regulatory subunit 4
hsa:5289 PIK3C3; phosphatidylinositol 3-kinase catalytic subunit type 3
hsa:5602 MAPK10; mitogen-activated protein kinase 10
hsa:57521 RPTOR; regulatory associated protein of MTOR complex 1
hsa:6009 RHEB; Ras homolog, mTORC1 binding
hsa:64798 DEPTOR; DEP domain containing MTOR interacting protein
• hsa04210 Apoptosis - Homo sapiens (human) (4)
hsa:54205 CYCS; cytochrome c, somatic
hsa:5602 MAPK10; mitogen-activated protein kinase 10

hsa:3708 ITPR1; inositol 1,4,5-trisphosphate receptor type 1
hsa:5499 PPP1CA; protein phosphatase 1 catalytic subunit alpha
hsa:5606 MAP2K3; mitogen-activated protein kinase kinase 3
hsa:6009 RHEB; Ras homolog, mTORC1 binding
LYSOSOMES AND VESICULAR TRANSPORT
• hsa04142 Lysosome - Homo sapiens (human) (5)
hsa:10577 NPC2; NPC intracellular cholesterol transporter 2
hsa:2799 GNS; glucosamine (N-acetyl)-6-sulfatase
hsa:3920 LAMP2; lysosomal associated membrane protein 2
hsa:410 ARSA; arylsulfatase A
hsa:8546 AP3B1; adaptor related protein complex 3 subunit beta 1
• hsa04144 Endocytosis - Homo sapiens (human) (4)
hsa:440073 IQSEC3; IQ motif and Sec7 domain ArfGEF 3
hsa:4734 NEDD4; NEDD4 E3 ubiquitin protein ligase
hsa:56904 SH3GLB2; SH3 domain containing GRB2 like, endophilin B2
hsa:6642 SNX1; sorting nexin 1
• hsa04141 Protein processing in endoplasmic reticulum - Homo sapiens (human) (4)
hsa:11231 SEC63; SEC63 homolog, protein translocation regulator
hsa:201595 STT3B; STT3 oligosaccharyltransferase complex catalytic subunit B
hsa:55666 NPLOC4; NPL4 homolog, ubiquitin recognition factor
hsa:9601 PDIA4; protein disulfide isomerase family A member 4
• hsa04015 Rap1 signaling pathway - Homo sapiens (human) (4)
hsa:208 AKT2; AKT serine/threonine kinase 2

hsa:7846 TUBA1A; tubulin alpha 1a
hsa:84790 TUBA1C; tubulin alpha 1c
LYSOSOMES AND VESICULAR TRANSPORT
• hsa04142 Lysosome - Homo sapiens (human) (5)
hsa:1176 AP3S1; adaptor related protein complex 3 subunit sigma 1
hsa:2517 FUCA1; alpha-L-fucosidase 1
hsa:411 ARSB; arylsulfatase B
hsa:5538 PPT1; palmitoyl-protein thioesterase 1
hsa:6272 SORT1; sortilin 1
• hsa04144 Endocytosis - Homo sapiens (human) (4)
hsa:23096 IQSEC2; IQ motif and Sec7 domain ArfGEF 2
hsa:27243 CHMP2A; charged multivesicular body protein 2A
hsa:6642 SNX1; sorting nexin 1
hsa:832 CAPZB; capping actin protein of muscle Z-line subunit beta
• hsa04141 Protein processing in endoplasmic reticulum - Homo sapiens (human) (6)
hsa:10952 SEC61B; SEC61 translocon subunit beta
hsa:3300 DNAJB2; DnaJ heat shock protein family (Hsp40) member B2
hsa:55666 NPLOC4; NPL4 homolog, ubiquitin recognition factor
hsa:5602 MAPK10; mitogen-activated protein kinase 10
hsa:7095 SEC62; SEC62 homolog, preprotein translocation factor
hsa:7353 UFD1; ubiquitin recognition factor in ER associated degradation 1

hsa:5606 MAP2K3; mitogen-activated protein kinase kinase 3
hsa:5908 RAP1B; RAP1B, member of RAS oncogene family
hsa:673 BRAF; B-Raf proto-oncogene, serine/threonine kinase

NEUROLOGIC DISEASES

• hsa04723 Retrograde endocannabinoid signaling - Homo sapiens (human) (4)
hsa:2893 GRIA4; glutamate ionotropic receptor AMPA type subunit 4
hsa:4535 ND1; NADH dehydrogenase subunit 1
hsa:4725 NDUFS5; NADH:ubiquinone oxidoreductase subunit S5
hsa:5602 MAPK10; mitogen-activated protein kinase 10
hsa:5602 MAPK10; mitogen-activated protein kinase 10
hsa:5692 PSMB4; proteasome 20S subunit beta 4
hsa:7846 TUBA1A; tubulin alpha 1a
hsa:84790 TUBA1C; tubulin alpha 1c
• hsa04145 Phagosome - Homo sapiens (human) (4)
hsa:10952 SEC61B; SEC61 translocon subunit beta
hsa:5289 PIK3C3; phosphatidylinositol 3-kinase catalytic subunit type 3
hsa:7846 TUBA1A; tubulin alpha 1a
hsa:84790 TUBA1C; tubulin alpha 1c
TRANSLATION AND SPLICING
• hsa03040 Spliceosome - Homo sapiens (human) (7)
hsa:11338 U2AF2; U2 small nuclear RNA auxiliary factor 2
hsa:1655 DDX5; DEAD-box helicase 5
hsa:3190 HNRNPK; heterogeneous nuclear ribonucleoprotein K
hsa:6429 SRSF4; serine and arginine rich splicing factor 4
hsa:6431 SRSF6; serine and arginine rich splicing factor 6
hsa:6434 TRA2B; transformer 2 beta homolog
hsa:9939 RBM8A; RNA binding motif protein 8A
NEUROLOGIC DISEASES

• hsa05022 Pathways of neurodegeneration - multiple diseases - Homo sapiens (human) (17)
hsa:1020 CDK5; cyclin dependent kinase 5
hsa:10476 ATP5PD; ATP synthase peripheral stalk subunit d
hsa:10540 DCTN2; dynactin subunit 2
hsa:1340 COX6B1; cytochrome c oxidase subunit 6B1
hsa:1347 COX7A2; cytochrome c oxidase subunit 7A2
hsa:1639 DCTN1; dynactin subunit 1
hsa:324 APC; APC regulator of WNT signaling pathway
hsa:3708 ITPR1; inositol 1,4,5-trisphosphate receptor type 1
hsa:4535 ND1; NADH dehydrogenase subunit 1
hsa:4725 NDUFS5; NADH:ubiquinone oxidoreductase subunit S5
hsa:487 ATP2A1; ATPase sarcoplasmic/endoplasmic reticulum Ca2+ transporting 1
hsa:53349 ZFYVE1; zinc finger FYVE-type containing 1
hsa:5606 MAP2K3; mitogen-activated protein kinase kinase 3
hsa:5682 PSMA1; proteasome 20S subunit alpha 1
hsa:5694 PSMB6; proteasome 20S subunit beta 6
hsa:673 BRAF; B-Raf proto-oncogene, serine/threonine kinase
hsa:7385 UQCRC2; ubiquinol-cytochrome c reductase core protein 2
• hsa05016 Huntington disease - Homo sapiens (human) (11)
hsa:10476 ATP5PD; ATP synthase peripheral stalk subunit d
hsa:10540 DCTN2; dynactin subunit 2
hsa:1340 COX6B1; cytochrome c oxidase subunit 6B1
hsa:1347 COX7A2; cytochrome c oxidase subunit 7A2
hsa:1639 DCTN1; dynactin subunit 1
hsa:3708 ITPR1; inositol 1,4,5-trisphosphate receptor type 1

• hsa05016 Huntington disease - Homo sapiens (human) (14)
hsa:10105 PPIF; peptidylprolyl isomerase F
hsa:10120 ACTR1B; actin related protein 1B
hsa:11258 DCTN3; dynactin subunit 3
hsa:1639 DCTN1; dynactin subunit 1
hsa:2893 GRIA4; glutamate ionotropic receptor AMPA type subunit 4
hsa:30849 PIK3R4; phosphoinositide-3-kinase regulatory subunit 4
hsa:4535 ND1; NADH dehydrogenase subunit 1
hsa:4725 NDUFS5; NADH:ubiquinone oxidoreductase subunit S5
hsa:5289 PIK3C3; phosphatidylinositol 3-kinase catalytic subunit type 3
hsa:54205 CYCS; cytochrome c, somatic
hsa:5602 MAPK10; mitogen-activated protein kinase 10
hsa:5692 PSMB4; proteasome 20S subunit beta 4
hsa:7846 TUBA1A; tubulin alpha 1a
hsa:84790 TUBA1C; tubulin alpha 1c
• hsa05010 Alzheimer disease - Homo sapiens (human) (13)
hsa:10105 PPIF; peptidylprolyl isomerase F
hsa:10313 RTN3; reticulon 3
hsa:30849 PIK3R4; phosphoinositide-3-kinase regulatory subunit 4
hsa:324 APC; APC regulator of WNT signaling pathway
hsa:4535 ND1; NADH dehydrogenase subunit 1
hsa:4725 NDUFS5; NADH:ubiquinone oxidoreductase subunit S5
hsa:487 ATP2A1; ATPase sarcoplasmic/endoplasmic reticulum Ca2+ transporting 1
hsa:5289 PIK3C3; phosphatidylinositol 3-kinase catalytic subunit type 3
hsa:54205 CYCS; cytochrome c, somatic

hsa:4535 ND1; NADH dehydrogenase subunit 1
hsa:4725 NDUFS5; NADH:ubiquinone oxidoreductase subunit S5
hsa:5682 PSMA1; proteasome 20S subunit alpha 1
hsa:5694 PSMB6; proteasome 20S subunit beta 6
hsa:7385 UQCRC2; ubiquinol-cytochrome c reductase core protein 2
• hsa05010 Alzheimer disease - Homo sapiens (human) (14)
hsa:1020 CDK5; cyclin dependent kinase 5
hsa:10476 ATP5PD; ATP synthase peripheral stalk subunit d
hsa:1340 COX6B1; cytochrome c oxidase subunit 6B1
hsa:1347 COX7A2; cytochrome c oxidase subunit 7A2
hsa:208 AKT2; AKT serine/threonine kinase 2
hsa:324 APC; APC regulator of WNT signaling pathway
hsa:3708 ITPR1; inositol 1,4,5-trisphosphate receptor type 1
hsa:4535 ND1; NADH dehydrogenase subunit 1
hsa:4725 NDUFS5; NADH:ubiquinone oxidoreductase subunit S5
hsa:487 ATP2A1; ATPase sarcoplasmic/endoplasmic reticulum Ca2+ transporting 1
hsa:5682 PSMA1; proteasome 20S subunit alpha 1
hsa:5694 PSMB6; proteasome 20S subunit beta 6
hsa:673 BRAF; B-Raf proto-oncogene, serine/threonine kinase
hsa:7385 UQCRC2; ubiquinol-cytochrome c reductase core protein 2
• hsa05014 Amyotrophic lateral sclerosis - Homo sapiens (human) (13)
hsa:10476 ATP5PD; ATP synthase peripheral stalk subunit d
hsa:10540 DCTN2; dynactin subunit 2
hsa:1340 COX6B1; cytochrome c oxidase subunit 6B1

hsa:5602 MAPK10; mitogen-activated protein kinase 10
hsa:5692 PSMB4; proteasome 20S subunit beta 4
hsa:7846 TUBA1A; tubulin alpha 1a
hsa:84790 TUBA1C; tubulin alpha 1c
• hsa05014 Amyotrophic lateral sclerosis - Homo sapiens (human) (12)
hsa:10452 TOMM40; translocase of outer mitochondrial membrane 40
hsa:11258 DCTN3; dynactin subunit 3
hsa:1639 DCTN1; dynactin subunit 1
hsa:30849 PIK3R4; phosphoinositide-3-kinase regulatory subunit 4
hsa:4535 ND1; NADH dehydrogenase subunit 1
hsa:4725 NDUFS5; NADH:ubiquinone oxidoreductase subunit S5
hsa:5289 PIK3C3; phosphatidylinositol 3-kinase catalytic subunit type 3
hsa:54205 CYCS; cytochrome c, somatic
hsa:5692 PSMB4; proteasome 20S subunit beta 4
hsa:7846 TUBA1A; tubulin alpha 1a
hsa:84790 TUBA1C; tubulin alpha 1c
• hsa05020 Prion disease - Homo sapiens (human) (8)
hsa:10105 PPIF; peptidylprolyl isomerase F
hsa:4535 ND1; NADH dehydrogenase subunit 1
hsa:4725 NDUFS5; NADH:ubiquinone oxidoreductase subunit S5
hsa:54205 CYCS; cytochrome c, somatic
hsa:5602 MAPK10; mitogen-activated protein kinase 10
hsa:5692 PSMB4; proteasome 20S subunit beta 4
hsa:7846 TUBA1A; tubulin alpha 1a

hsa:1347 COX7A2; cytochrome c oxidase subunit 7A2
hsa:1639 DCTN1; dynactin subunit 1
hsa:3178 HNRNPA1; heterogeneous nuclear ribonucleoprotein A1
hsa:4535 ND1; NADH dehydrogenase subunit 1
hsa:4725 NDUFS5; NADH:ubiquinone oxidoreductase subunit S5
hsa:4928 NUP98; nucleoporin 98 and 96 precursor
hsa:5606 MAP2K3; mitogen-activated protein kinase kinase 3
hsa:5682 PSMA1; proteasome 20S subunit alpha 1
hsa:5694 PSMB6; proteasome 20S subunit beta 6
hsa:7385 UQCRC2; ubiquinol-cytochrome c reductase core protein 2
• hsa05020 Prion disease - Homo sapiens (human) (10)
hsa:10476 ATP5PD; ATP synthase peripheral stalk subunit d
hsa:10963 STIP1; stress induced phosphoprotein 1
hsa:1340 COX6B1; cytochrome c oxidase subunit 6B1
hsa:1347 COX7A2; cytochrome c oxidase subunit 7A2
hsa:3708 ITPR1; inositol 1,4,5-trisphosphate receptor type 1
hsa:4535 ND1; NADH dehydrogenase subunit 1
hsa:4725 NDUFS5; NADH:ubiquinone oxidoreductase subunit S5
hsa:5682 PSMA1; proteasome 20S subunit alpha 1
hsa:5694 PSMB6; proteasome 20S subunit beta 6
hsa:7385 UQCRC2; ubiquinol-cytochrome c reductase core protein 2
• hsa05012 Parkinson disease - Homo sapiens (human) (9)
hsa:10476 ATP5PD; ATP synthase peripheral stalk subunit d
hsa:1340 COX6B1; cytochrome c oxidase subunit 6B1
hsa:1347 COX7A2; cytochrome c oxidase subunit 7A2
hsa:3708 ITPR1; inositol 1,4,5-trisphosphate receptor type 1

hsa:84790 TUBA1C; tubulin alpha 1c
• hsa05012 Parkinson disease - Homo sapiens (human) (8)
hsa:10105 PPIF; peptidylprolyl isomerase F
hsa:4535 ND1; NADH dehydrogenase subunit 1
hsa:4725 NDUFS5; NADH:ubiquinone oxidoreductase subunit S5
hsa:54205 CYCS; cytochrome c, somatic
hsa:5602 MAPK10; mitogen-activated protein kinase 10
hsa:5692 PSMB4; proteasome 20S subunit beta 4
hsa:7846 TUBA1A; tubulin alpha 1a
hsa:84790 TUBA1C; tubulin alpha 1c
• hsa05017 Spinocerebellar ataxia - Homo sapiens (human) (7)
hsa:10105 PPIF; peptidylprolyl isomerase F
hsa:30849 PIK3R4; phosphoinositide-3-kinase regulatory subunit 4
hsa:487 ATP2A1; ATPase sarcoplasmic/endoplasmic reticulum Ca2+ transporting 1
hsa:5289 PIK3C3; phosphatidylinositol 3-kinase catalytic subunit type 3
hsa:54205 CYCS; cytochrome c, somatic
hsa:5602 MAPK10; mitogen-activated protein kinase 10
hsa:5692 PSMB4; proteasome 20S subunit beta 4

hsa:4535 ND1; NADH dehydrogenase subunit 1
hsa:4725 NDUFS5; NADH:ubiquinone oxidoreductase subunit S5
hsa:5682 PSMA1; proteasome 20S subunit alpha 1
hsa:5694 PSMB6; proteasome 20S subunit beta 6
hsa:7385 UQCRC2; ubiquinol-cytochrome c reductase core protein 2
• hsa05208 Chemical carcinogenesis - reactive oxygen species - Homo sapiens (human) (8)
hsa:10476 ATP5PD; ATP synthase peripheral stalk subunit d
hsa:1340 COX6B1; cytochrome c oxidase subunit 6B1
hsa:1347 COX7A2; cytochrome c oxidase subunit 7A2
hsa:208 AKT2; AKT serine/threonine kinase 2
hsa:4535 ND1; NADH dehydrogenase subunit 1
hsa:4725 NDUFS5; NADH:ubiquinone oxidoreductase subunit S5
hsa:673 BRAF; B-Raf proto-oncogene, serine/threonine kinase
hsa:7385 UQCRC2; ubiquinol-cytochrome c reductase core protein 2
ECM, FOCAL ADHESION AND CYTOSKELETON
• hsa04810 Regulation of actin cytoskeleton - Homo sapiens (human) (4)
hsa:324 APC; APC regulator of WNT signaling pathway
hsa:3679 ITGA7; integrin subunit alpha 7
hsa:5499 PPP1CA; protein phosphatase 1 catalytic subunit alpha
hsa:673 BRAF; B-Raf proto-oncogene, serine/threonine kinase
• hsa04512 ECM-receptor interaction - Homo sapiens (human) (4)
hsa:1282 COL4A1; collagen type IV alpha 1 chain
hsa:3339 HSPG2; heparan sulfate proteoglycan 2
hsa:3679 ITGA7; integrin subunit alpha 7

• hsa05208 Chemical carcinogenesis - reactive oxygen species - Homo sapiens (human) (5)
hsa:10105 PPIF; peptidylprolyl isomerase F
hsa:4535 ND1; NADH dehydrogenase subunit 1
hsa:4725 NDUFS5; NADH:ubiquinone oxidoreductase subunit S5
hsa:5602 MAPK10; mitogen-activated protein kinase 10
hsa:873 CBR1; carbonyl reductase 1
ECM, FOCAL ADHESION AND CYTOSKELETON
• hsa04810 Regulation of actin cytoskeleton - Homo sapiens (human) (4)
hsa:1729 DIAPH1; diaphanous related formin 1
hsa:324 APC; APC regulator of WNT signaling pathway
hsa:3696 ITGB8; integrin subunit beta 8
hsa:85477 SCIN; scinderin
• hsa04530 Tight junction - Homo sapiens (human) (4)
hsa:4301 AFDN; afadin, adherens junction formation factor
hsa:5602 MAPK10; mitogen-activated protein kinase 10
hsa:7846 TUBA1A; tubulin alpha 1a

hsa:375790 AGRN; agrin
• hsa04510 Focal adhesion - Homo sapiens (human) (7)
hsa:1282 COL4A1; collagen type IV alpha 1 chain
hsa:208 AKT2; AKT serine/threonine kinase 2
hsa:29780 PARVB; parvin beta
hsa:3679 ITGA7; integrin subunit alpha 7
hsa:5499 PPP1CA; protein phosphatase 1 catalytic subunit alpha
hsa:5908 RAP1B; RAP1B, member of RAS oncogene family
hsa:673 BRAF; B-Raf proto-oncogene, serine/threonine kinase
LONG TERM POTENTIATION
• hsa04720 Long-term potentiation - Homo sapiens (human) (4)
hsa:3708 ITPR1; inositol 1,4,5-trisphosphate receptor type 1
hsa:5499 PPP1CA; protein phosphatase 1 catalytic subunit alpha
hsa:5908 RAP1B; RAP1B, member of RAS oncogene family
hsa:673 BRAF; B-Raf proto-oncogene, serine/threonine kinase
• hsa03013 Nucleocytoplasmic transport - Homo sapiens (human) (4)
hsa:23534 TNPO3; transportin 3
hsa:30000 TNPO2; transportin 2
hsa:4928 NUP98; nucleoporin 98 and 96 precursor
hsa:6613 SUMO2; small ubiquitin like modifier 2
**PLATELET ACTIVATION**
• hsa04611 Platelet activation - Homo sapiens (human) (6)
hsa:208 AKT2; AKT serine/threonine kinase 2
hsa:2243 FGA; fibrinogen alpha chain
hsa:3708 ITPR1; inositol 1,4,5-trisphosphate receptor type 1

hsa:84790 TUBA1C; tubulin alpha 1c
• hsa04510 Focal adhesion - Homo sapiens (human) (4)
hsa:1729 DIAPH1; diaphanous related formin 1
hsa:3696 ITGB8; integrin subunit beta 8
hsa:55742 PARVA; parvin alpha
hsa:5602 MAPK10; mitogen-activated protein kinase 10

hsa:5499 PPP1CA; protein phosphatase 1 catalytic subunit alpha
hsa:5908 RAP1B; RAP1B, member of RAS oncogene family
hsa:8773 SNAP23; synaptosome associated protein 23

Table S7. List of common and different proteins in NBW and LBW groups in response to an HFD.

Dark blue indicates common proteins; Light blue indicates common isoforms.

**NBW**	**LBW**	
ACAD10	ACAD8	AcylCoA dehydrogenase. ACAD10 is only active with R- and S-2-methyl-C15-CoA (uniprot). ACAD10 is Isobutyryl-CoA dehydrogenase and participates in valine catabolism
ACOX1	ACTR1B	
AGPS	ADAM9	
	ADD3	
	AFDN	
AGRN	AGRN	Agrin: isoform 2 is transmembrane form that is the predominate form in neurons of the brain, induces dendritic filopodia and synapse formation in mature hippocampal neurons in large part due to the attached glycosaminoglycan chains and the action of Rho-family GTPases. Isoform 1 is at the neuromuscular junction
AGRN	AIMP1	
AK2	AKAP9	
AKT2	AKAP9	
ALDH1A3	ALDH7A1	Aldehyde dehydrogenase 1: Cytosolic dehydrogenase that catalyzes the irreversible oxidation of a wide range of aldehydes to their corresponding carboxylic acid, important for retinoic acid. ALDH7: Oxidizes medium and long chain saturated and unsaturated aldehydes
	ANKS1B	
	ANXA13	

AP3B1	AP3S1	AP3B1: AP-3 complex subunit beta-1. Subunit of non-clathrin- and clathrin-associated adaptor protein complex 3 (AP-3) that plays a role in protein sorting in the late-Golgi/trans-Golgi network (TGN) and/or endosomes. In concert with the BLOC-1 complex, AP-3 is required to target cargos into vesicles assembled at cell bodies for delivery into neurites and nerve terminals.
		AP3S1: AP-3 complex subunit sigma-1. Part of the AP-3 complex, an adaptor-related complex which is not clathrin-associated. The complex is associated with the Golgi region as well as more peripheral structures. In concert with the BLOC-1 complex, AP-3 is required to target cargos into vesicles assembled at cell bodies for delivery into neurites and nerve terminals.
APC	APC	Adenomatous polyposis coli protein. Promotes rapid degradation of CTNNB1 and participates in Wnt signaling as a negative regulator. It is required for the localization of MACF1 to the cell membrane and this localization of MACF1 is critical for its function in microtubule stabilization
APPL2	APLP2	
APRT		
ARHGAP44		
ARHGEF9	ARHGEF9	Rho guanine nucleotide exchange factor 9
ARSA	ARSB	Arylsulfatase: Hydrolyzes cerebroside sulfate
	ARSD	
ATE1	ATE1	Arginyl-tRNA--protein transferase 1: Involved in the post-translational conjugation of arginine to the N-terminal aspartate or glutamate of a protein
ATG3	ATP13A1	
ATG4B		
ATP2A1	ATP2A1	Sarcoplasmic/endoplasmic reticulum calcium ATPase 1: major Ca2+ ATPase responsible for the reuptake of cytosolic Ca2+ into the sarcoplasmic reticulum
	ATP2A1	
ATP5PD	ATPAF1	ATP5PD: ATP synthase subunit d, mitochondrial
BAG3		ATPAF1: ATP synthase mitochondrial F1 complex assembly factor 1
BCLAF1		
BRAF		
C10H10orf67	C14H1orf198	No identified orfs

C2CD5	C16orf70	
CA4	CACNA1E	
CAMK1	CALCR	
CASK	CAPZB	
CAST	CARS2	
CCP110	CBR1	
CDK5	CBS	
	CC2D1A	
	CCDC88A	
	CCDC93	
	CDIPT	
	CDK16	
CELF2	CELF2	CUGBP Elav-like family member 2. RNA-binding protein implicated in the regulation of several post-transcriptional events. Involved in pre-mRNA alternative splicing, mRNA translation and stability
CELSR2	CEP170	
CFB	CHMP2A	
CKMT2	CHPT1	
	CLCC1	
CLPB	CLPB	Chaperone protein ClpB (also caseinolytix peptidase B protein homolog). May function as a regulatory ATPase and be related to secretion/protein trafficking process
	CLPTM1	
COA1	COA1	Cytochrome c oxidase assembly factor 1 homolog: Component of the MITRAC (mitochondrial translation regulation assembly intermediate of cytochrome c oxidase complex) complex, that regulates cytochrome c oxidase assembly. Required for assembly of mitochondrial respiratory chain complex I and complex IV.
COL4A1	COPS7B	COP9 signalosome complex subunit 7b
COPS8	COPS8	COP9 signalosome complex subunit 8: Component of the COP9 signalosome complex (CSN), a complex involved in various cellular and developmental processes. The CSN complex is an essential regulator of the ubiquitin (Ubl) conjugation pathway
COPZ1		

COQ7	COQ3	COQ7: 5-demethoxyubiquinone hydroxylase, mitochondrial. Catalyzes the hydroxylation of 2-polyprenyl-3-methyl-6-methoxy-1,4-benzoquinol (DMQH2) during ubiquinone biosynthesis. Has also a structural role in the COQ enzyme complex
COX15		COQ3: Ubiquinone biosynthesis O-methyltransferase, mitochondrial. O-methyltransferase that catalyzes the 2 O-methylation steps in the ubiquinone biosynthetic pathway
COX6B1		
COX7A2		
CRYZ	CRYZL1	CRYZ: Quinone oxidoreductase; Orthoquinones, such as 1,2-naphthoquinone or 9,10-phenanthrenequinone, are the best substrates (in vitro). May act in the detoxification of xenobiotics. Interacts with (AU)-rich elements (ARE) in the 3'-UTR of target mRNA species. Enhances the stability of mRNA coding for BCL2
CSTB		CRYZL1: Quinone oxidoreductase-like protein 1
CTTNBP2	CTNND2	CTTNBP2: CTTNBP2 N-terminal-like protein. Regulates lamellipodial actin dynamics in a CTTN-dependent manner. CTTN: Src substrate cortactin: hrough its interaction with CTTNBP2, involved in the regulation of neuronal spine density
DAAM2		CTNND2: Catenin delta-2. Has a critical role in neuronal development, particularly in the formation and/or maintenance of dendritic spines and synapses
	CYCS	
DCTN1	DCTN1	DCTN1: Dynactin subunit 1. Plays a key role in dynein-mediated retrograde transport of vesicles and organelles along microtubules by recruiting and tethering dynein to microtubules. Can regulate microtubule stability by promoting microtubule formation, nucleation and polymerization and by inhibiting microtubule catastrophe in neurons. Inhibits microtubule catastrophe by binding both to microtubules and to tubulin, leading to enhanced microtubule stability along the axon
DCTN2	DCTN3	DCTN2, DCTN3: Dynactin subunits 2 and 3
DDX17	DDX5	DDX17: Probable ATP-dependent RNA helicase DDX17. As an RNA helicase, unwinds RNA and alters RNA structures through ATP binding and hydrolysis. Involved in multiple cellular processes, including pre-mRNA splicing, alternative splicing, ribosomal RNA processing and miRNA processing, as well as transcription regulation. Regulates the alternative splicing of exons exhibiting specific features

	DDX58	DDX5: Probable ATP-dependent RNA helicase DDX5. Involved in the alternative regulation of pre-mRNA splicing. its RNA helicase activity is necessary for increasing tau exon 10 inclusion and occurs in a RBM4-dependent manner.
	DEPTOR	
DGKE	DGKB	DGKE: Diacylglycerol kinase epsilon. Membrane-bound diacylglycerol kinase that converts diacylglycerol/DAG into phosphatidic acid/phosphatidate/PA and regulates the respective levels of these two bioactive lipids
		DGKB: Diacylglycerol kinase beta. Diacylglycerol kinase that converts diacylglycerol/DAG into phosphatidic acid/phosphatidate/PA and regulates the respective levels of these two bioactive lipids. Specifically expressed in brain, it regulates neuron-specific morphological changes including neurite branching and neurite spine formation
	DIAPH1	
	DKK3	
DNAJA4	DNAJB2	DnaJ homolog subfamily A member 4
		DnaJ homolog subfamily B member 2: Functions as a co-chaperone, regulating the substrate binding and activating the ATPase activity of chaperones of the HSP70/heat shock protein 70 family
DOCK8	DOCK10	DOCK8: Dedicator of cytokinesis protein 8: Guanine nucleotide exchange factor (GEF) which specifically activates small GTPase CDC42 by exchanging bound GDP for free GTP
DPP8	DOCK9	DOCK9: Dedicator of cytokinesis protein 9. Guanine nucleotide-exchange factor (GEF) that activates CDC42 by exchanging bound GDP for free GTP. Overexpression induces filopodia formation
ELP1		DOCK10: Dedicator of cytokinesis protein 10. Guanine nucleotide-exchange factor (GEF) that activates CDC42 and RAC1 by exchanging bound GDP for free GTP. Essential for dendritic spine morphogenesis in Purkinje cells and in hippocampal neurons, via a CDC42-mediated pathway
	DST	
	ECHDC1	
	EML1	
	ENAH	
ERBIN	ERBIN	Acts as an adapter for the receptor ERBB2, in epithelia. Invbolved in cytoskeleton organization and regulation od postsynaptic membrane neurotransmitter receptor levels
ERMP1	ERH	

EXOC5	EXOC5	Exocyst complex component 5: Component of the exocyst complex involved in the docking of exocytic vesicles with fusion sites on the plasma membrane.
	EXOC6B	
FAM120B	FAM169A	FAM120B: Constitutive coactivator of peroxisome proliferator-activated receptor gamma. Functions as a transactivator of PPARG and ESR1. Functions in adipogenesis through PPARG activation
FARP1	FASN	FAM169A: Soluble lamin-associated protein of 75 kDa
FATP4	FKBP15	
FBXL18		
FBXO21		
FGA		
FLII		
FMR1	FMR1	Synaptic functional regulator FMR1: Multifunctional polyribosome-associated RNA-binding protein that plays a central role in neuronal development and synaptic plasticity through the regulation of alternative mRNA splicing, mRNA stability, mRNA dendritic transport and postsynaptic local protein synthesis of a subset of mRNAs
FSIP2	FSD1L	
FTH1	FUCA1	
GLUD1	GCSH	
GNS	GFM2	
	GFPT1	
	GLRX	
	GMPPA	
	GNA15	
GOLGA2	GOLGA2	Golgin subfamily A member 2: Peripheral membrane component of the cis-Golgi stack that acts as a membrane skeleton that maintains the structure of the Golgi apparatus, and as a vesicle thether that facilitates vesicle fusion to the Golgi membrane
GOLGA3	GOLGA3	Golgin subfamily A member 3: probably involved in maintaining Golgi structure.
GSTP1	GOSR2	
	GRIA4	
	HADHA	

HAPLN4	HAPLN4	Hyaluronan and proteoglycan link protein 4: Binds to hyaluronic acid and may be involved in formation of the extracellular matrix. Involved in central nervous system development
HMGB2		
HNRNPA1	HNRNPK	HNRNPA1: Heterogeneous nuclear ribonucleoprotein A1. Involved in the packaging of pre-mRNA into hnRNP particles, transport of poly(A) mRNA from the nucleus to the cytoplasm and may modulate splice site selection
HSD17B13		HNRNPK: Heterogeneous nuclear ribonucleoprotein K. One of the major pre-mRNA-binding proteins. Binds tenaciously to poly(C) sequences. Likely to play a role in the nuclear metabolism of hnRNAs, particularly for pre-mRNAs that contain cytidine-rich sequences
HSPG2	HSPG2	Basement membrane-specific heparan sulfate proteoglycan core protein: Integral component of basement membranes
HTATSF1		
IL17RE		
IQSEC3	IQSEC2	IQ motif and SEC7 domain-containing protein 3: Acts as a guanine nucleotide exchange factor (GEF) for ARF1.
		IQ motif and SEC7 domain-containing protein 2: Is a guanine nucleotide exchange factor for the ARF GTP-binding proteins.
ITGA7	ITGB8	ITGA7: Integrin alpha-7. Acts as Schwann cell receptor for laminin-2.
		ITGB8: Integrin beta-8. is a receptor for fibronectin
ITM2C	ITM2C	Integral membrane protein 2C: Negative regulator of amyloid-beta peptide production. May play a role in TNF-induced cell death and neuronal differentiation
ITPR1		
KARS1	JPH3	
KATNB1	KHDRBS2	
KCNMA1	KHDRBS3	
KIF16B	KIF16B	Kinesin-like protein KIF16B: Plus end-directed microtubule-dependent motor protein involved in endosome transport and receptor recycling and degradation
KYAT1	KYAT1	Kynurenine--oxoglutarate transaminase 1. Catalyzes the irreversible transamination of the L-tryptophan metabolite L-kynurenine to form kynurenic acid (KA), an intermediate in the tryptophan catabolic pathway which is also a broad spectrum antagonist of the three ionotropic excitatory amino acid receptors among others

LAMP2	LCMT1	
LGALS8	LGALS8	Galectin-8: Beta-galactoside-binding lectin that acts as a sensor of membrane damage
	LIMCH1	
LIN7C	LIN7C	Protein lin-7 homolog C: The tripartite complex composed of LIN7 (LIN7A, LIN7B or LIN7C), CASK and APBA1 associates with the motor protein KIF17 to transport vesicles containing N-methyl-D-aspartate (NMDA) receptor subunit NR2B along microtubules. This complex may have the potential to couple synaptic vesicle exocytosis to cell adhesion in brain
	LMBRD1	
LOC100523668	LOC100523668	GB1/RHD3-type G domain-containing protein. GTP binding
MACROD1	LRRC7	
MAP2K3	MAPK10	
MCTP1	MAT2B	
METAP2	MT2A	
METTL22	MTMR9	
METTL7A	MTSS2	
MICAL3		
MMS19		
MON2		
MVD		
MX2		
MYBBP1A	MYBBP1A	Myb-binding protein 1A. May activate or repress transcription via interactions with sequence specific DNA-binding proteins. Has a role in rRNA biogenesis together with PWP1
MYEF2		
NADH1	NADH1	NADH-ubiquinone oxidoreductase chain 1. Core subunit of the mitochondrial membrane respiratory chain NADH dehydrogenase (Complex I) which catalyzes electron transfer from NADH through the respiratory chain, using ubiquinone as an electron acceptor
NDE1		

NDUFS5	NDUFS5	NADH dehydrogenase [ubiquinone] iron-sulfur protein 5. Accessory subunit of the mitochondrial membrane respiratory chain NADH dehydrogenase (Complex I), that is believed not to be involved in catalysis. Complex I functions in the transfer of electrons from NADH to the respiratory chain. The immediate electron acceptor for the enzyme is believed to be ubiquinone.
NEB		
NEBL		
NEDD4		
NEDD4L	NEDD4L	E3 ubiquitin-protein ligase NEDD4-like: E3 ubiquitin-protein ligase which accepts ubiquitin from an E2 ubiquitin-conjugating enzyme in the form of a thioester and then directly transfers the ubiquitin to targeted substrates. romotes ubiquitination and internalization of various plasma membrane channels. Involved in the regulation of TOR signaling
NF1		
NLGN4X		
NPC2		
NPLOC4	NPLOC4	Nuclear protein localization protein 4 homolog. The ternary complex containing UFD1, VCP and NPLOC4 binds ubiquitinated proteins and is necessary for the export of misfolded proteins from the ER to the cytoplasm, where they are degraded by the proteasome
NRXN1		
NUP98		
OPCML	OCIAD2	
PAM16	PAICS	
PARVB	PARVA	PARVA: Alpha-parvin. Plays a role in the reorganization of the actin cytoskeleton, formation of lamellipodia and ciliogenesis. Plays a role in the establishment of cell polarity, cell adhesion, cell spreading, and directed cell migration.
PCDH7		PARVB: Beta-parvin. Adapter protein that plays a role in integrin signaling via ILK and in activation of the GTPases CDC42 and RAC1 by guanine exchange factors. Plays a role in cell adhesion, cell spreading, establishment or maintenance of cell polarity, and cell migration.
PDIA4	PDCD5	
PDXDC1	PDE4B	
PIN1	PDK2	
PKM	PDLIM5	
	PDXDC1	

	PIGS	
	PIK3C3	
	PIK3R4	
PKP4	PKP4	Plakophilin-4: Plays a role as a regulator of Rho activity during cytokinesis. May play a role in junctional plaques.
PLCH1	PLCH1	1-phosphatidylinositol 4,5-bisphosphate phosphodiesterase eta-1. The production of the second messenger molecules diacylglycerol (DAG) and inositol 1,4,5-trisphosphate (IP3) is mediated by calcium-activated phosphatidylinositol-specific phospholipase C enzymes
PLEKHB1	PLS1	
	PMM1	
PODN	PODN	Podocan: Negatively regulates cell proliferation and cell migration.
PPP1CA	PPIF	
PPP6R1	PPP6R1	Serine/threonine-protein phosphatase 6 regulatory subunit 1. Regulatory subunit of protein phosphatase 6 (PP6). May function as a scaffolding PP6 subunit. Involved in the PP6-mediated dephosphorylation of NFKBIE opposing its degradation in response to TNF-alpha
PRKAR1B	PPT1	
PRPSAP2	PRUNE2	
PSMA1	PSMB4	Proteasome subunit alpha 1: Component of the proteasome core, a large protease complex with broad specificity involved in protein degradation.
PSMB6		Proteasome subunit beta type-4: Non-catalytic component of the 20S core proteasome complex involved in the proteolytic degradation of most intracellular proteins
PTBP1	PTBP1	Proteasome subunit beta type-6
PTPRF	PTK2	
RAB24	RABGAP1L	RABGAP1L: Rab GTPase-activating protein 1-like. Plays a role in endocytosis and intracellular protein transport.
RAB3B	RABL6	RAB24: Ras-related protein Rab-24. May be involved in autophagy-related processes.
RAP1B	RBM8A	RABL6: Rab-like protein 6. May enhance cellular proliferation
RCSD1		RAB3B: Ras-related protein Rab-3B. Protein transport. Probably involved in vesicular traffic
		RAP1B: Ras-related protein Rap-1b. GTP-binding protein that possesses intrinsic GTPase activity
RHEB	RHEB	GTP-binding protein Rheb. Activates the protein kinase activity of mTORC1, and thereby plays a role in the regulation of apoptosis. Stimulates the phosphorylation of S6K1 and EIF4EBP1 through activation of mTORC1 signaling

RPL17	RNF123	
RPS18	RPS6KA3	
RPS19	RPTOR	
SAG	RSU1	
	RTN3	
	RTN4IP1	
	S100A2	
	SAE1	
SCCPDH	SCCPDH	Saccharopine dehydrogenase-like oxidoreductase. glycolipid biosynthetic process
	SCIN	
SCN9A	SCN8A	SCN9A: Sodium channel protein type 9 subunit alpha. Mediates the voltage-dependent sodium ion permeability of excitable membranes.
	SCRN3	SCN8A: Sodium channel protein type 8 subunit alpha. Mediates the voltage-dependent sodium ion permeability of excitable membranes
SCYL2	SCYL2	SCY1-like protein 2. Component of the AP2-containing clathrin coat that may regulate clathrin-dependent trafficking at plasma membrane, TGN and endosomal system. By regulating the expression of excitatory receptors at synapses, plays an essential role in neuronal function and signaling and in brain development
SEC63	SEC61B	SEC63: Translocation protein SEC63. Mediates cotranslational and post-translational transport of certain precursor polypeptides across endoplasmic reticulum
SH3GLB2	SEC62	SEC61B: Protein transport protein Sec61 subunit beta. Component of SEC61 channel-forming translocon complex that mediates transport of signal peptide-containing precursor polypeptides across the endoplasmic reticulum
		SEC62: Translocation protein SEC62. Mediates post-translational transport of precursor polypeptides across endoplasmic reticulum
	SERPINA1	
	SETD3	
	SIRT5	
SLA-2	SLA-1	SLA: Sus scrofa HLA family
SLA-3	SLA-2	
SLA-6	SLA-3	
SLA-B	SLA-6	

SLC27A4	SLC7A10	SLC27A4: Long-chain fatty acid transport protein 4. Involved in translocation of long-chain fatty acids (LFCA) across the plasma membrane
SLC4A7		SLC7A10: Asc-type amino acid transporter 1. Sodium-independent, high affinity transport of small neutral D- and L-amino acids. May play a role in the modulation of glutamatergic transmission through mobilization of D-serine at the glutamatergic synapse
SNAP23	SMARCC1	SLC4A7: Sodium bicarbonate cotransporter 3. Electroneutral sodium- and bicarbonate-dependent cotransporter
	SMYD5	
SNX1	SNX1	Sorting nexin-1. Involved in several stages of intracellular trafficking. Interacts with membranes containing phosphatidylinositol 3-phosphate (PtdIns(3P)) or phosphatidylinositol 3,5-bisphosphate
SPAG9	SORL1	
SPARC	SORT1	
SPARCL1		
SPEF2		
SRSF1	SRSF4	SRSF1: Serine/arginine-rich splicing factor 1. Plays a role in preventing exon skipping, ensuring the accuracy of splicing and regulating alternative splicing
STIP1	SRSF6	SRSF4: Serine/arginine-rich splicing factor 4. Plays a role in alternative splice site selection during pre-mRNA splicing
STRBP		SRSF6: Serine/arginine-rich splicing factor 6. Plays a role in constitutive splicing and modulates the selection of alternative splice sites. Plays a role in the alternative splicing of MAPT/Tau exon 10
STT3B	SUGP2	
SUGP2		
SUMO2		
SYNE1	SYN3	SYNE1: Nesprin-1. Multi-isomeric modular protein which forms a linking network between organelles and the actin cytoskeleton to maintain the subcellular spatial organization.
		SYN3: Synapsin-3. May be involved in the regulation of neurotransmitter release and synaptogenesis.
SYT3	SYT3	Synaptotagmin-3. Ca2+ sensor involved in Ca2+-dependent exocytosis of secretory vesicles through Ca2+ and phospholipid binding to the C2 domain.
	TKFC	

	TMED9	
TMEM263	TMEM245	TMEM263: Transmembrane protein 263. No information
		TMEM245: Transmembrane protein 245. No information
TNPO2	TMPRSS11A	
TNPO3	TMPRSS13	
	TMX4	
	TOMM40	
	TPM1	
	TPRG1L	
	TRA2B	
	TRAPPC9	
	TRIM9	
	TRIP11	
	TSC22D3	
	TUBA1A	
	TUBA1C	
	TXNRD2	
UQCRC2	U2AF2	
	UCKL1	
	UFD1	
	UTRN	
	VPS52	
	WDR13	WD repeat-containing protein 13: no information
WDR45	WDR45	WD repeat domain phosphoinositide-interacting protein 3. Component of the autophagy machinery that controls the major intracellular degradation process by which cytoplasmic materials are packaged into autophagosomes and delivered to lysosomes for degradation. In the cellular response to starvation, may also function together with the TSC1-TSC2 complex and RB1CC1 in the inhibition of the mTORC1 signaling pathway
ZFYVE1	ZADH2	
